# Selection among site-dependent structurally constrained substitution models of protein evolution by approximate Bayesian computation

**DOI:** 10.1093/bioinformatics/btae096

**Published:** 2024-02-19

**Authors:** David Ferreiro, Catarina Branco, Miguel Arenas

**Affiliations:** CINBIO, Universidade de Vigo, 36310 Vigo, Spain; Department of Biochemistry, Genetics and Immunology, Universidade de Vigo, 36310 Vigo, Spain; CINBIO, Universidade de Vigo, 36310 Vigo, Spain; Department of Biochemistry, Genetics and Immunology, Universidade de Vigo, 36310 Vigo, Spain; CINBIO, Universidade de Vigo, 36310 Vigo, Spain; Department of Biochemistry, Genetics and Immunology, Universidade de Vigo, 36310 Vigo, Spain

## Abstract

**Motivation:**

The selection among substitution models of molecular evolution is fundamental for obtaining accurate phylogenetic inferences. At the protein level, evolutionary analyses are traditionally based on empirical substitution models but these models make unrealistic assumptions and are being surpassed by structurally constrained substitution (SCS) models. The SCS models often consider site-dependent evolution, a process that provides realism but complicates their implementation into likelihood functions that are commonly used for substitution model selection.

**Results:**

We present a method to perform selection among site-dependent SCS models, also among empirical and site-dependent SCS models, based on the approximate Bayesian computation (ABC) approach and its implementation into the computational framework *ProteinModelerABC*. The framework implements ABC with and without regression adjustments and includes diverse empirical and site-dependent SCS models of protein evolution. Using extensive simulated data, we found that it provides selection among SCS and empirical models with acceptable accuracy. As illustrative examples, we applied the framework to analyze a variety of protein families observing that SCS models fit them better than the corresponding best-fitting empirical substitution models.

**Availability and implementation:**

*ProteinModelerABC* is freely available from https://github.com/DavidFerreiro/ProteinModelerABC, can run in parallel and includes a graphical user interface. The framework is distributed with detailed documentation and ready-to-use examples.

## 1 Introduction

Substitution model selection is a traditional step of the phylogenetics pipeline because the applied substitution model can affect the accuracy of phylogenetic tree and ancestral sequence reconstructions, among other evolutionary inferences (Yang *et al.* 1994, Zhang and Nei 1997, Zhang 1999, Minin *et al.* 2003, Lemmon and Moriarty 2004, Keane *et al.* 2006, Ripplinger and Sullivan 2010, Cox and Foster 2013, Arenas and Bastolla 2019, Del Amparo and Arenas 2022, 2023). This need of selection among substitution models is essentially based on the heterogeneous evolutionary processes observed in nature at the molecular level, where genomic regions (Arbiza *et al.* 2011, Pandey and Braun 2020) and even protein sites (Pupko *et al.* 2002, Robinson *et al.* 2003, Echave *et al.* 2016, Jiménez-Santos *et al.* 2018, Neverov *et al.* 2021) often evolve under different selection pressures that better fit with different substitution models. As a consequence, a variety of substitution models of molecular evolution are available and used in the field (see for a review, Arenas 2015a).

At the protein level, two main types of substitution models were developed so far. First, the empirical substitution models, which consist of the relative rates of change among amino acids (exchangeability matrix) and the amino acid frequencies at the equilibrium (Thorne 2000, Yang 2006, Arenas 2015a). These models are traditionally obtained from large empirical datasets of nuclear (Jones *et al.* 1992, Whelan and Goldman 2001), chloroplast (Adachi *et al.* 2000), mitochondrial (Yang *et al.* 1998, Abascal *et al.* 2007) or virus (Nickle *et al.* 2007, Dang *et al.* 2010, Del Amparo and Arenas 2022) proteins, among other biological groups (Thorne 2000, Yang 2006, Arenas 2015a). Empirical substitution models of molecular evolution assume site-independent evolution and also that all the protein sites are modeled with the same exchangeability matrix and amino acid frequencies, which allow them a straightforward implementation into likelihood functions (where the likelihood is site-specific and site-independent) (Yang 2006, Puller *et al.* 2020) and, in extension, into likelihood-based phylogenetic methods (e.g. Darriba *et al.* 2011, Kozlov *et al.* 2019, Tamura *et al.* 2021). However, those assumptions could also produce proteins with unrealistic amino acid distributions and folding stability (Keane *et al.* 2006, Bordner and Mittelmann 2014, Arenas *et al.* 2015b, Arenas and Bastolla 2019). Second, the structurally constrained substitution (SCS) models, which directly consider selection on the protein structure (see for a review Liberles *et al.* 2012). Some SCS models account for site-dependent evolution and can produce proteins with amino acid distributions and folding stability more realistic than those obtained with empirical substitution models (Arenas *et al.* 2013). This is hardly surprising because, for example, it is known that residues at the protein core can exhibit substitution patterns different from those located in other regions of the protein (i.e. surface) due to selection on the protein folding stability and activity (Jiménez-Santos *et al.* 2018, Echave 2019, Perron *et al.* 2019). Indeed, physicochemical interactions between amino acids located at different protein sites are often observed (Shakhnovich *et al.* 1996) and can promote coevolution among sites (Starr and Thornton 2016, Neverov *et al.* 2021, Chaurasia and Dutheil 2022), suggesting that site-dependent substitution models could be preferred to those that ignore coevolution although this should be formally evaluated for every studied data. However, site-dependent models cannot be incorporated into likelihood functions due to the consideration of the site-dependence evolutionary process [notice that current likelihood-based phylogenetic methods calculate site-independent likelihoods (Yang 2006)]. Consequently, these models cannot be compared with other models through the traditional methods for substitution model selection based on likelihoods such as the likelihood ratio test, Akaike Information Criterion and Bayesian Information Criterion, among others (Sullivan and Joyce 2005, Luo *et al.* 2010, Darriba *et al.* 2011, 2020). As a consequence, there is a need of likelihood-free methods to perform selection among substitution models of evolution that include substitution models accounting for site-dependent evolution. Importantly, site-dependent SCS models can be used to study protein evolution by likelihood-free methods like those based on computer simulations, including applications for hypothesis testing (Bordner and Mittelmann 2014, Shah *et al.* 2015, Pascual-García *et al.* 2019, Del Amparo *et al.* 2023), validation of analytical frameworks (Arenas *et al.* 2017, Arenas and Bastolla 2019), and estimation of evolutionary parameters (Bastolla and Arenas 2019, Arenas 2022).

In this regard, as an alternative, the approximate Bayesian computation (ABC) approach is traditionally used to perform model selection in population genetics and ecology without the need of a likelihood function (Beaumont *et al.* 2002, Beaumont 2010). This approach is based on extensive computer simulations with parameters sampled from prior distributions, summary statistics that extract the information from the query and simulated data and a statistical adjustment (i.e. rejection or multiple linear regression, among others) to obtain the posterior distribution (probability) of the fitting of each evaluated model with the query data (Csilléry *et al.* 2012). Despite ABC does not require likelihood analyses, it can provide estimates with similar (sometimes higher) accuracy compared to those obtained with some likelihood-based methods (Lopes *et al.* 2014). Some previous works demonstrated that ABC can be used to study molecular evolution (Wilson *et al.* 2009, Lopes *et al.* 2014, Arenas *et al.* 2015a, Arenas 2015b, Moshe *et al.* 2022). For example, at the protein level, we previously applied ABC to estimate substitution and recombination rates with acceptable accuracy (Arenas 2022). Key factors for adapting ABC to the evolutionary analysis of protein sequences are the simulation of protein data along evolutionary histories (i.e. phylogenetic trees) under substitution models of evolution and, the design of informative summary statistics to extract evolutionary information from this genetic marker. Concerning the simulation of protein evolution upon evolutionary histories, it was implemented in diverse evolutionary frameworks (see the reviews Arenas 2012, Hoban *et al.* 2012) although only a few of them include SCS models (Grahnen and Liberles 2012, Arenas *et al.* 2013). We believe that efforts should still be made in implementing SCS models into computer simulators and, in general, into practical phylogenetic frameworks. Concerning the summary statistics to extract evolutionary information from protein sequences, Arenas (2022) found that several statistics (i.e. mean, standard deviation, skewness and kurtosis) of heterozygosity and pairwise sequence identity are informative for ABC-based analyses of protein evolution under empirical substitution models. However, SCS models produce evolutionary signatures in sequences that could only be detected by evaluating the fitting of the protein sequence with a respective protein structure. Conveniently, there are statistics that could be used for this purpose such as hydrophobicity (Jiménez-Santos *et al.* 2018), entropy (Goldstein and Pollock 2017), contact interactions (Franzosa and Xia 2009), solvent accessibility (Yeh *et al.* 2014) and, in general, protein folding stability. We believe that these statistics could allow the application of ABC to study patterns of protein evolution with selection on the protein structure.

Here, we present the application of ABC to perform selection among substitution models of protein evolution that can include site-dependent evolution, thus providing an alternative strategy to evaluate substitution models that, due to their complexity, cannot be implemented into likelihood functions. We implemented the method into a user-friendly computational framework called *ProteinModelerABC* that showed an acceptable accuracy in distinguishing among these models. As illustrative practical examples, we applied the framework to study the fitting of different site-dependent SCS and empirical models with protein families from diverse organisms of general interest.

## 2 Materials and methods

**Figure 1.**
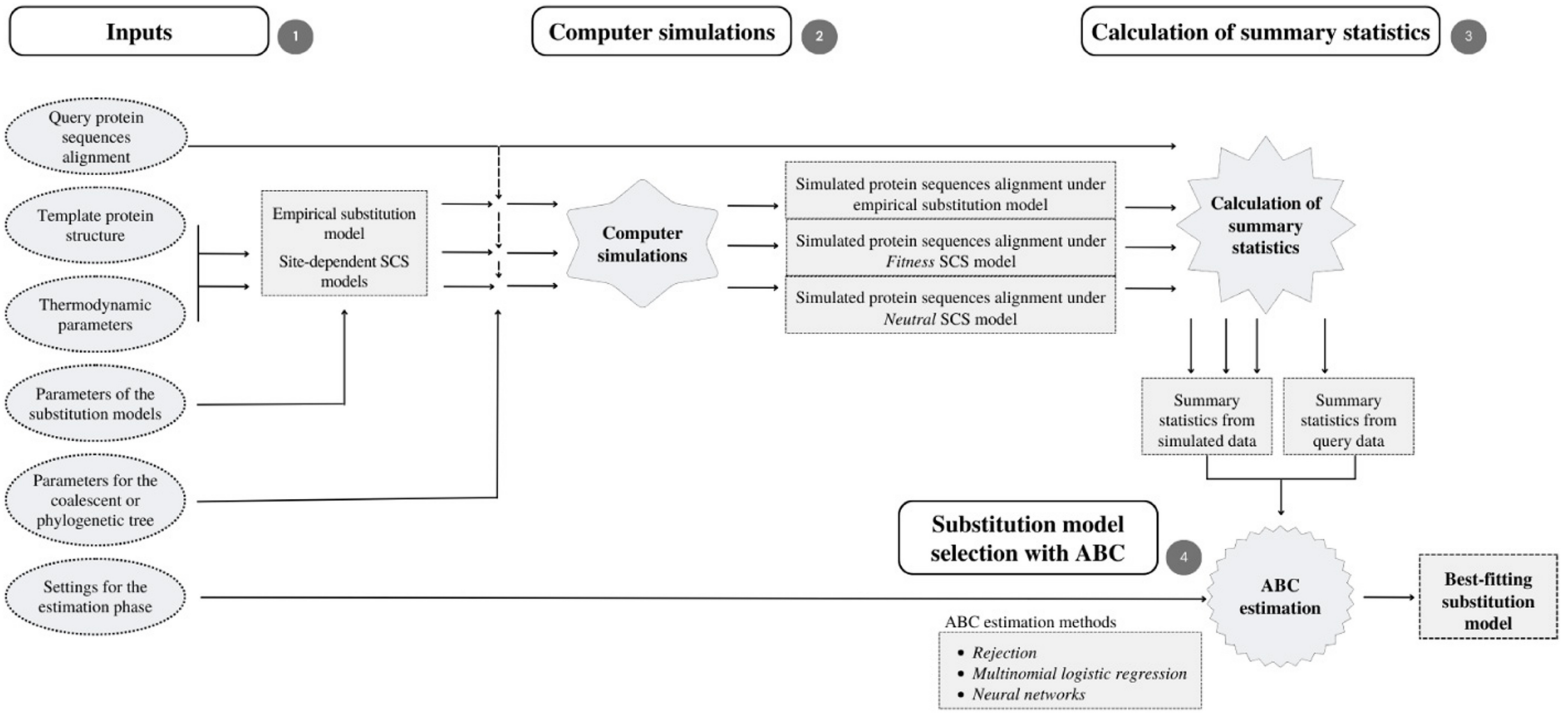
Pipeline of substitution model selection with *ProteinModelerABC*. The framework starts reading the query alignment of protein sequences and diverse user-specified information such as the substitution models to be evaluated and their parameters (Supplementary Table S1), the evolutionary history (simulated with the coalescent or a user-specified phylogenetic tree), the number of simulations and the ABC estimation method, among others. Next, the framework simulates protein evolution under the specified substitution models, obtaining a same number of simulations under each substitution model. In a subsequent step, it calculates the summary statistics for the query and simulated data. Finally, the framework predicts the best-fitting substitution model, among the studied substitution models, according to the posterior probabilities estimated with the ABC method.

We present an ABC-based method to identify the best-fitting substitution model for a given alignment of protein sequences through a few methodological steps that include the reading of input information, simulation of protein evolution along evolutionary histories under the studied substitution models, calculation of informative summary statistics and, estimation of posterior probabilities for the studied substitution models with common statistical ABC methods (Fig. 1). Details about these steps are presented below.

1. Input information. The ABC approach requires making some decisions, such as the number of simulations and the fraction of simulations retained for the estimation (tolerance), that could affect the results. Thus, in addition to the query multiple sequence alignment, the input information includes parameters of the evaluated substitution models of protein evolution, the underlined evolutionary histories and statistical ABC estimation methods (a list of all the parameters implemented in the framework is presented in Supplementary Table S1). A variety of input parameters are optional (including fixed and nuisance parameters, the latter allow user-specified prior distributions) and could provide a more realistic modeling of certain evolutionary processes. For example, the user can optionally specify population genetics parameters to simulate coalescent evolutionary histories (i.e. population size and population growth rate) and the empirical substitution models can include variation of the substitution rate among sites according to a Gamma distribution (Yang *et al.* 1994) and a proportion of invariable sites (Shoemaker and Fitch 1989). Despite the main aim of *ProteinModelerABC* is to evaluate the fitting of site-dependent SCS models [note that other well-established methods and frameworks are already available to identify the best-fitting substitution model among a set of empirical substitution models (i.e. Keane *et al.* 2006, Darriba *et al.* 2011, Kalyaanamoorthy *et al.* 2017, Darriba *et al.* 2020)], it implements a variety of empirical substitution models that allow diverse comparisons between site-dependent SCS and empirical models. In particular, the empirical substitution models implemented in *ProteinModelerABC* are *Blosum62* (Henikoff and Henikoff 1992), *CpRev* (Adachi *et al.* 2000), *Dayhoff* (Dayhoff *et al.* 1978), *DayhoffDCMUT* (Kosiol and Goldman 2005), *HIVb* (Nickle *et al.* 2007), *HIVw* (Nickle *et al.* 2007), *JTT* (Jones *et al.* 1992), *JonesDCMUT* (Kosiol and Goldman, 2005), *LG* (Le and Gascuel 2008), *Mtart* (Abascal *et al.* 2007), *Mtmam* (Yang *et al.* 1998), *Mtrev24* (Adachi and Hasegawa 1996), *RtRev* (Dimmic *et al.* 2002), *VT* (Müller and Vingron 2000), and *WAG* (Whelan and Goldman 2001), also any other exchangeability matrix and amino acid frequencies given as input could be evaluated. Concerning site-dependent SCS models, the framework implements two main site-dependent SCS models, hereafter named “*Neutral*” and “*Fitness*” (Arenas *et al.* 2013), that consider the stability of the native state with respect to both unfolding and misfolding states (Minning *et al.* 2013). The stability includes configurational entropies, hydrophobicity, and site-specific contacts [involving a statistical mechanical treatment of misfolded conformations that is computationally affordable (Bastolla *et al.* 2005a, 2005b)], among other physicochemical properties (Arenas *et al.* 2013). Misfolding stability affects the energy of amino acid contacts found in alternative structures (named as negative design to distinguish it from the positive design of protein stability based on native interactions) (Berezovsky *et al.* 2007, Noivirt-Brik *et al.* 2009, Minning *et al.* 2013) and it is important because, if only unfolded states are considered, the modeling of selection tends to artificially increase hydrophobicity (Arenas *et al.* 2013, Jiménez-Santos *et al.* 2018). The *Neutral* model is more general than the *Fitness* model. The *Neutral* model considers the fitness as a binary variable where all protein variants with stability above a given threshold (based on a representative protein structure) are considered viable (and equally fit) whereas all protein variants below the threshold are considered lethal and, therefore, discarded (Arenas *et al.* 2013). Thus, the *Neutral* model is less sensitive to variations of entropy and thermodynamic temperature (Arenas *et al.* 2013). Next, the *Fitness* model additionally considers that the probability of mutations depends on the effective population size, thus showing segregating variation in a population (Arenas *et al.* 2013). In this case, the fitness is an increasing function of stability and proportional to the fraction of protein variants in the native state (Goldstein 2011, 2013). In addition, the probability of accepting mutation events also considers the effective population size through the Moran’s birth–death process (Ewens 1979, Sella and Hirsh 2005). Thus, the *Neutral* model includes fewer parameters than the *Fitness* model and the *Fitness* model can be reduced to the *Neutral* model at a low thermodynamic temperature where the fitness tends to be 1 in highly stable proteins and zero in highly unstable proteins (Arenas *et al.* 2013). In practice, these models can produce different distributions of amino acid frequencies and folding stability of the modeled proteins. The *Fitness* model produced amino acid distributions more similar to the real observations than those obtained with the *Neutral* model for some protein families, while the *Neutral* model was in general more robust than the *Fitness* model to analyze diverse data (Arenas *et al.* 2013). For further information about the *Neutral* and *Fitness* site-dependent substitution models, we refer the reader to Arenas *et al.* (2013). As input information, these SCS models require the specification of several thermodynamic parameters and a protein structure (i.e. available from the Protein Data Bank, PDB) representative of the query alignment of protein sequences (Supplementary Table S1). Conveniently, the framework is distributed with a detailed documentation that includes recommendations for the specification of the input parameters.
2. Computer simulations. The computer simulations of protein data are performed in *ProteinModelerABC* with a recent version of the simulator *ProteinEvolver* (Arenas *et al.* 2013) adapted to ABC (Arenas 2022). Protein evolution is simulated along evolutionary histories that can be previously simulated with the coalescent (Kingman 1982) under diverse population genetics scenarios (Supplementary Table S1) or specified through an input phylogenetic tree. While the latter considers a same phylogenetic tree for all the simulations, the former can include stochasticity to obtain different coalescent evolutionary histories among simulations. The simulation of protein evolution under the studied substitution models is performed forward in time, from the root node to the tip nodes of the evolutionary history. Conveniently, *ProteinModelerABC* can run the simulations in parallel on a multicore machine to reduce computer time (Supplementary Fig. S1). As expected, a simulation of protein evolution under an empirical substitution model is more rapid (less than a second) than a simulation under a site-dependent SCS model (from seconds to minutes depending on the protein length and sample size) due to the consideration of structural constraints (Supplementary Fig. S2).
3. Summary statistics. We designed seven summary statistics (SS; details below and in Supplementary Table S2) that showed sufficient evolutionary information to distinguish between the implemented SCS models and between SCS and empirical models (details shown in the following section). In general, these SS comprise the protein folding stability, molecular diversity and physicochemical properties of the amino acids involved in the replacements. Concerning the protein folding stability, we included the mean and standard deviation of the free energy predicted with the framework *DeltaGREM* (Minning *et al.* 2013, Arenas *et al.* 2015b). As a measure of molecular diversity, we considered the number of segregating sites, following previous ABC studies of molecular evolution (Lopes *et al.* 2014, Arenas *et al.* 2015a, Arenas 2022). Additionally, we included the site-specific change of physicochemical properties among amino acids by the mean, standard deviation, skewness and kurtosis of the traditional Grantham distances (Grantham 1974).
4. Substitution model selection with ABC. The framework estimates the posterior probability of every studied substitution model with the query protein multiple sequence alignment using statistical methods available from the *abc* R library (Csilléry *et al.* 2012). In particular, the framework implements the rejection, multinomial logistic regression, and neural networks methods (Blum and François 2010, Csilléry *et al.* 2012). In addition to the posterior probabilities, the framework provides the confusion matrix (accuracy of predictions under every studied substitution model) and the goodness of fit of the studied substitution models with the query data. Indeed, the framework supplies distributions of the distance between SS of the retained simulations and SS of the query data for every studied substitution model, which illustrate about the realism of the modeling.
  Altogether, *ProteinModelerABC* provides selection among substitution models of protein evolution including complex models that cannot be implemented in likelihood functions through ABC. The framework is written in Python, C, and R, and can run in parallel on local or cluster computers. Interestingly, the program includes a graphical user interface that can be useful for users that are not familiar with the command line. *ProteinModelerABC* is freely available from https://github.com/DavidFerreiro/ProteinModelerABC and it is distributed with a detailed documentation and illustrative practical examples.

## 3 Results

### 3.1 *ProteinModelerABC* validation

The use of ABC for selecting among evolutionary scenarios is well-established in population genetics and ecology (e.g. Leuenberger and Wegmann 2010, Sousa *et al.* 2012, Branco *et al.* 2022) and we believe that it can provide a proper likelihood-free alternative to evaluate complex substitution models of molecular evolution. Here, we evaluated the accuracy of *ProteinModelerABC* to perform selection among empirical and SCS models under different scenarios: (i) Number of simulations for training the method (10 000, 50 000, and 100 000), (ii) tolerance (0.005, 0.01, and 0.05), and (*iii*) ABC estimation method including rejection, multinomial logistic regression, and neural networks. We performed the evaluations using data simulated under the *Dayhoff* empirical substitution model (which is widely used in the field), the *Fitness* site-dependent SCS model and the *Neutral* site-dependent SCS model. The simulations were inspired in the thioredoxin protein family [27 sequences and 316 amino acids (*l*) with sequence identity of 0.44; Pfam code PF00070] and a representative protein structure (PDB code 1TDE) (Waksman *et al.* 1994) obtained by homology modeling with *SWISS-MODEL* (Arnold *et al.* 2006) from the consensus sequence. Next, we simulated protein sequence alignments upon coalescent evolutionary histories considering a population size (*N*) of 1000 individuals and a population substitution rate (*θ* = 4 *Nμl*, where *μ* is the substitution rate per site per generation) sampled from a uniform prior distribution between 0 and 500 that include values commonly observed in nature (e.g. Carvajal-Rodriguez, 2006, Lopes *et al.* 2014, Arenas, 2022). For every scenario (3 substitution models × 3 different numbers of simulations × 3 ABC tolerance levels × 3 ABC estimation methods = 81 scenarios), we evaluated the power of *ProteinModelerABC* to distinguish between the three substitution models by cross-validation based on 100 permutations (Csilléry *et al.* 2012). We found that the framework distinguishes between the studied substitution models with acceptable accuracy regardless of the number of simulations used for training the method, the tolerance level and the ABC statistical method used for the estimation (Supplementary Table S3).

**Figure 2.**
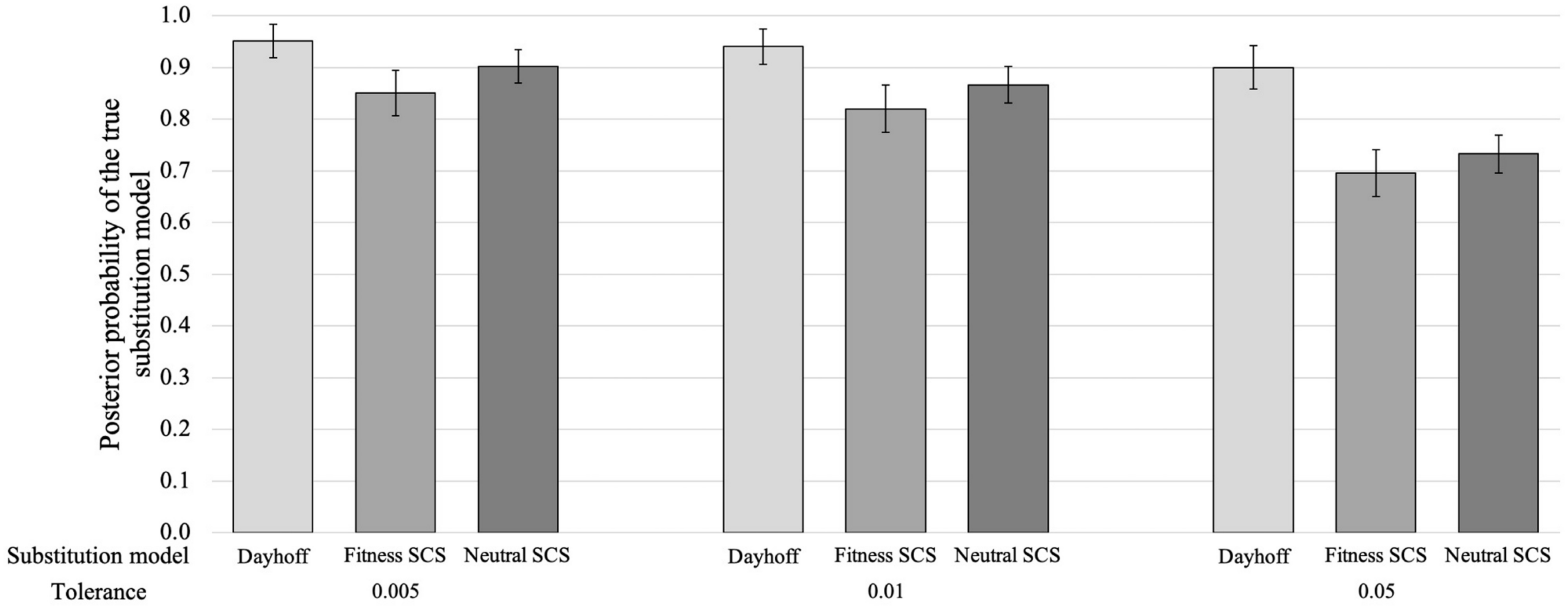
Evaluation of substitution model selection with *ProteinModelerABC* as a function of the tolerance. Posterior probability of the true substitution model (i.e. *Dayhoff*, site-dependent *Fitness* SCS and site-dependent *Neutral* SCS models) with the ABC rejection method, at different tolerance levels (0.005, 0.01, and 0.05) and using 10 000 training simulations, for 100 pseudo-observed datasets simulated under each substitution model. Error bars indicate 95% confidence intervals from the mean of the posterior probabilities of the true substitution model predicted for the pseudo-observed data. Estimates based on the multinomial logistic regression and neural networks methods are presented in Supplementary Fig. S3

Once we found that the method can distinguish between SCS and empirical models through cross-validation, we evaluated its accuracy in identifying the true substitution model in pseudo-observed (test) data. In particular, we simulated 100 alignments of protein sequences evolved under each studied substitution model (*Dayhoff*, *Fitness* SCS and *Neutral* SCS models) and for each simulated dataset we performed substitution model selection with *ProteinModelerABC*. These analyses were also performed considering 10 000, 50 000, and 100 000 training simulations, tolerance levels of 0.005, 0.01, and 0.05 and, the three ABC statistical methods. Again, we found that the accuracy of the substitution model selection is not affected by the number of simulations (Supplementary Fig. S3; compare the three plots) and thus 10 000 simulations are sufficient to distinguish between the studied models. Concerning the optimal tolerance, it varied among the studied ABC statistical methods (Supplementary Fig. S3). In particular, the rejection method showed a high robustness in predicting the true substitution model although its accuracy slightly decreased when increasing the tolerance (Fig. 2), a pattern not observed for substitution model selection with the multinomial logistic regression and neural networks methods (Supplementary Fig. S3). However, the latter methods could not converge when the tolerance is small (not enough retained simulations for the estimation) where at least a tolerance of 0.05 was required to obtain accurate estimates (Supplementary Fig. S3). Altogether, the rejection method was less sensitive to the tolerance for substitution model selection and thus we believe that it could be used by default. We did not observe effects of the studied number of simulations used for training the method on the accuracy of the estimates (Supplementary Fig. S3).

### 3.2 Illustrative examples of substitution model selection in diverse protein families

We used *ProteinModelerABC* to identify the best-fitting substitution model, among the best-fitting empirical substitution model previously selected with *ProtTest3* (Darriba *et al.* 2011) and the site-dependent SCS models implemented in *ProteinModelerABC*, in 10 different protein families (Table 1). These protein families belong to viruses related to human diseases including HIV-1 PR, HIV-1 gag, influenza NS1, SARS-CoV-2 endopeptidase C30 and 2'-O-methyltransferase, Ebola nucleoprotein and, the tumor necrosis factor (TNF) of monkeypox (Mpox) virus. Additionally, we analyzed the highly conserved intracellular signaling Toll-Interleukin protein domain, the squalene epoxidase and the mitochondria membrane translocase, all of them randomly selected but folding in known protein structures. We obtained the protein datasets from the Pfam (Mistry *et al.* 2021) and PROSITE (Sigrist *et al.* 2012) databases and they presented diverse sequence length (from 99 to 450 amino acids), sample size (from 8 to 128 sequences) and sequence identity (Table 1). Next, for every dataset, we aligned the sequences with *MUSCLE* (Edgar 2004) and also we obtained a consensus sequence that we used to identify a representative protein structure by homology modeling with *SWISS-MODEL* (Table 1). The simulation of protein evolution under site-dependent SCS models and the prediction of protein folding stability (free energy) require homology between the representative protein structure and the sequences of the dataset and thus, sites of the dataset without homology with the protein structure were excluded. Next, we ran *ProteinModelerABC* with 10 000 simulations under each studied substitution model and under a prior distribution for the substitution rate that produces simulated data with a distribution of sequence identity that includes the sequence identity of the real data (Table 1). Indeed, following results from the previous section, we performed the estimations with the rejection method under a tolerance of 0.005.

**Table 1.** Real protein families and their substitution model selection with *ProteinModelerABC*^a^.

Protein family	Sequences database entry	Number of sequences and sequences length	Sequence identity	Prior for the population substitution rate and derived range of sequence identity	Template protein structure	Best-fitting empirical substitution model	Posterior probabilities for substitution model selection		
Tumor necrosis factor monkeypox	GenBank accession codes^b^	10, 160	0.95	Uniform (0–100)	3on9	HIVw	Fitness	HIVw	**Neutral**
				(1.00–0.65)			0.22	0.01	**0.77**
HIV protease (PR)	PS50175	95, 99	0.91	Uniform (0–150)	1tcx	HIVb	**Fitness**	HIVb	Neutral
				(1.00–0.46)			**0.45**	0.33	0.22
HIV gag polyprotein	PF00540	128, 288	0.69	Uniform (0–500)	1l6n	RtRev	**Fitness**	Neutral	RtRev
				(1.00–0.41)			**0.59**	0.34	0.07
Influenza NS1	PF00600	25, 202	0.83	Uniform (0–200)	4oph	JTT	Fitness	JTT	**Neutral**
				(1.00–0.54)			0	0.13	**0.87**
Coronavirus endopeptidase C30	PF05409	30, 299	0.53	Uniform (0–500)	1lvo	LG	**Fitness**	LG	Neutral
				(1.00–0.42)			**0.95**	0	0.05
Coronavirus 2'-O-methyltransferase	PF06460	28, 298	0.62	Uniform (0–500)	7c2i	LG	**Fitness**	LG	Neutral
				(1.00–0.42)			**0.51**	0.3	0.19
Toll-Interleukin receptor domain	PF01582	23, 171	0.3	Uniform (0–700)	5ku7	WAG	**Fitness**	Neutral	WAG
				(1.00–0.25)			**0.97**	0.03	0
Mitochondria membrane translocase	PF08038	54, 50	0.51	Uniform (0–500)	6ucv	WAG	Fit ness	**Neutral**	WAG
				(1.00–0.14)			0.36	**0.44**	0.2
Squalene epoxidase	PF08491	12, 450	0.66	Uniform (0–500)	6c6n	WAG	**Fitness**	Neutral	WAG
				(1.00–0.50)			**0.97**	0.03	0
Ebola nucleoprotein	PF05505	8, 373	0.67	Uniform (0–500)	6c54	LG	Fitness	LG	**Neutral**
				(1.00–0.47)			0	0.02	**0.98**

a For every studied protein family, the table shows the Pfam or PROSITE accession code (excepting for the first dataset where GenBank accession codes are shown in the table foot), the number of sequences and the sequence length, the sequence identity (average of pairwise sequence identities), the prior for the population substitution rate (including the derived approximate range of average pairwise sequence identity), a representative protein structure (PDB code), the best-fitting empirical substitution model selected with *ProtTest3* and the posterior probability of every studied substitution model (empirical, site-dependent Fitness SCS, and site-dependent Neutral SCS models) with *ProteinModelerABC* (the posterior probability of the selected model is shown in bold).

b AAB94354, AAB94356, AAB94388, ADZ29547, YP_010085450, AXN75227, AIE41152, AAB94364, URF91555, and AAB94363.

The goodness of fit analysis showed that, in general, the SS of the real data fall within the SS of the retained simulated data especially for the best-fitting substitution model (illustrative examples are shown in Supplementary Fig. S4). In general, we found that site-dependent SCS models are preferred by most of the summary statistics (in terms of distance to the observed summary statistics) compared to the traditional empirical substitution models (Supplementary Table S4). Indeed, for all the studied real datasets, we found that the site-dependent SCS models fitted better with the real data than the best-fitting empirical substitution model selected with *ProtTest3* (Table 1).

## 4 Discussion

Current methods for substitution model selection are based on the likelihood of fitting the substitution models with the query data. This likelihood is commonly calculated per site, assuming site-independent evolution (Yang 2006, Puller *et al.* 2020), and none current likelihood function allows the evaluation of substitution models that consider site-dependent evolution. However, these models are increasing in popularity because can produce proteins with more realistic folding stability and distribution of amino acid frequencies than traditional substitution models (i.e. Robinson *et al.* 2003, Rodrigue *et al.* 2005, Yu and Thorne 2006, Arenas *et al.* 2013, Larson *et al.* 2020) and could be used to analyze protein evolution for diverse applications such as hypothesis testing (Bordner and Mittelmann 2014, Shah *et al.* 2015, Pascual-García *et al.* 2019, Del Amparo *et al.* 2023), validation of analytical frameworks (Arenas *et al.* 2017, Arenas and Bastolla 2019), and estimation of evolutionary parameters (Bastolla and Arenas 2019, Arenas 2022). Next, the implementation of these substitution models in likelihood-free sampling methodologies such as Monte Carlo and ABC (Beaumont *et al.* 2002, Rodrigue *et al.* 2005) becomes relevant for extending their practical applications, including substitution model selection. Here, we present the application of the ABC approach to perform selection among substitution models that can include site-dependent evolution, thus without using likelihood. In particular, we extended our previous ABC studies oriented to estimate parameters of molecular evolution (Arenas *et al.* 2015a, Arenas 2022), by adapting simulations and summary statistics, to the selection among substitution models of protein evolution that can consider evolutionary constraints from the protein structure. We found that this ABC framework for selection among complex substitution models presents an acceptable accuracy using the implemented set of summary statistics (which was sufficiently informative to distinguish among data simulated under empirical and SCS models, Supplementary Table S4 and Supplementary Fig. S4), and it is especially robust through the implemented ABC rejection method [the implemented logistic and neural networks methods were highly sensible to the tolerance parameter, requiring a high tolerance to ensure accurate model selection as discussed in Beaumont (2010), Fig. 2, and Supplementary Fig. S3]. A low level of tolerance can be convenient to obtain accurate estimates under the rejection method (Sunnåker *et al.* 2013), as we also found exploring low tolerance levels [i.e. 0.005 and 0.01, the latter was also recommended in previous studies (Csilléry *et al.* 2012, Nunes and Prangle 2015), Fig. 2]. Regarding the number of simulations, we found that 10 000 simulations can be sufficient to distinguish between the studied substitution models (Supplementary Fig. S3), which is a lower number of simulations than that required to estimate parameters in our previous studies [50 000 simulations in Arenas (2022)] although this is not surprising since parameters estimation usually requires more simulations than model selection.

We implemented the method into a freely available framework named *ProteinModelerABC* that includes flexibility concerning the underlined evolutionary history (simulated with the coalescent under diverse population genetics processes or specified by the user as a phylogenetic tree, Supplementary Table S1), the modeling of protein evolution (a variety of substitution models are implemented) and the ABC statistical methods to calculate the posterior probability of every studied substitution model with the query data. Next, for subsequent evolutionary analyses, similarly to likelihood-based methods for substitution model selection (Darriba *et al.* 2011, 2020), we recommend applying the selected best-fitting substitution model, which is the model presenting the highest posterior probability with the study data. The framework can run on the command line of local and cluster computers and includes a graphical user interface (GUI). The required computer time varies depending on the size of the studied data (Supplementary Fig. S2) and, conveniently, the simulations can run in parallel (on both command line and GUI versions) on a multicore machine to reduce the computer time (Supplementary Fig. S1). *ProteinModelerABC* is distributed with a detailed documentation and several illustrative examples that we recommend exploring.

As illustrative examples of application, we investigated the selection between site-dependent SCS models and the best-fitting empirical substitution model (previously selected with *ProtTest3*) in diverse protein families of general interest (Table 1). For all the studied real data, we found that site-dependent substitution models explained the real protein evolution better than the best-fitting empirical substitution models. Perhaps the currently available set of empirical substitution models is very limited and more empirical substitution models should be developed to better mimic the evolution of the studied protein families. However, we believe that the main cause of these findings is that site-dependent SCS models are much more specific and realistic than the empirical substitution models because, as indicated in the introduction, the empirical substitution models are usually too generalist, assume a same exchangeability matrix for all the protein sites and ignore coevolution. Note that proteins often present intramolecular interactions that can promote selection toward specific variants through site-dependent evolution (Woo *et al.* 2014, Rawi *et al.* 2015, Codoñer *et al.* 2017, Priya and Shanker 2021, Ferreiro *et al.* 2022). On the other hand, we find important to mention that the currently implemented site-dependent SCS models assume a representative protein structure for all the sequences of the query data. This could lead to a poor fitting if the query data has sequences poorly represented by the cited protein structure. In this regard, a representative protein structure can be selected from the studied protein sequences by diverse methods (Kuhlman and Bradley 2019) such as the traditional homology modeling [especially when there are protein structures in databases such as PDB likely to resemble the structure of the study sequences (Hameduh *et al.* 2020)], and the recent deep learning methods implemented in *AlphaFold* and *RoseTTAFold* (Baek *et al.* 2021, Jumper *et al.* 2021) that are particularly useful when there are not protein structures in databases fitting with the study sequences. Indeed, protein sites present in the study sequences but not in the structure, or disordered, could reduce the fitting of the SCS model with the study sequences due to a lack of structural information in those sites (Kolchanov *et al.* 1983, Zhang *et al.* 2011), suggesting a careful modeling and refinement for example also with deep learning methods (Nguyen *et al.* 2019, Jing and Xu 2021). We believe that the development of more robust SCS models, such as SCS models that consider the evolution and diversity of protein structures, and their implementation into useful frameworks for phylogenetic analyses, are highly demanded in the field to provide more reliable evolutionary estimates. Altogether, we show that ABC can provide a free-likelihood alternative for selecting among complex substitution models of evolution that are often more realistic than the traditional empirical substitution models.

## Supplementary Material

btae096_Supplementary_Data

## Data Availability

*ProteinModelerABC* is freely available from https://github.com/DavidFerreiro/ProteinModelerABC. The simulated and real data used in the study are available from Zenodo at https://doi.org/10.5281/zenodo.10491125.
