# Supplementary material for: Selection among site-dependent structurally constrained substitution models of protein evolution by approximate Bayesian computation
Source: Bioinformatics. 2024 Feb 19;40(3):btae096. doi: 10.1093/bioinformatics/btae096 (PMC10914458; doi:10.1093/bioinformatics/btae096)
Supplement: btae096_Supplementary_Data [file btae096_supplementary_data.pdf]

Supplementary Material

Selection among site-dependent structurally constrained substitution models of protein evolution by approximate Bayesian computation

The supplementary material includes Tables S1-S4 and Figures S1-S4.

Supplementary tables

Table S1. Summary of input parameters for running *ProteinModelerABC*. For every parameter, the table includes a related methodological step (i.e., general analytical process, associated to the simulation of protein data, associated to the substitution model/s or associated to the ABC estimation), if the parameter is mandatory or optional to perform an analysis, and some useful information. Further details and recommendations for the specification of every parameter are provided in the documentation distributed with the framework.

Parameter	Method	Mandatory	Comments
Computer cluster enviroment information	General	Yes if Cluster version	Input file with user-specified computer cluster environment information
Query alignment of protein sequences	General	Yes	Input file in *phylip* sequential format.
Consideration of indels	General	Yes	How to consider indels (gaps). They can be ignored or considered as a new state.
Protein structure template	General	Yes	Representative protein structure (PDB file) of the studied multiple sequence alignment. It is required to predict folding free energies.
Protein structure chain	General	Yes	Chain of the representative protein structure. It is required to predict folding free energies.
Amount of output information shown on the screen	General	Yes	Amount of output information shown on the screen when running the program.
Number of simulations	Simulations	Yes	Total number of simulations.
Number of processors	Simulations	Yes	Number of processors used to run the simulations (note that the simulations can run in parallel).
Save simulations	Simulations	Yes	Save the simulated data in a compressed folder if desired.
Coalescent or phylogeny	Simulations	Yes	Indicate coalescent simulation (with user-specified population genetics parameters) or a rooted phylogenetic tree (user-specified), upon which protein evolution will be simulated.

Haploid/diploid	Simulations	Yes if coalescent	Indicate if the data belongs to a haploid or diploid organism.
Amino acid substitution rate	Simulations	Yes if coalescent	It can be fixed or sampled from a user-specified prior distribution.
Population size (*N*)	Simulations	Yes if coalescent	Effective population size.
Dated tips	Simulations	No	Time at which the tip nodes of the tree are sampled (in units of *2N* generations for diploids).
Generation time	Simulations	No	Time for each generation (in units of *2N* generations for diploids). It can be fixed or sampled from a uniform distribution.
Population growth rate	Simulations	No	The population size can change over time by an exponential growth rate.
Migration model	Simulations	No	Migration model and type of population structure.
Migration rate	Simulations	No	The migration rate among demes (subpopulations). It can be constant or variable over time.
Convergence demes	Simulations	No	Events of convergence of demes.
Phylogenetic tree	Simulations	Yes if phylogeny	User-specified phylogenetic tree.
Substitution models of amino acid evolution	Substitution model	Yes	Empirical and/or site-dependent SCS models to be evaluated.
Amino acid frequencies	Substitution model	Yes	Amino acid frequencies at the equilibrium.
Heterogeneity of the substitution rate among sites (+G)	Substitution model	No	Variation of the substitution rate among sites according to a Gamma distribution.
Proportion of invariable sites (+I)	Substitution model	No	Proportion of invariable sites in the sequences.
Thermodynamic temperature	Substitution model	Yes	Thermodynamic temperature used to predict protein folding stability.
Configurational entropy per residue (unfolded)	Substitution model	Yes	Configurational entropy per residue for unfolded protein states.

Configurational entropy per residue (misfolded)	Substitution model	Yes	Configurational entropy per residue for misfolded protein states.
Configurational entropy offset (misfolded)	Substitution model	Yes	Configurational entropy offset for misfolded protein states.
Third cumulant in REM calculation	Substitution model	Yes	The third cumulant in REM calculation to predict protein folding stability.
Population size considered for the SCS models	Substitution model	Yes if *Fitness* SCS model	The population size to evaluate protein states through the Moran model.
ABC iterations	Estimation	Yes	Number of permutations used for the cross validation.
ABC tolerance	Estimation	Yes	Fraction of simulations closer to the observed data that are retained for the ABC analysis.
ABC method	Estimation	Yes	Statistical ABC method used to estimate the posterior probability of every studied substitution model.
Summary statistics	Estimation	Yes	Selected summary statistics to perform the ABC analysis.
Output files with results in plots	Estimation	Yes	It provides informative PDF documents with multiple plots showing results.

Table S2. Summary statistics implemented in *ProteinModelerABC*. For every summary statistic the table includes an identification (ID) and a brief description. See the documentation of the framework for further details.

ID	Name	Description
**1**	*DGREM_mean*	Mean of folding stability (free energy) of the proteins of the dataset.
**2**	*DGREM_sd*	Standard deviation of folding stability among the proteins of the dataset.
**3**	*SegSites*	Number of segregating sites.
**4**	*Grantham_mean_Position*	Mean of the Grantham distance of amino acid replacements at every protein site (position).
**5**	*Grantham_sd_Position*	Standard deviation of the Grantham distance of amino acid replacements at every protein site.
**6**	*Grantham_sk_Position*	Skewness of the Grantham distance of amino acid replacements for every protein site.
**7**	*Grantham_ku_Position*	Kurtosis of the Grantham distance of amino acid replacements at every protein site.

Table S3. *ProteinModelerABC* cross-validation based on 100 permutations. Frequency of predicting the true substitution model for every studied substitution model (*Dayhoff*, site-dependent *Fitness* SCS and site-dependent *Neutral* SCS models) using each implemented ABC estimation method (rejection "*Rejection*", multinomial logistic regression *"Mnlogistic"* and neural networks *"Neuralnet"*) with different ABC tolerance (0.005, 0.01 and 0.05) and number of training simulations per studied substitution model (10000, 50000 and 100000).

Number of simulations	Tolerance	ABC estimation method	True model	Frequency (%) of predicting the true model (confusion matrix)		
				**Dayhoff**	**Fitness**	**Neutral**
10,000	0.005	*Rejection*	*Dayhoff*	93	99	96
			Fitness	91	95	96
			Neutral	92	94	88
		Mnlogistic	*Dayhoff*	95	100	95
			Fitness	95	95	95
			Neutral	96	97	98
		Neuralnet	*Dayhoff*	94	98	96
			Fitness	94	97	95
			Neutral	98	99	96
	0.01	*Rejection*	*Dayhoff*	93	97	86
			Fitness	92	96	95
			Neutral	89	99	95
		Mnlogistic	*Dayhoff*	97	97	98
			Fitness	97	98	97
			Neutral	96	94	96
		Neuralnet	*Dayhoff*	97	95	93
			Fitness	95	97	96
			Neutral	99	99	95
	0.05	*Rejection*	*Dayhoff*	91	97	79
			Fitness	89	98	79
			Neutral	84	94	89
		Mnlogistic	*Dayhoff*	96	94	94
			Fitness	97	96	91
			Neutral	95	95	95
		Neuralnet	*Dayhoff*	96	96	97
			Fitness	90	97	92
			Neutral	96	94	94
50,000	0.005	*Rejection*	*Dayhoff*	93	97	95

			Fitness	94	98	92
			Neutral	92	98	90
		Mnlogistic	*Dayhoff*	94	99	97
			Fitness	97	97	99
			Neutral	97	98	99
		Neuralnet	*Dayhoff*	97	98	96
			Fitness	100	98	96
			Neutral	97	95	97
	0.01	*Rejection*	*Dayhoff*	93	99	89
			Fitness	93	98	90
			Neutral	96	100	96
		Mnlogistic	*Dayhoff*	93	98	97
			Fitness	98	98	96
			Neutral	97	95	96
		Neuralnet	*Dayhoff*	94	96	96
			Fitness	98	97	98
			Neutral	97	99	95
	0.05	*Rejection*	*Dayhoff*	91	98	86
			Fitness	81	96	83
			Neutral	81	97	93
		Mnlogistic	*Dayhoff*	93	98	94
			Fitness	97	96	94
			Neutral	98	93	96
		Neuralnet	*Dayhoff*	92	99	94
			Fitness	97	96	97
			Neutral	98	96	96
100,000	0.005	*Rejection*	*Dayhoff*	93	97	95
			Fitness	94	98	92
			Neutral	92	98	90
		Mnlogistic	*Dayhoff*	94	99	97
			Fitness	97	97	99
			Neutral	97	98	99
		Neuralnet	*Dayhoff*	97	98	96
			Fitness	100	98	96
			Neutral	97	95	97
	0.01	*Rejection*	*Dayhoff*	93	99	89
			Fitness	93	98	90
			Neutral	96	100	96

	Mnlogistic	*Dayhoff*	93	98	97
		Fitness	98	98	96
		Neutral	97	95	96
	Neuralnet	*Dayhoff*	94	96	96
		Fitness	98	97	98
		Neutral	97	99	95
0.05	*Rejection*	*Dayhoff*	91	98	86
		Fitness	81	96	83
		Neutral	81	97	93
	Mnlogistic	*Dayhoff*	93	98	94
		Fitness	97	96	94
		Neutral	98	93	96
	Neuralnet	*Dayhoff*	92	99	94
		Fitness	97	96	97
		Neutral	98	96	96

Table S4. Distance between simulated (mode) and observed summary statistics for the studied real data. For every dataset, the table shows the distances between the simulated and observed summary statistics. For the simulated summary statistics the table indicates the most frequent values (mode) obtained from the 0.005 (tolerance) closest simulations to the real data and under the corresponding substitution models (empirical and site-dependent *Fitness* SCS and *Neutral* SCS models). The text in bold indicates the selected best-fitting substitution model and the closest simulated summary statistic to the observation. The last row presents a raking of the most informative summary statistics for every type of substitution model.

Protein family	Substitution model	*DGREM mean*	*DGREM sd*	*SegSites*	*Grantham distance mean position*	*Grantham distance sd position*	*Grantham distance sk position*	*Grantham distance ku position*
Tumor necrosis factor monkeypox	Fitness	0.17	0.0202	**58**	196783.30	177719	3.26	24.28
	HIVw	**0.10**	**0.0009**	115	**5545.71**	**11219**	1.41	12.80
	**Neutral**	0.13	0.0137	105	65685.87	88800	**0.09**	**5.66**
HIV protease	**Fitness**	0.094	0.06	40	6945424	7475843	1.93	11.42
	HIVb	**0.004**	**0.01**	**1**	**1340531**	**3757585**	3.91	19.24
	Neutral	0.133	0.03	47	11995939	13128529	**1.66**	**10.51**
HIV gag polyprotein	**Fitness**	0.053	**0.0003**	**57**	5772328	3227541	1.20	7.14
	Neutral	0.046	0.0150	62	**2812966**	**2226535**	**0.17**	**2.65**
	RtREV	**0.008**	0.0084	164	13205051	15361769	4.21	49.15
Influenza NS1	Fitness	**0.01**	0.0043	**43**	244201.30	196979.80	4.44	47.01
	JTT	0.03	**0.0036**	109	91493.32	48229.89	1.07	6.80
	**Neutral**	0.05	0.0099	55	**72804.97**	**198439**	**0.24**	**0.17**
Coronavirus endopeptidase C30	**Fitness**	0.33	-	**46**	797329.50	**258185.30**	**0.37**	**0.86**
	LG	0.03	-	62	1211228	748835.10	5.05	46.14
	Neutral	**0.01**	-	58	**1105742**	811997.80	3.53	33.41

Coronavirus 2'-O-methyltransferase	**Fitness**	0.12	0.0030	**53**	**134404.10**	228050.90	0.43	2.35
	LG	0.26	**0.0001**	56	413732.70	30705.02	**0.21**	**0.92**
	Neutral	**0.11**	0.0017	55	367162.70	**192606.90**	0.42	2.51
Toll-Interleukin receptor domain	**Fitness**	**0.10**	-	2	**5923.38**	202807.10	0.63	0.09
	Neutral	0.24	-	2	439396.7	197891.10	**0.52**	**0.03**
	WAG	0.22	-	**1**	90596.05	**24845.04**	0.60	0.96
Mitochondria membrane translocase	Fitness	0.04	0.028	11	8577530	2429583	0.33	1.13
	**Neutral**	0.03	**0.016**	11	**7969909**	**1693804**	**0.12**	**1.05**
	WAG	**0.02**	0.017	11	8299147	2566616	0.76	3.71
Squalene epoxidase	**Fitness**	**0.02**	0.014	191	167776.60	255842.70	1.88	6.85
	Neutral	0.04	0.011	188	**67489.20**	93922.36	0.47	2.49
	WAG	0.21	**0.005**	**162**	110010.30	**19275.10**	**0.42**	**1.51**
Ebola nucleoprotein	Fitness	**0.03**	**0.002**	**40**	32992.77	28250.01	2.58	22.33
	LG	0.04	0.009	110	**15594.76**	9098.45	1.27	9.08
	**Neutral**	0.17	0.003	156	87244.66	**43966.10**	**0.62**	**3.43**
Summary	*SCS model*	***6***	*3*	***6***	***7***	***6***	***8***	***8***
	Empirical model	*4*	***5***	*3*	*3*	*4*	*2*	*2*

Supplementary figures

Figure S1. Computer time required for running an analysis with *ProteinModelerABC* under a different number of cores. The dataset included 10 sequences of 160 amino acids (real dataset 1 in Table 1). The 10,000 simulations were carried out using a local machine (2.6 GHz Intel Core i7) under 1, 2, 4, 8 and 12 cores (A) and using a computer cluster (Intel Xeon Ice Lake 8352Y) under 50, 100, 200, 325 and 500 cores (B). The dataset included 10 sequences of 160 amino acids (real dataset 1 in Table 1). The decrease of computer time does not follow a straight line because some steps of the analysis cannot be parallelized and also the memory was shared among cores.

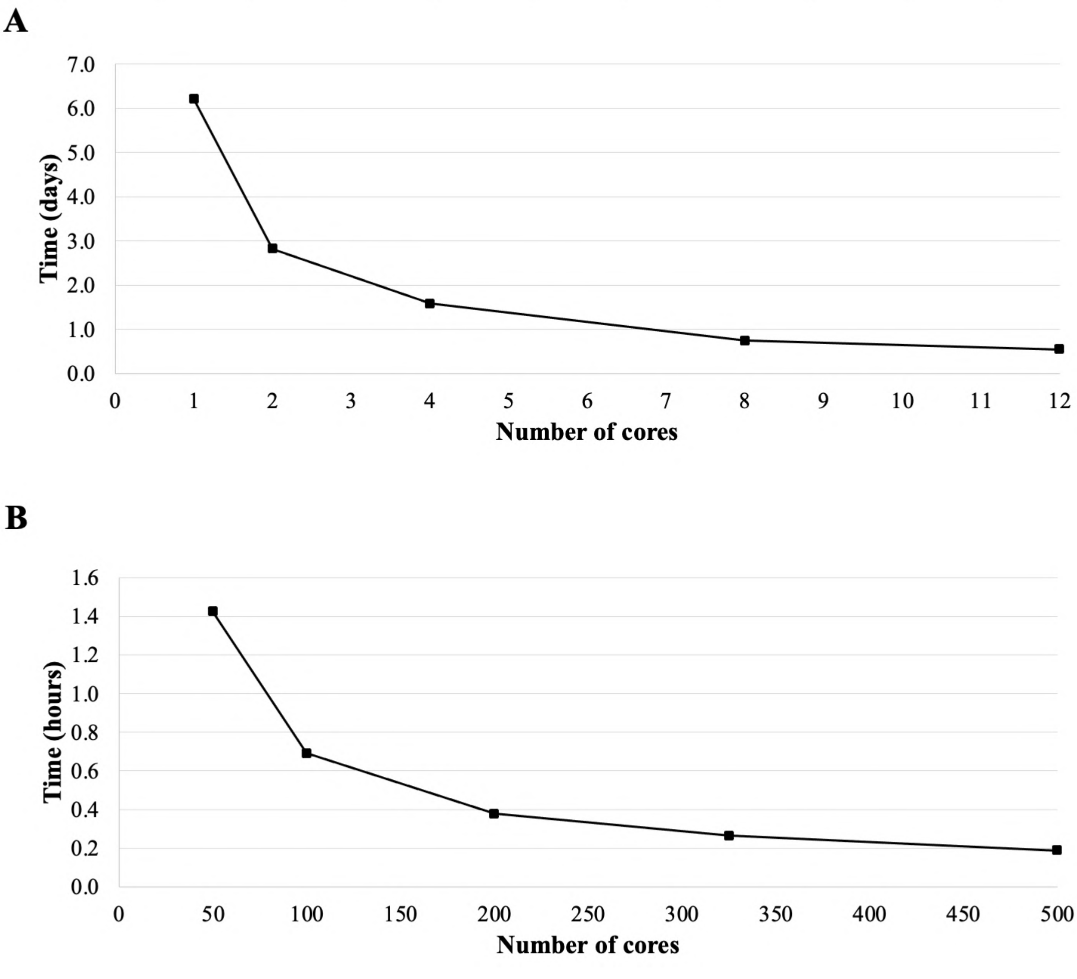

Figure S2. Influence of the size of input data and substitution models on the computer time. Above, the figure shows that the computer time increases as a function of the data size (number of sequences and sequences length) (A). Below, the figure shows the computer time required to simulate data under the empirical and structurally constrained substitution models used to study each real dataset (Table 1, following the order A-J) (B). The computer times were obtained from running the analyses on a computer cluster (Intel Xeon Ice Lake 8352Y) under 500 cores.

A

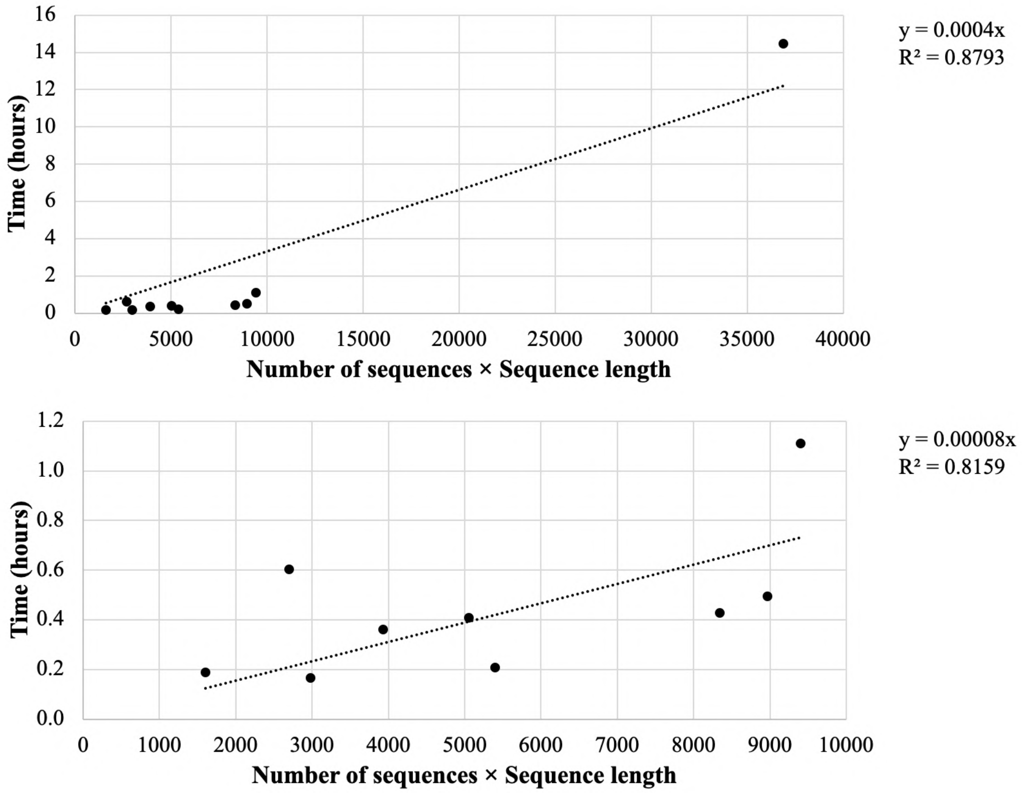

B

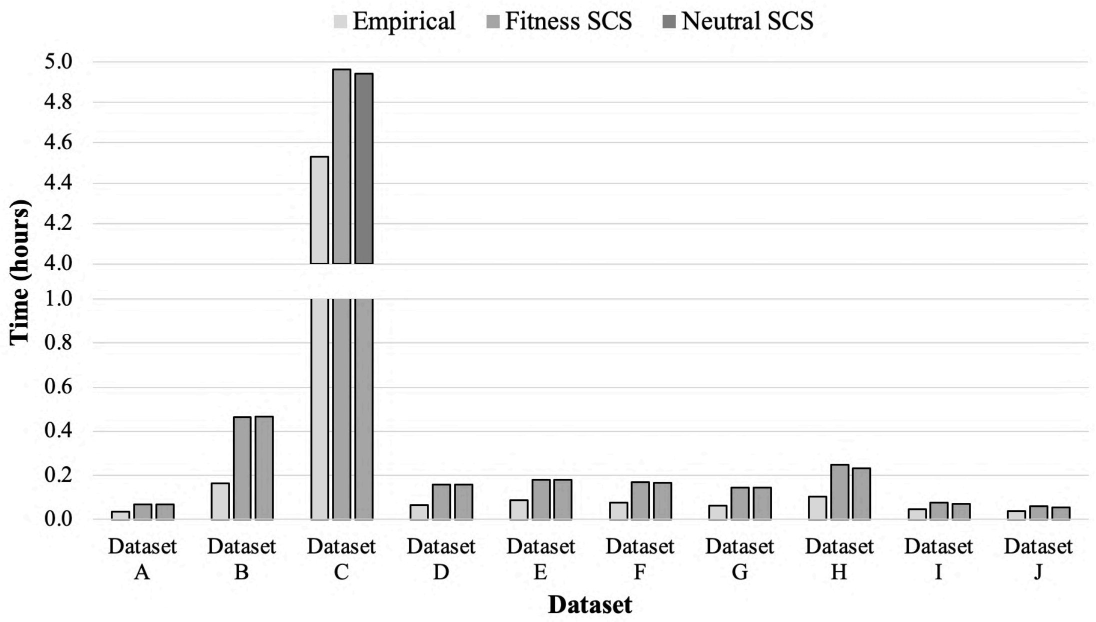

Figure S3. Evaluation of substitution model selection under diverse estimation conditions. Posterior probability of the true substitution model (*Dayhoff* and site-dependent *Fitness* and *Neutral* SCS models) with three ABC estimation methods (rejection, multinomial logistic regression and neural networks), under different tolerance levels (0.005, 0.01 and 0.05) and number of training simulations (10000, 50000 and 100000, which correspond with the upper, middle and lower plots, respectively), in 100 pseudo-observed datasets simulated under each substitution model. Error bars indicate 95% confidence intervals from the mean of the posterior probabilities of the true substitution model predicted for the pseudo-observed data.

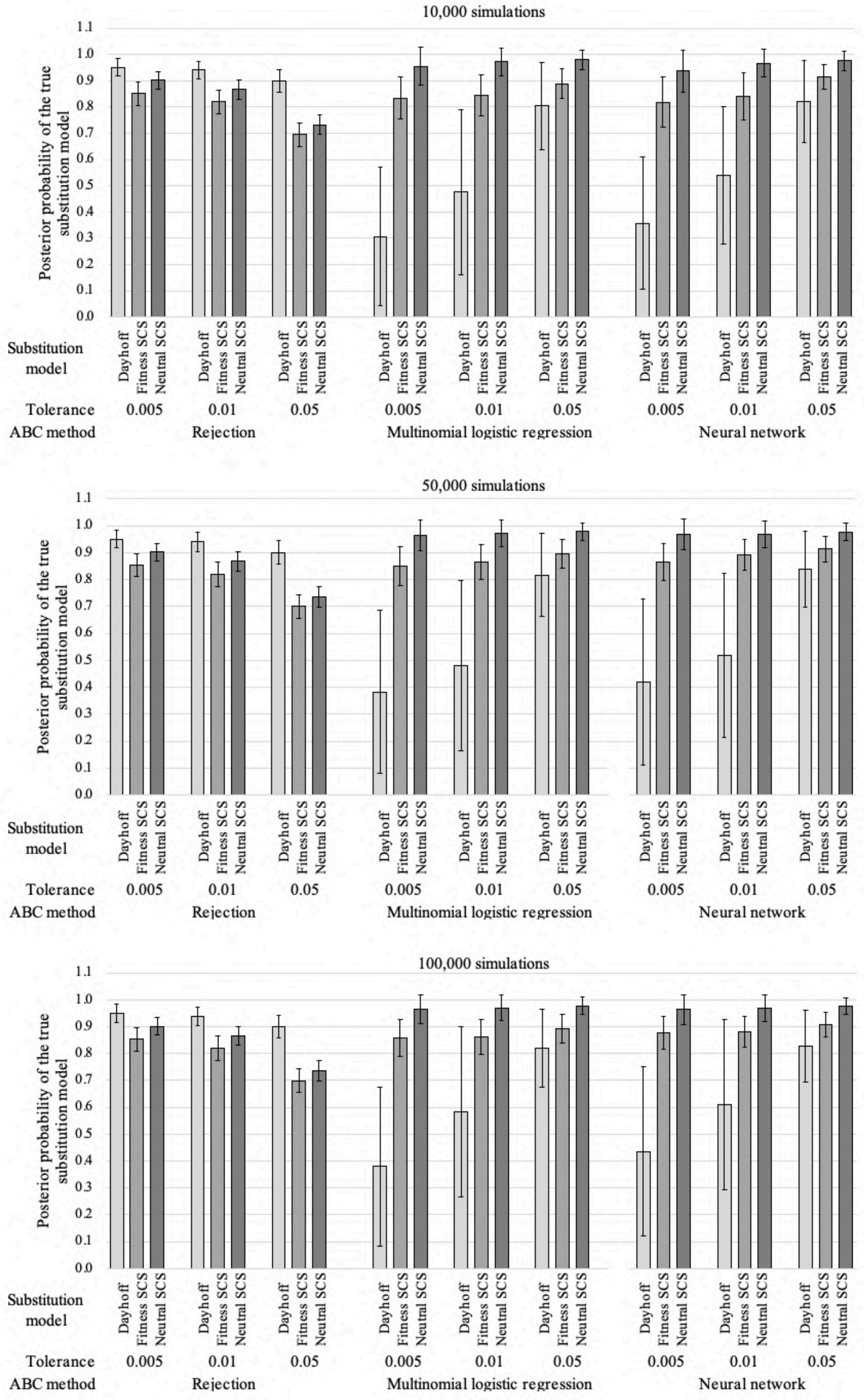

Figure S4. Goodness of fit for substitution model selection in the real dataset. Goodness of fit for the real datasets (Table 1, A-J) that includes: (1) histogram of the summary statistics from simulated data and summary statistics from the real data (blue vertical line) for every substitution model, (2) principal component analysis illustrating the goodness of fit of summary statistics from simulations under every substitution model with summary statistics from the real data (cross) and, (3) histograms of every summary statistic, including the protein folding stability mean (*DGREM_mean*) and standard deviation *(DGREM_sd)*, number of segregating sites (*SegSites)* and Grantham distance mean *(Grantham_mean_Position),* standard deviation *(Grantham_sd_Position),* skewness (*Grantham_sk_Position)* and kurtosis *(Grantham_ku_Position)* from data simulated under every substitution model and including the corresponding summary statistic from the real data (blue vertical line).

A. Tumor necrosis factor monkeypox protein family

1. Histogram of summary statistics from simulated (under every evaluated substitution model) and real data (blue vertical line).

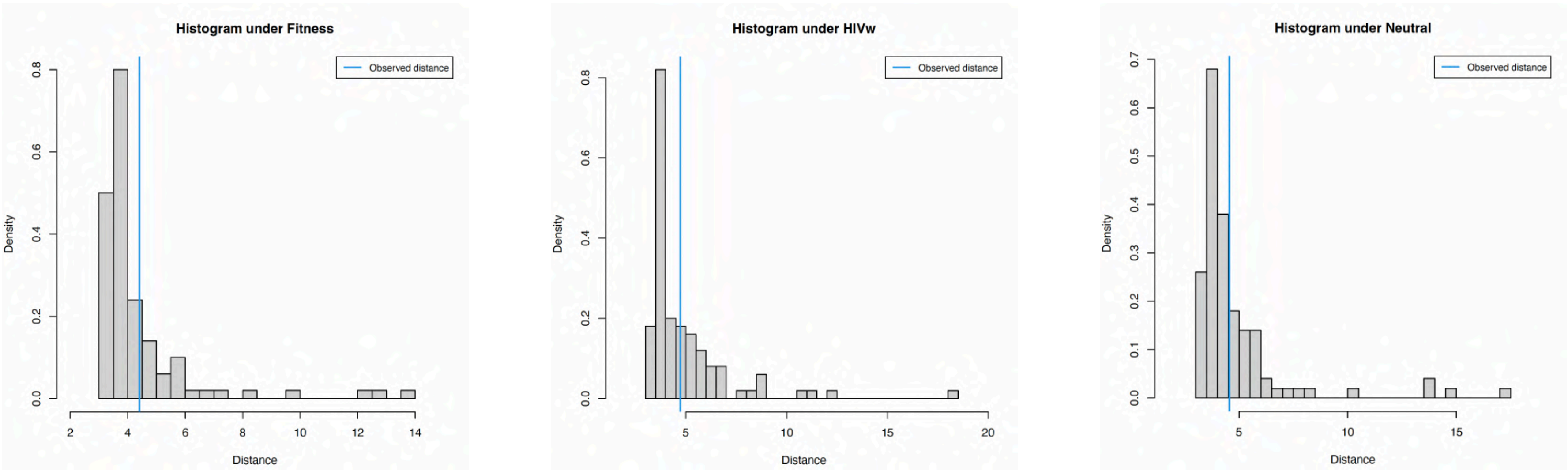

2. Principal component analysis of summary statistics from the data simulated under each substitution model and including summary statistics from the real data (cross).

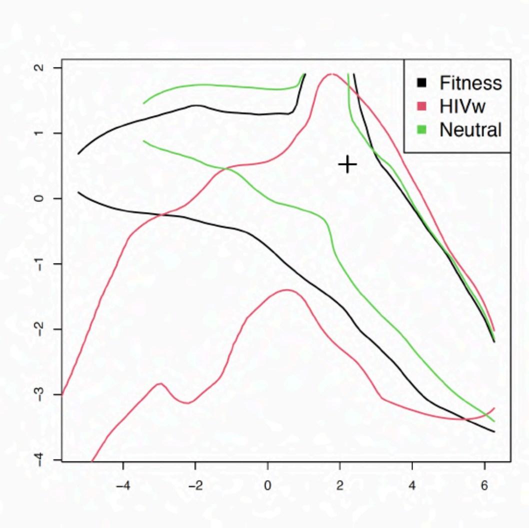

3. Histograms of protein folding stability mean (DGREM_mean) and standard deviation (DGREM_sd), number of segregating sites (SegSites) and Grantham distance mean (Grantham_mean_Position), standard deviation (Grantham_sd_Position), skewness (Grantham_sk_Position) and kurtosis (Grantham_ku_Position), from data simulated under every substitution model and including the corresponding summary statistics from the real data (blue vertical line).

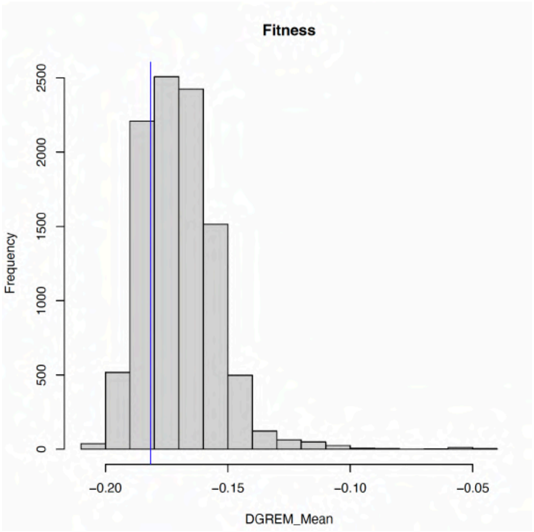

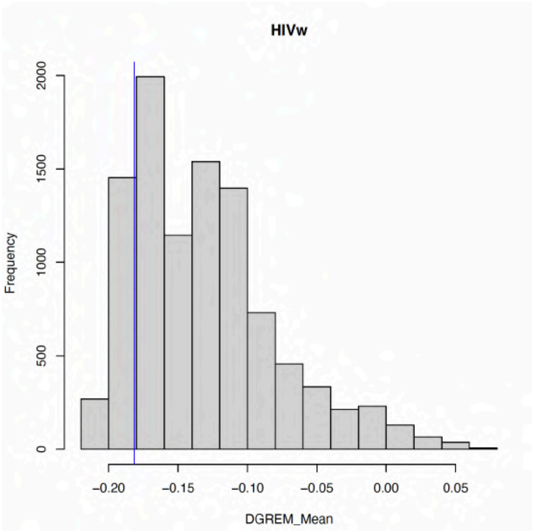

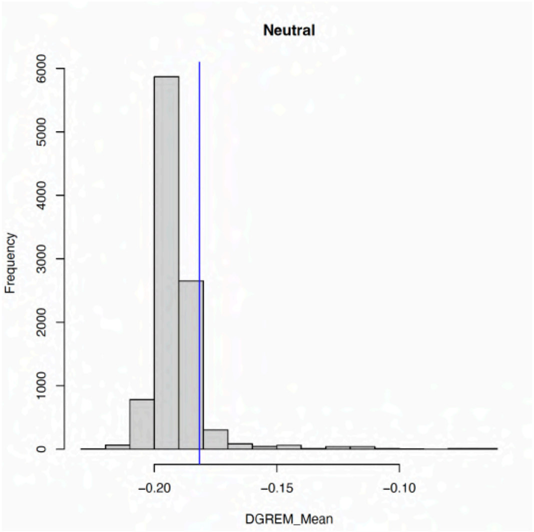

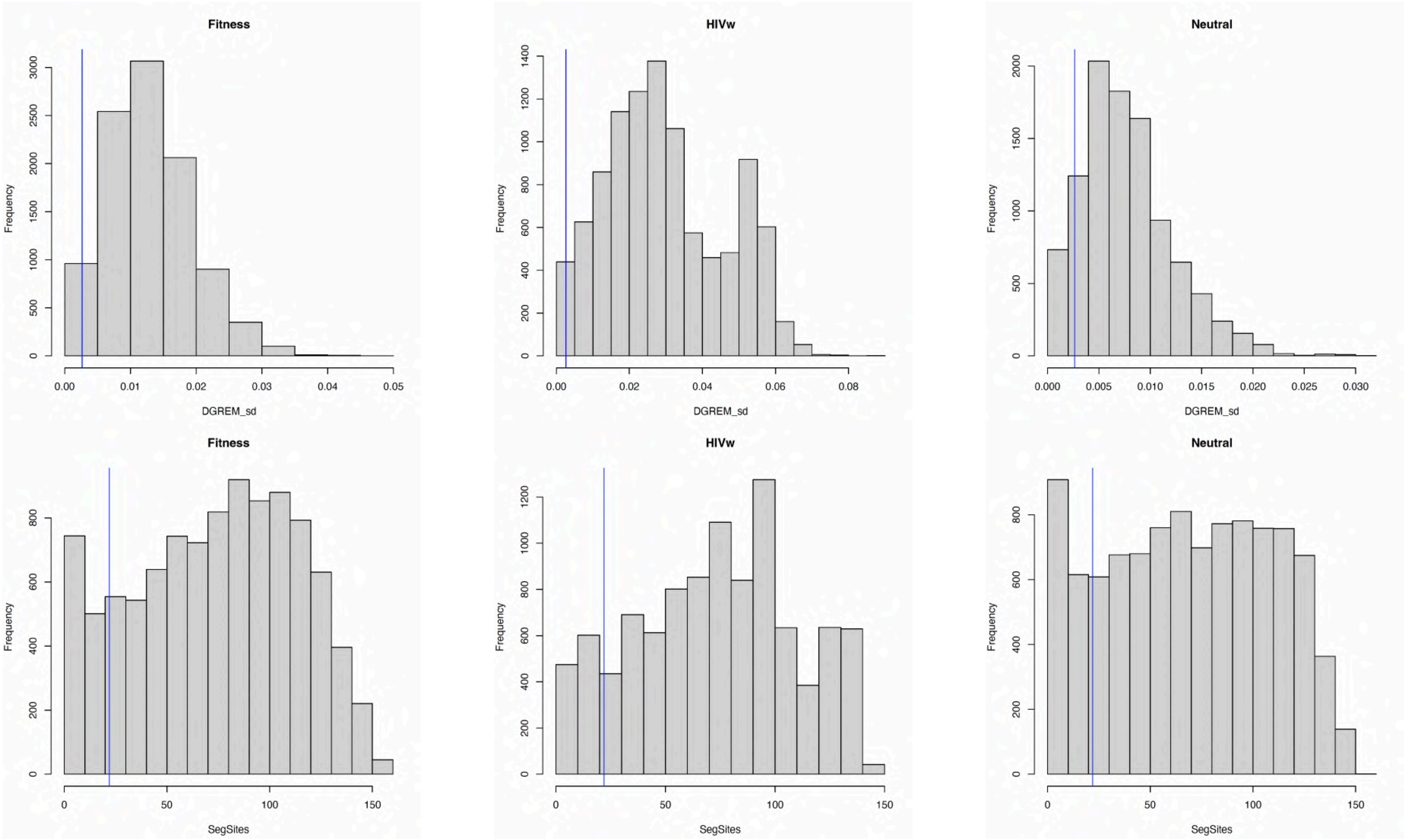
Fitness
HIVw
Neutral
Frequency
DGREM_sd
Fitness
HIVw
Neutral
SegSites

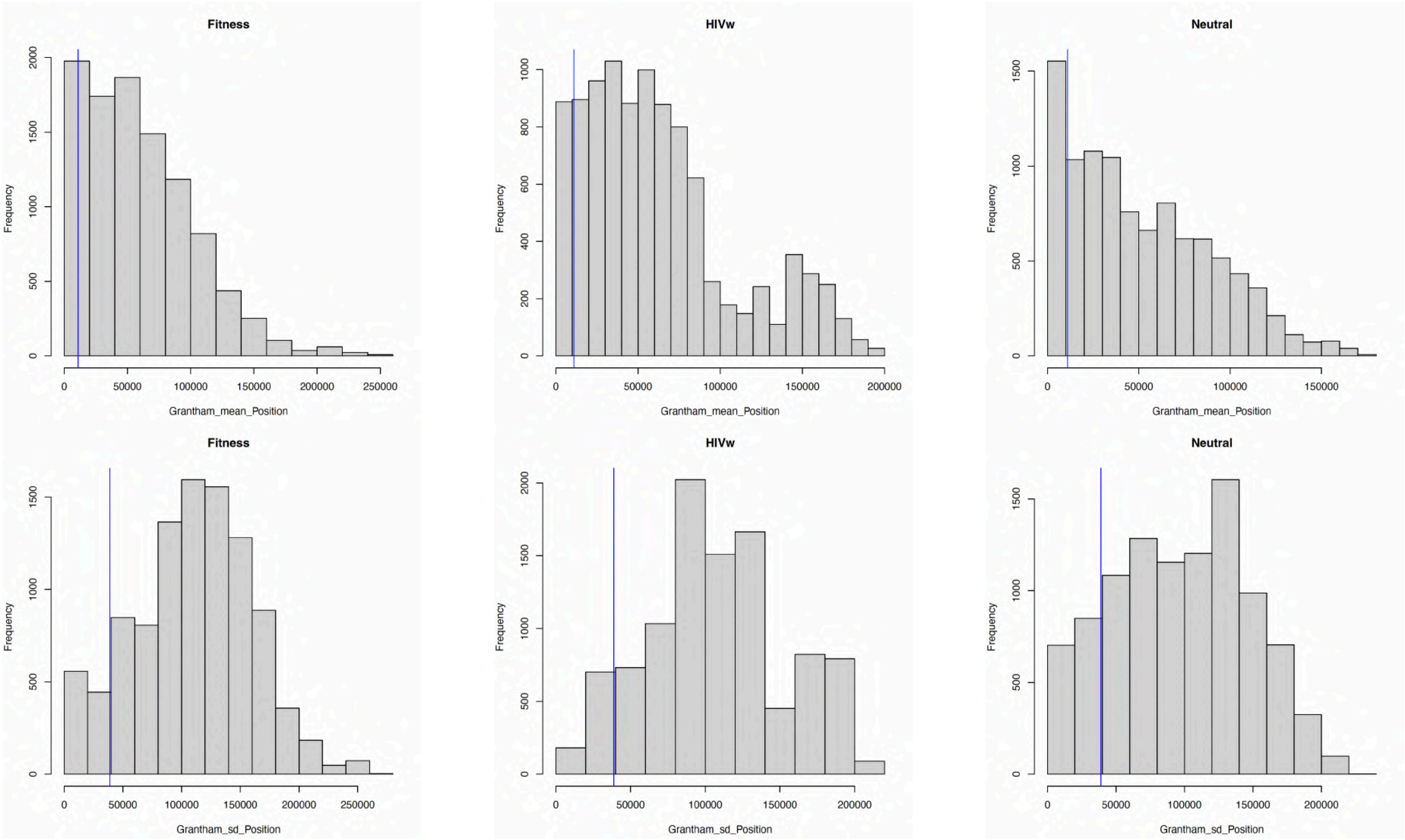
Fitness
HIVw
Neutral
Frequency
Grantham_mean_Position
Grantham_sd_Position

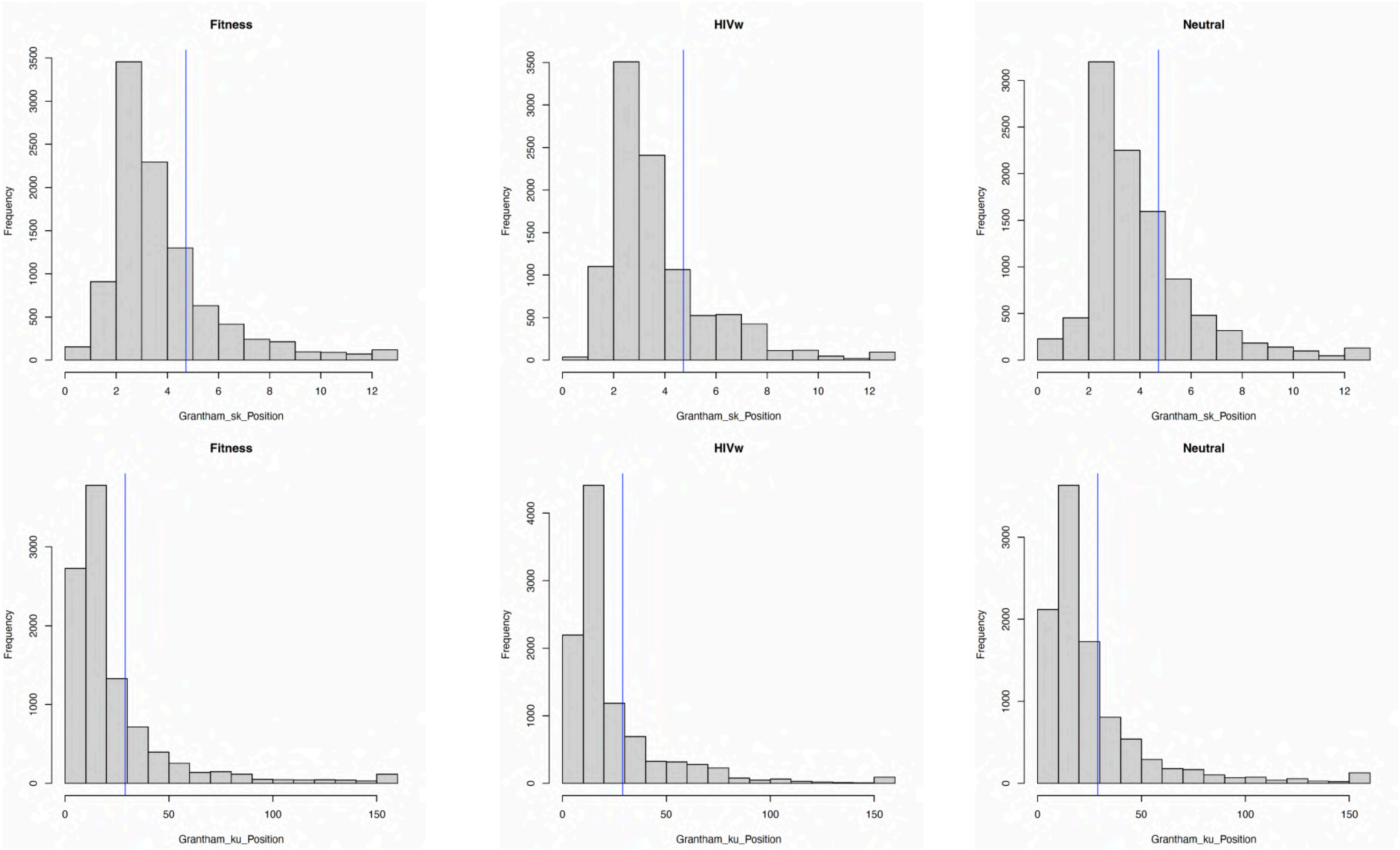
Fitness
HIVw
Neutral
Frequency
Grantham_sk_Position
Fitness
HIVw
Neutral
Frequency
Grantham_ku_Position

B. HIV protease protein family

1. Histogram of summary statistics from simulated (under every evaluated substitution model) and real data (blue vertical line).

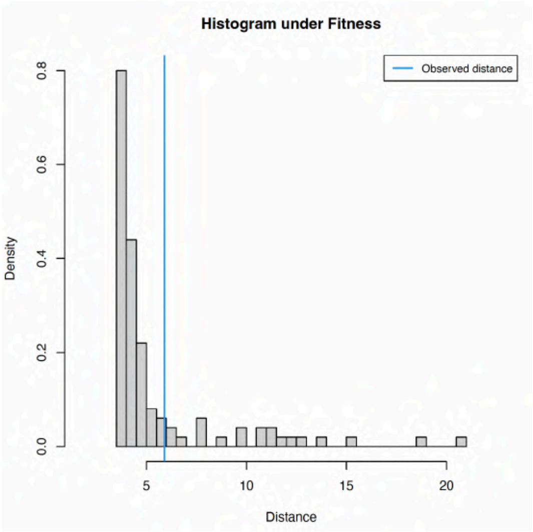

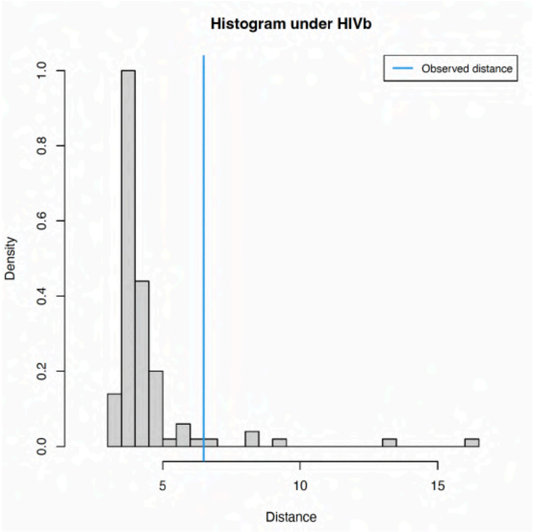

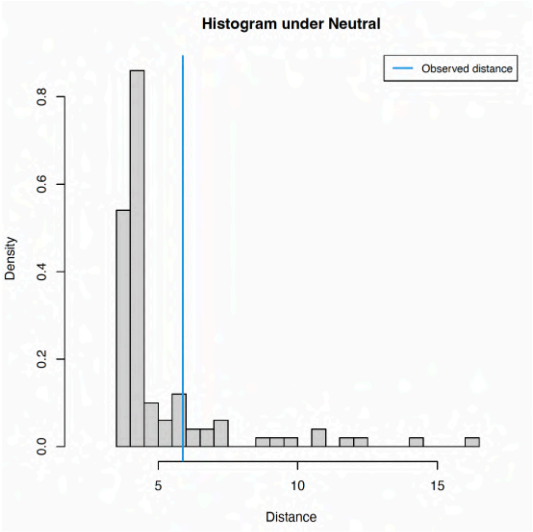

2. Principal component analysis of summary statistics from the data simulated under each substitution model and including summary statistics from the real data (cross).

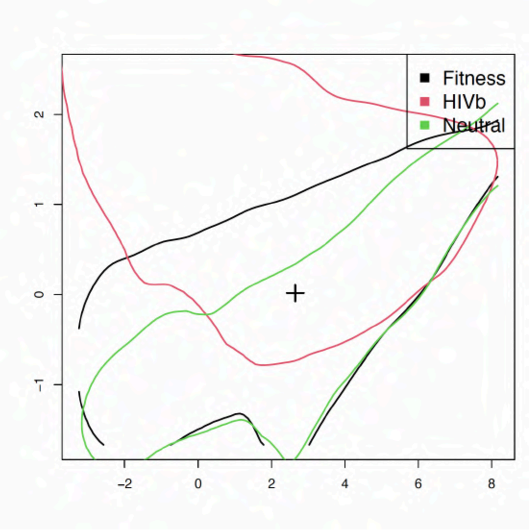

3. Histograms of protein folding stability mean (DGREM_mean) and standard deviation (DGREM_sd), number of segregating sites (SegSites) and Grantham distance mean (Grantham_mean_Position), standard deviation (Grantham_sd_Position), skewness (Grantham_sk_Position) and kurtosis (Grantham_ku_Position), from data simulated under every substitution model and including the corresponding summary statistics from the real data (blue vertical line).

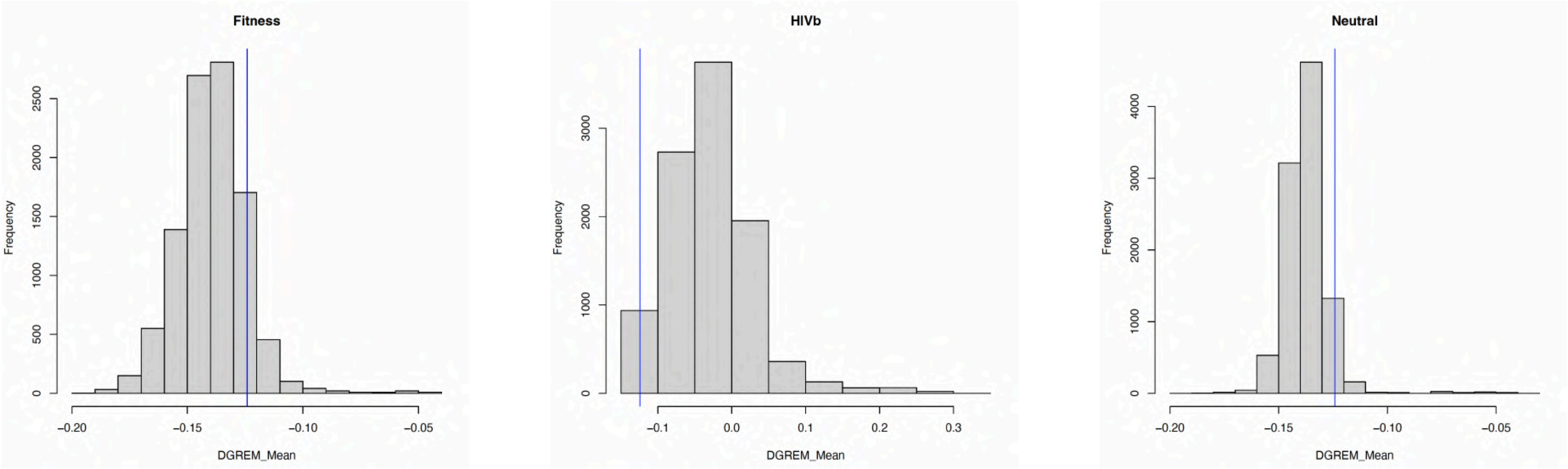

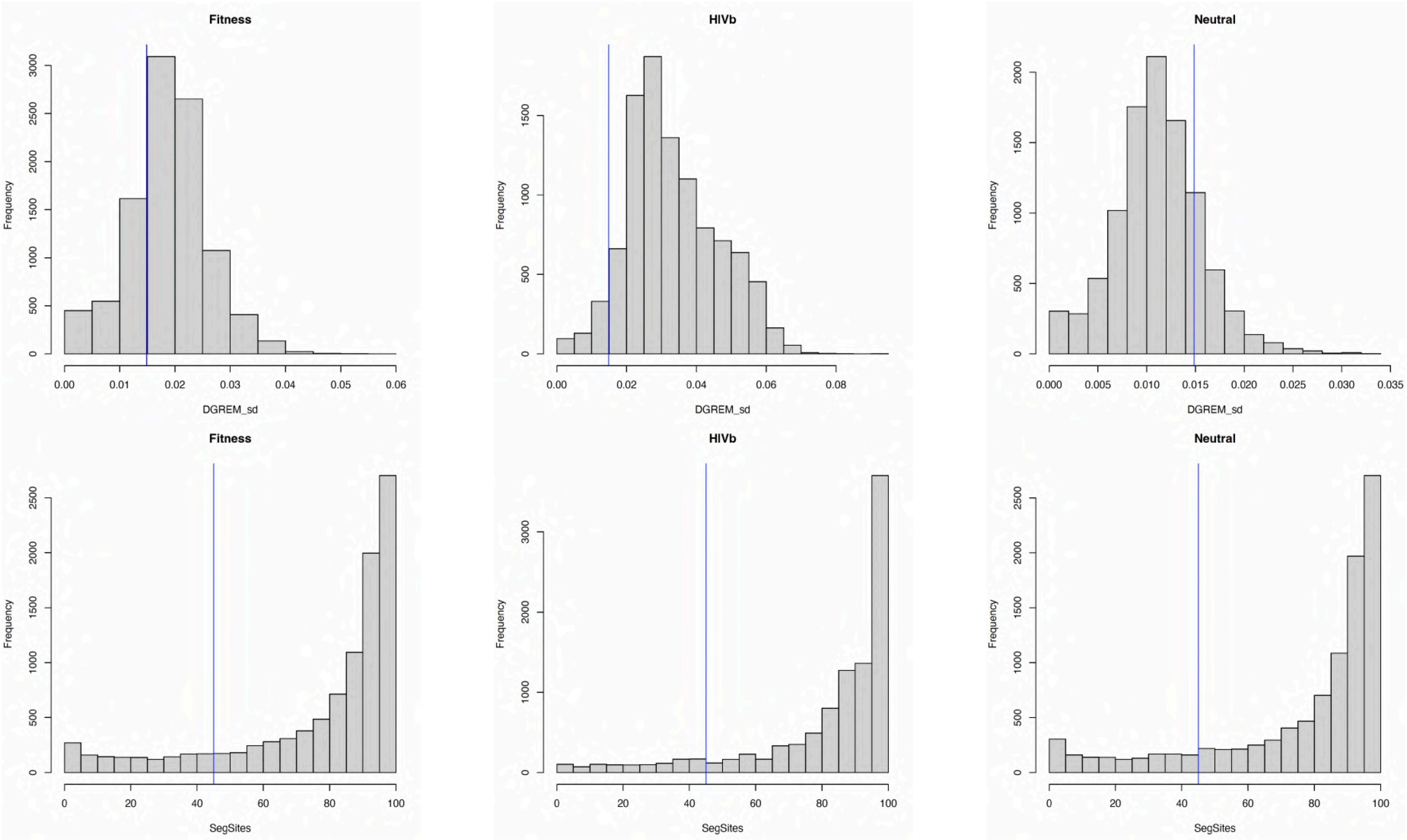
Fitness
HIVb
Neutral
Frequency
0
500
1000
1500
2000
2500
3000
0.00
0.01
0.02
0.03
0.04
0.05
0.06
0.08
0.000
0.005
0.010
0.015
0.020
0.025
0.030
0.035
DGREM_sd
Fitness
HIVb
Neutral
Frequency
0
20
40
60
80
100
SegSites

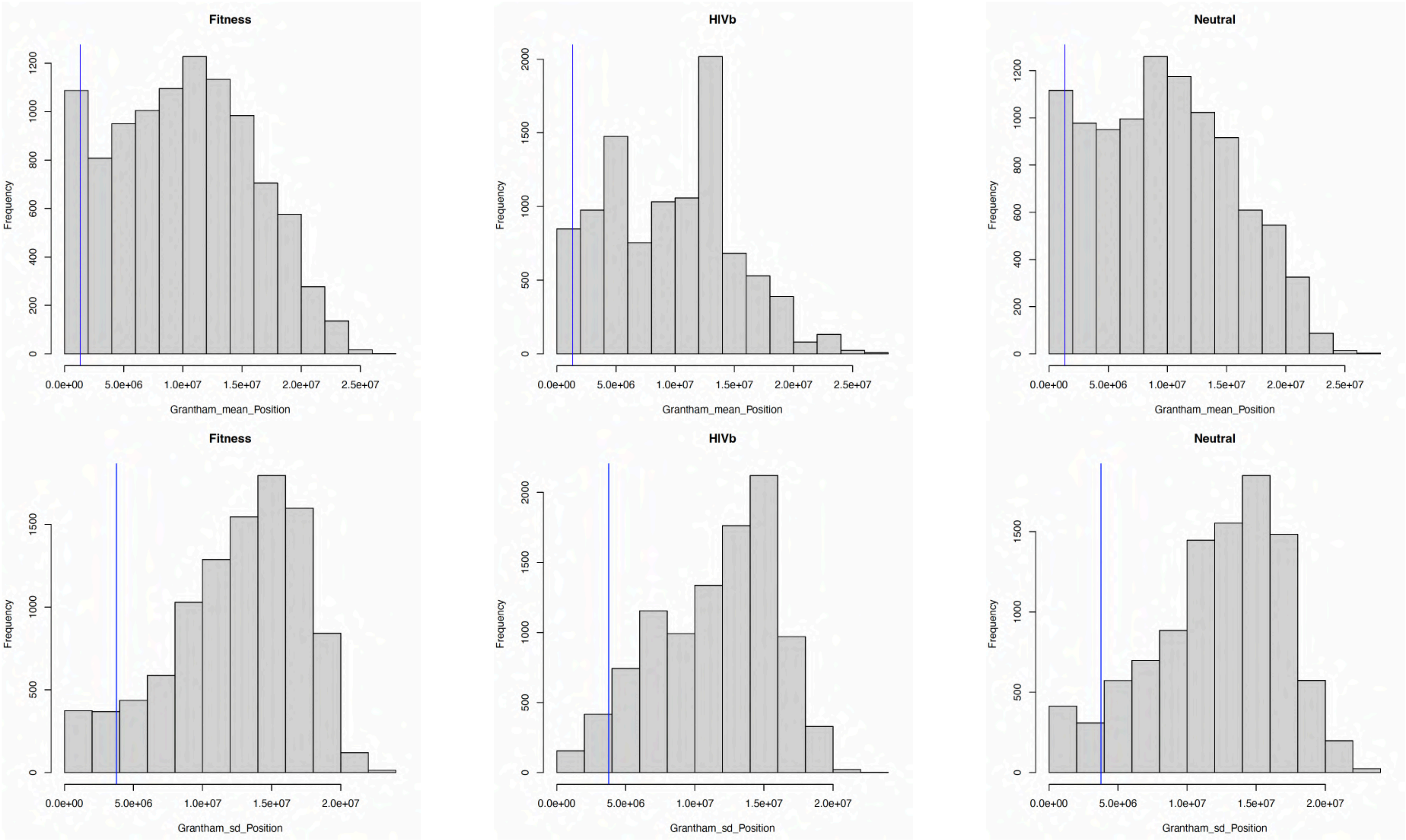
Fitness
HIVb
Neutral
Frequency
Grantham_mean_Position
Grantham_sd_Position
0.0e+00
5.0e+06
1.0e+07
1.5e+07
2.0e+07
2.5e+07

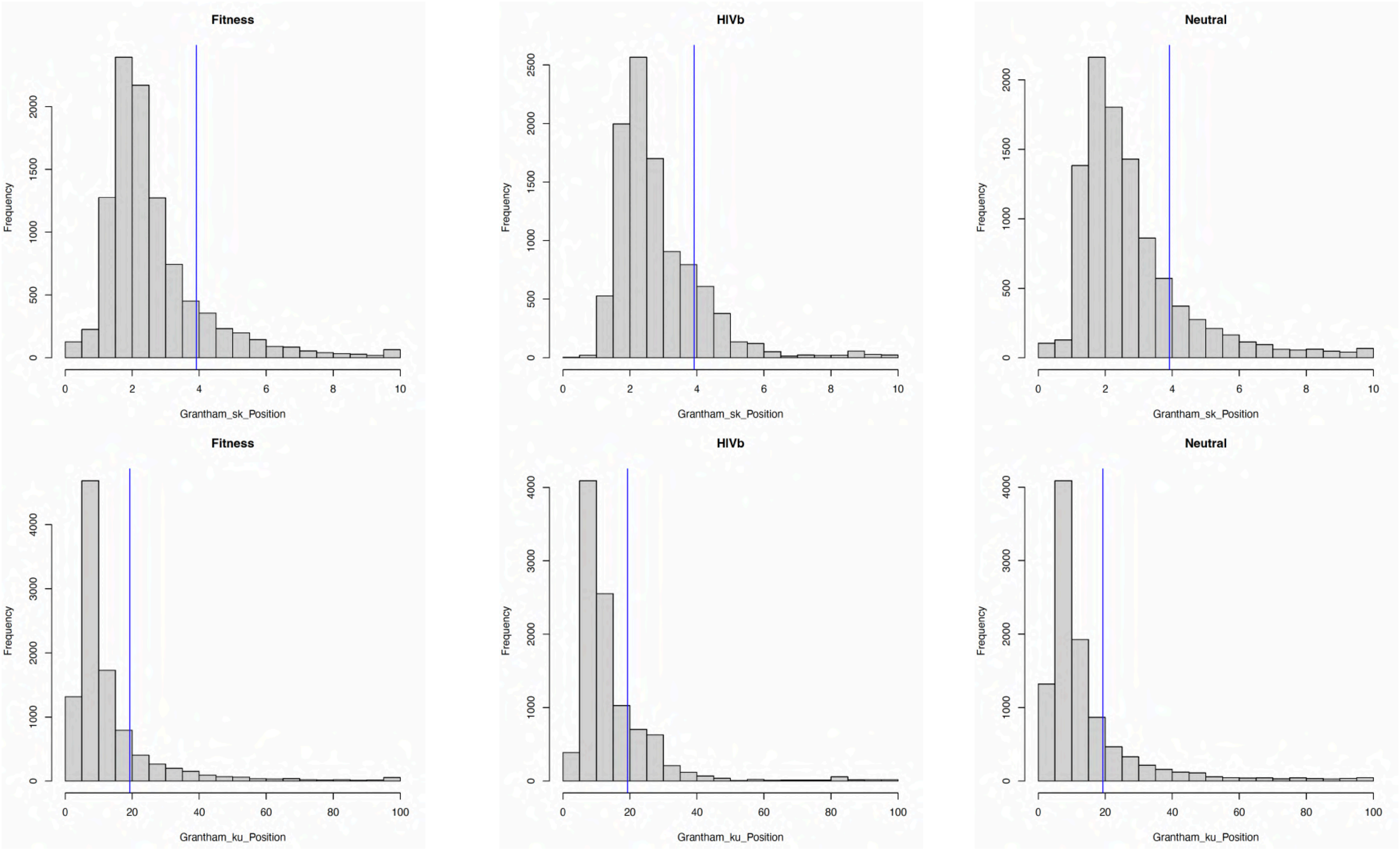
Fitness
HIVb
Neutral
Frequency
Grantham_sk_Position
Fitness
HIVb
Neutral
Frequency
Grantham_ku_Position

C. HIV gag polyprotein protein family

1. Histogram of summary statistics from simulated (under every evaluated substitution model) and real data (blue vertical line).

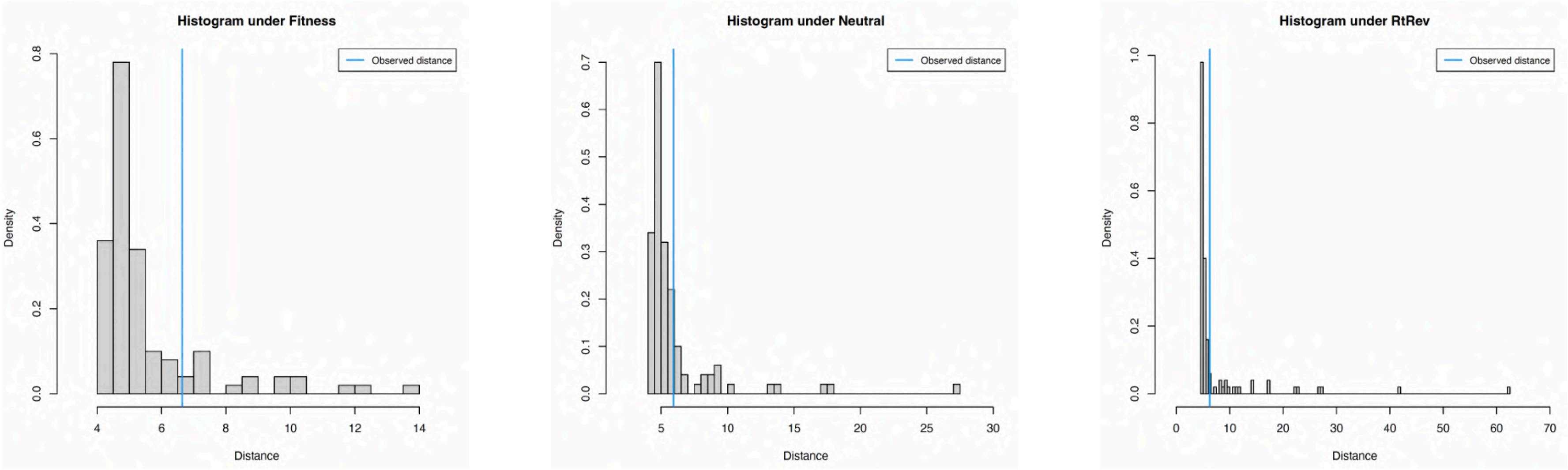

2. Principal component analysis of summary statistics from the data simulated under each substitution model and including summary statistics from the real data (cross).

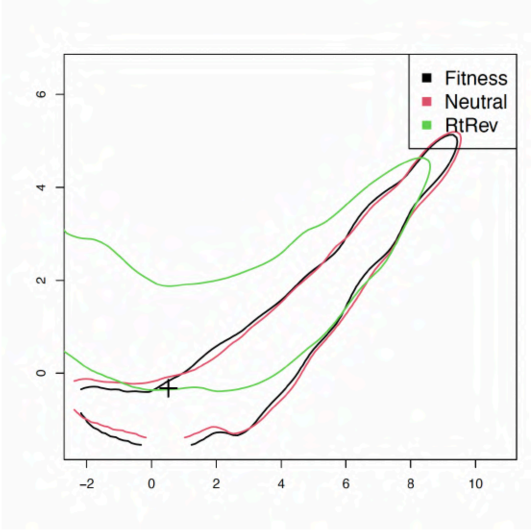

3. Histograms of protein folding stability mean (DGREM_mean) and standard deviation (DGREM_sd), number of segregating sites (SegSites) and Grantham distance mean (Grantham_mean_Position), standard deviation (Grantham_sd_Position), skewness (Grantham_sk_Position) and kurtosis (Grantham_ku_Position), from data simulated under every substitution model and including the corresponding summary statistics from the real data (blue vertical line).

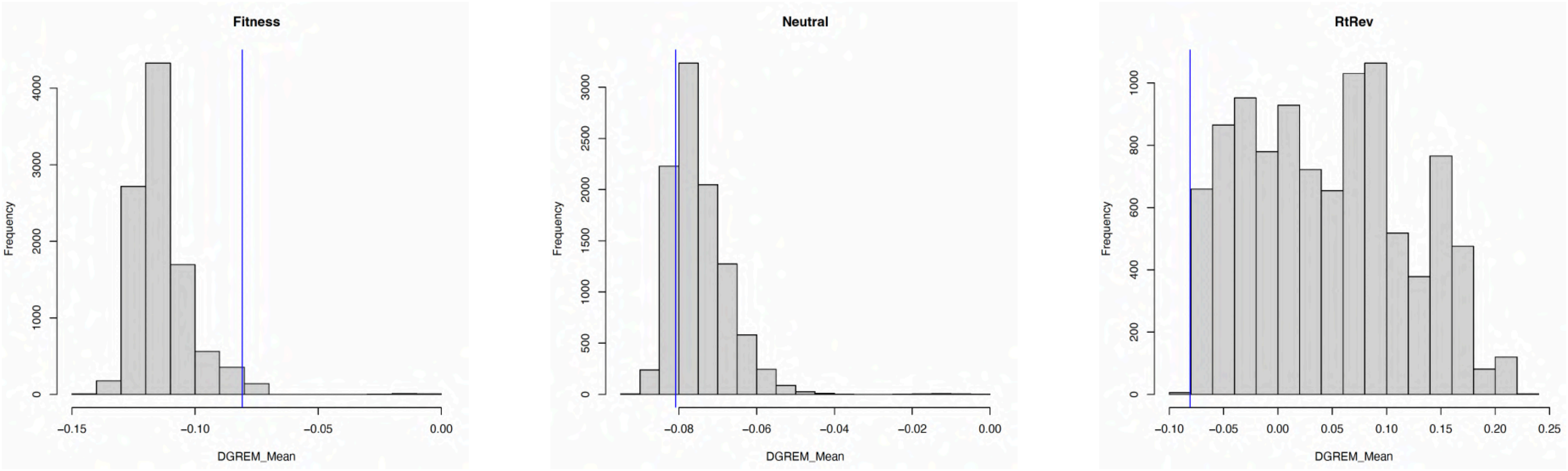

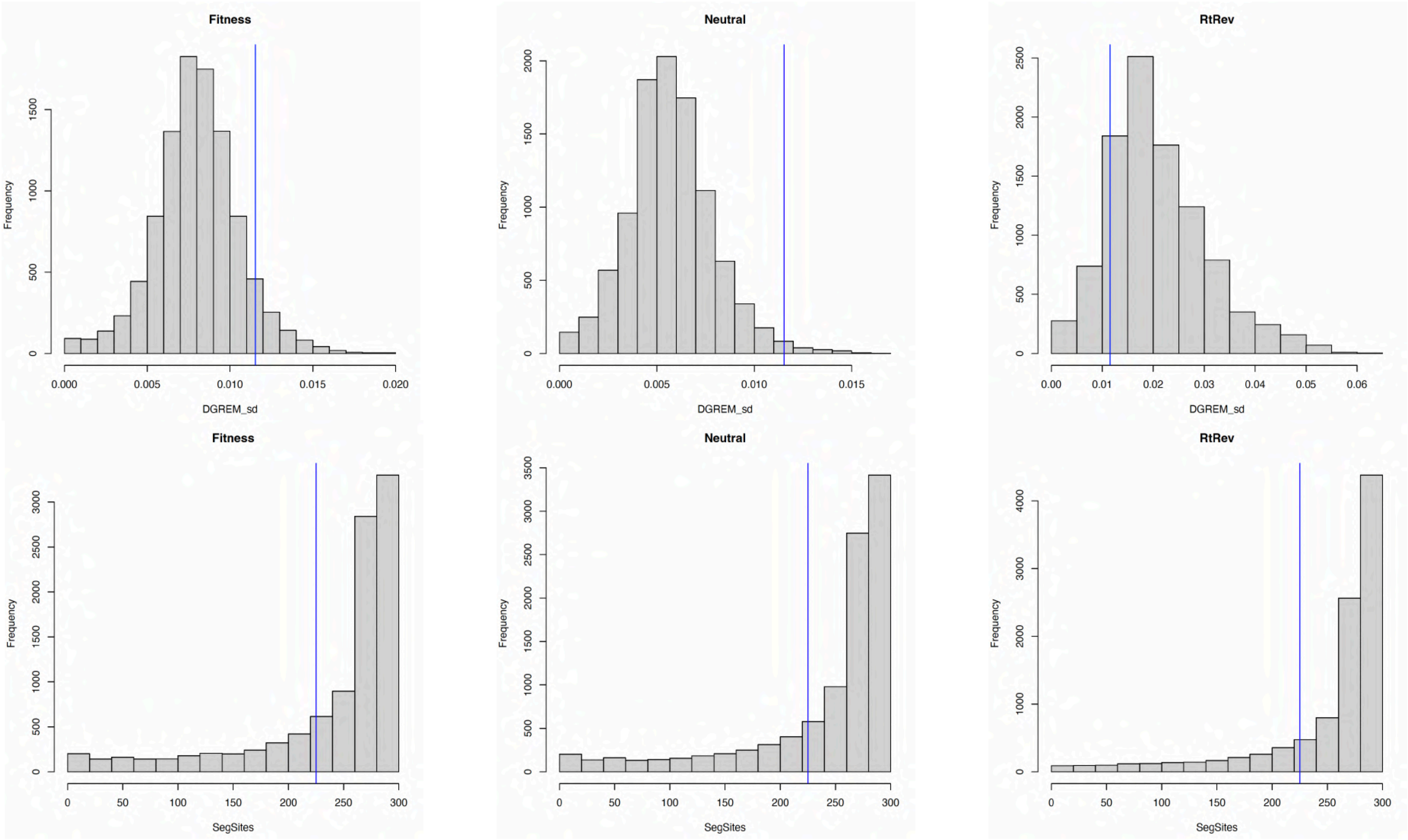
Fitness
Neutral
RtRev
Frequency
DGREM_sd
SegSites
0.000
0.005
0.010
0.015
0.020
0.00
0.01
0.02
0.03
0.04
0.05
0.06
0
50
100
150
200
250
300
500
1000
1500
2000
2500
3000
3500
4000

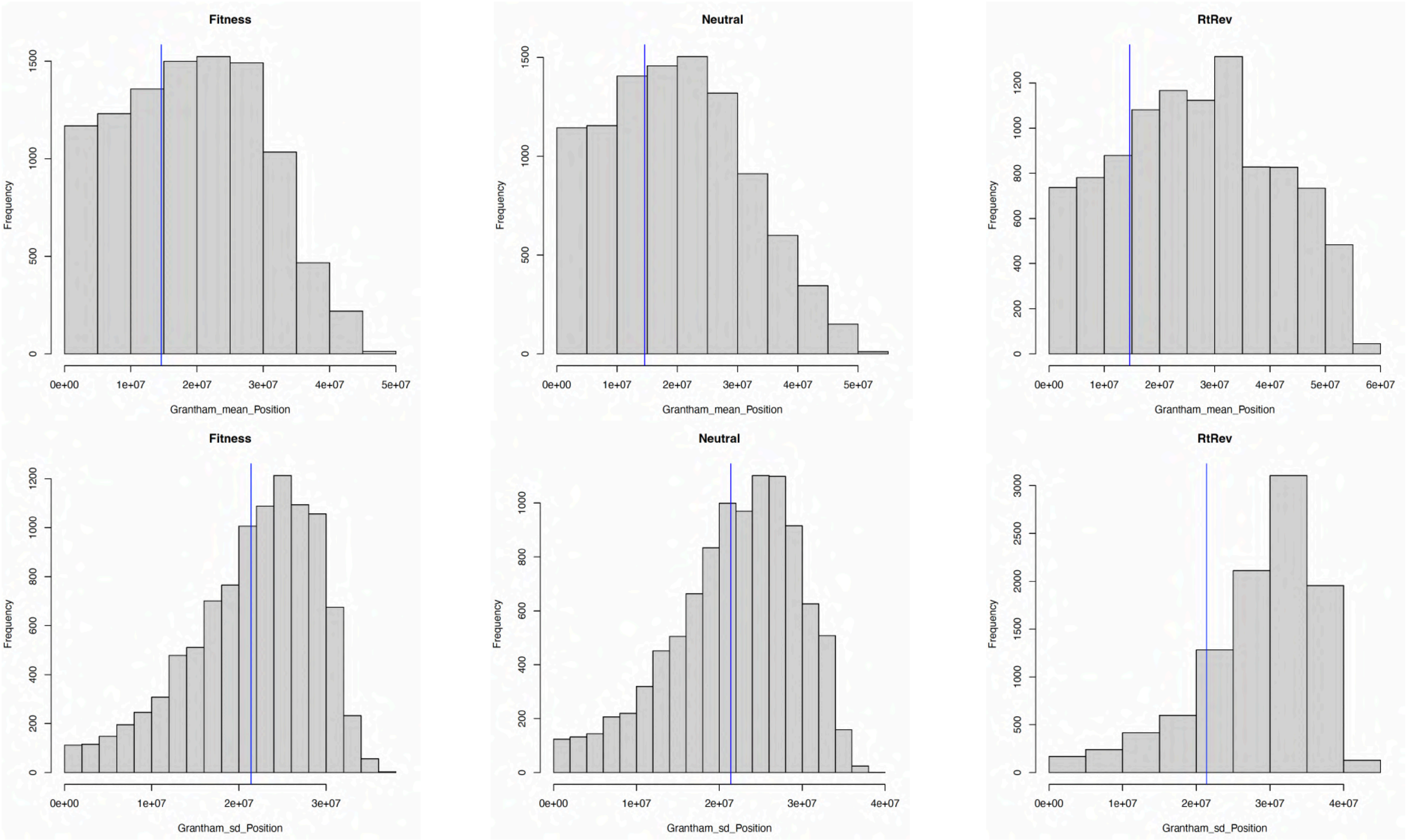
Fitness
Neutral
RtRev
Frequency
0
500
1000
1500
0e+00
1e+07
2e+07
3e+07
4e+07
5e+07
Grantham_mean_Position
0
500
1000
1500
0e+00
1e+07
2e+07
3e+07
4e+07
5e+07
Grantham_mean_Position
0
200
400
600
800
1000
1200
0e+00
1e+07
2e+07
3e+07
4e+07
5e+07
6e+07
Grantham_mean_Position
Fitness
Neutral
RtRev
0
200
400
600
800
1000
1200
0e+00
1e+07
2e+07
3e+07
Grantham_sd_Position
0
200
400
600
800
1000
0e+00
1e+07
2e+07
3e+07
4e+07
Grantham_sd_Position
0
500
1000
1500
2000
2500
3000
0e+00
1e+07
2e+07
3e+07
4e+07
Grantham_sd_Position

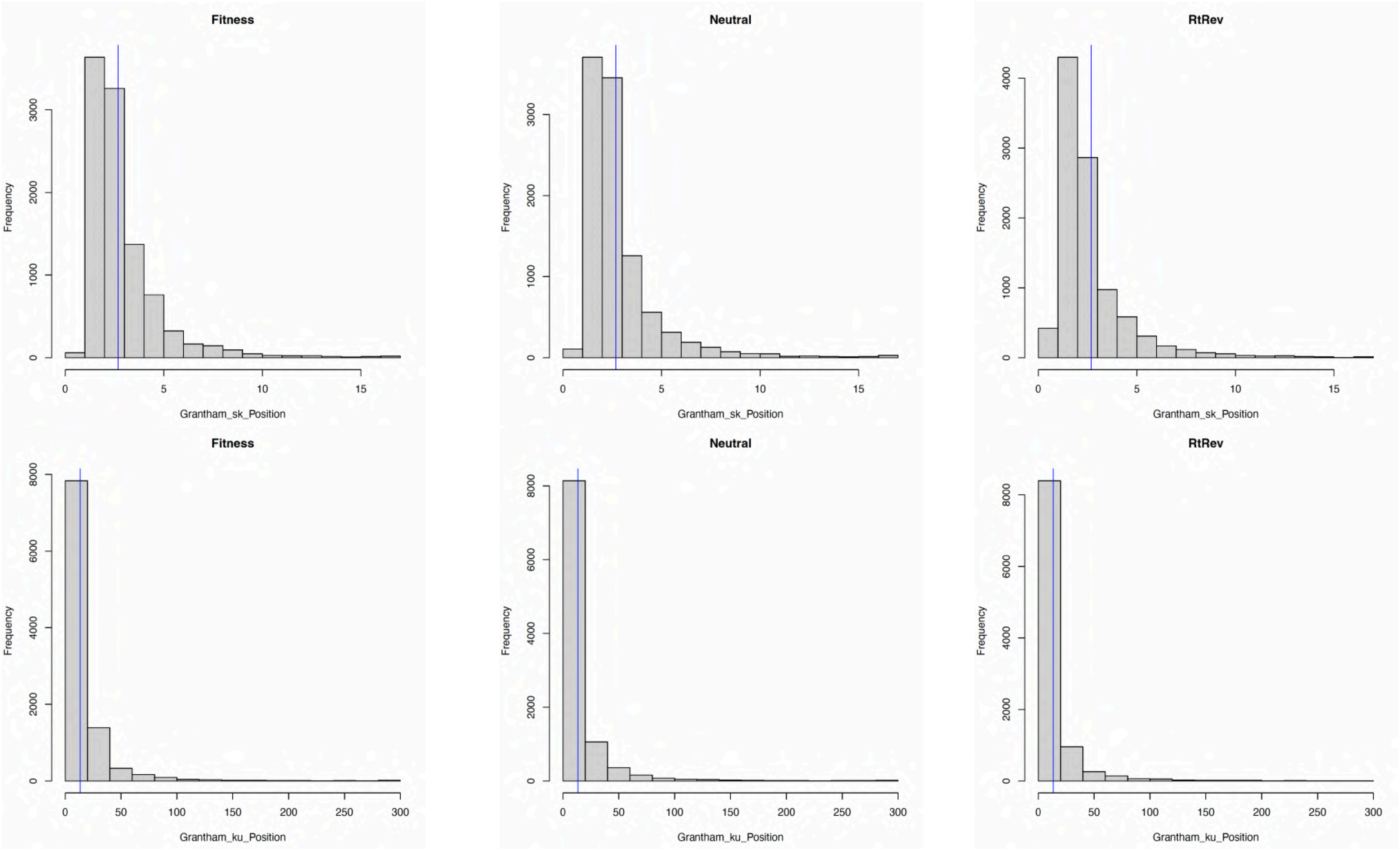
Fitness
Neutral
RtRev
Frequency
Grantham_sk_Position
Grantham_ku_Position
0
5
10
15
50
100
150
200
250
300
1000
2000
3000
4000
6000
8000

D. Influenza NS1

1. Histogram of summary statistics from simulated (under every evaluated substitution model) and real data (blue vertical line).

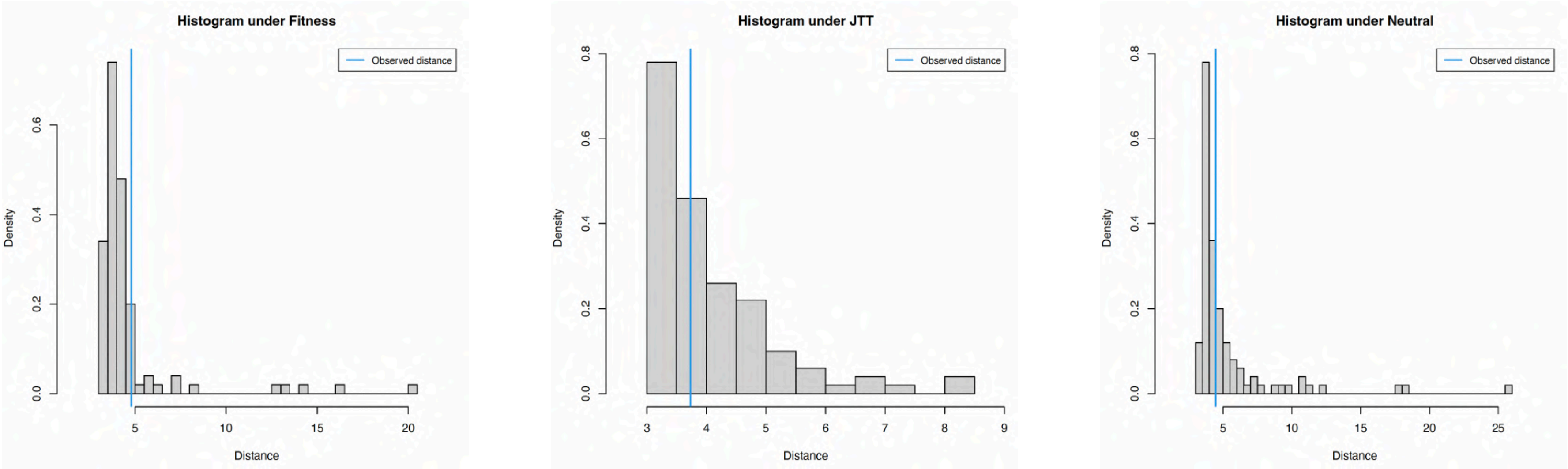

2. Principal component analysis of summary statistics from the data simulated under each substitution model and including summary statistics from the real data (cross).

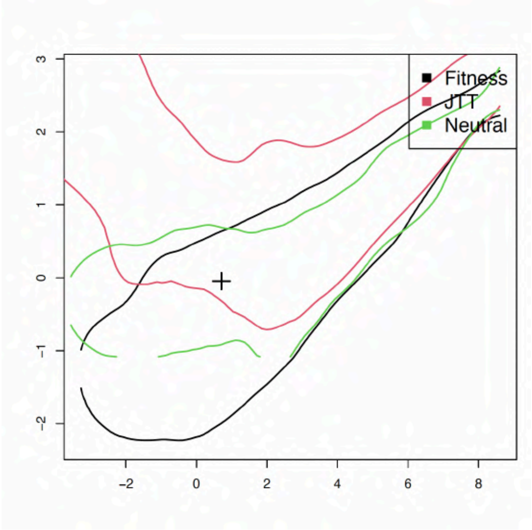

3. Histograms of protein folding stability mean (DGREM_mean) and standard deviation (DGREM_sd), number of segregating sites (SegSites) and Grantham distance mean (Grantham_mean_Position), standard deviation (Grantham_sd_Position), skewness (Grantham_sk_Position) and kurtosis (Grantham_ku_Position), from data simulated under every substitution model and including the corresponding summary statistics from the real data (blue vertical line).

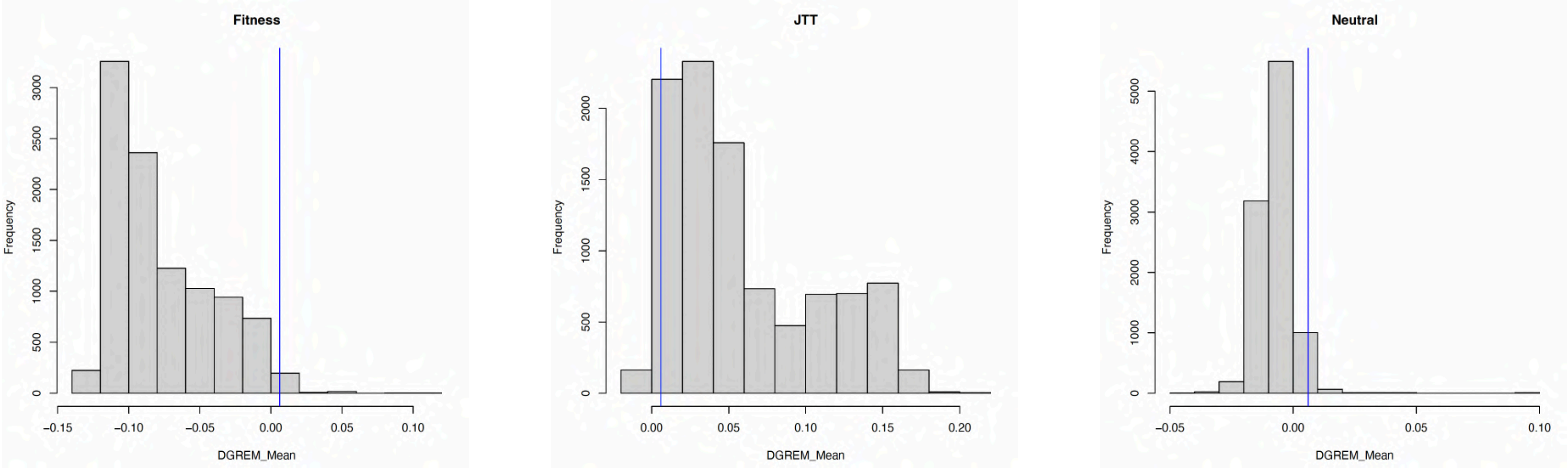

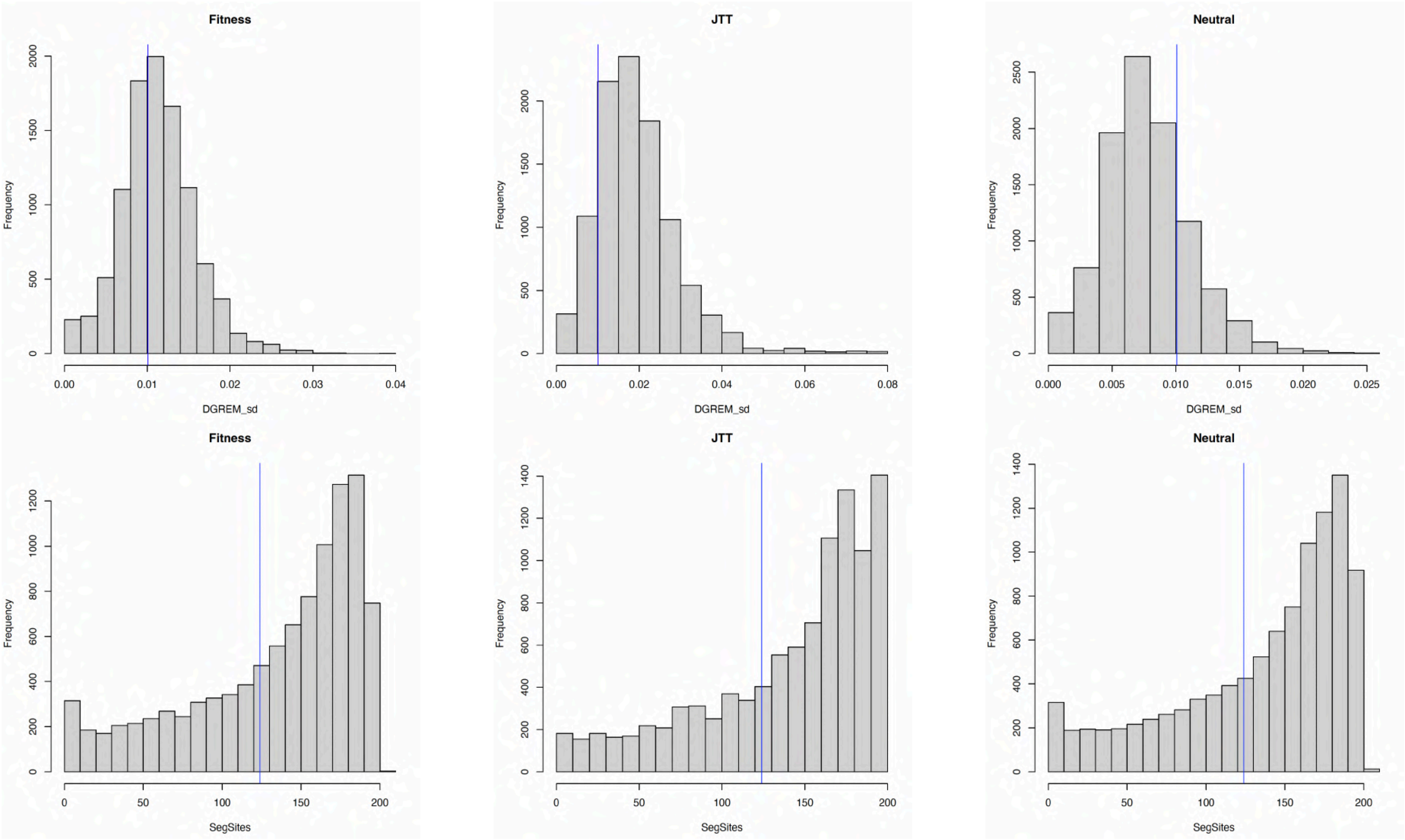
Fitness
JTT
Neutral
Frequency
DGREM_sd
Fitness
JTT
Neutral
Frequency
SegSites

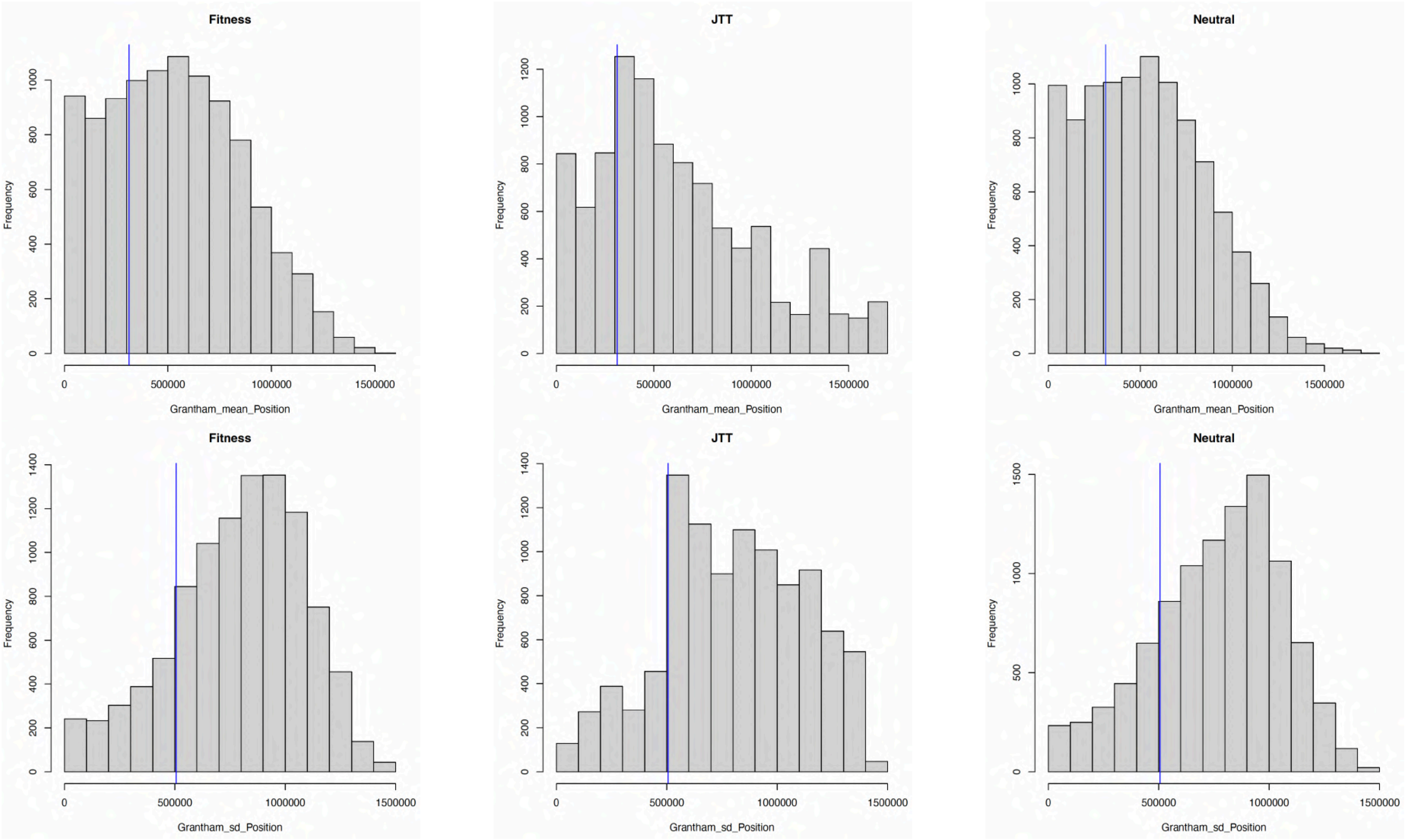

Fitness
JTT
Neutral
Frequency
Grantham_mean_Position
Grantham_sd_Position
0
500000
1000000
1500000

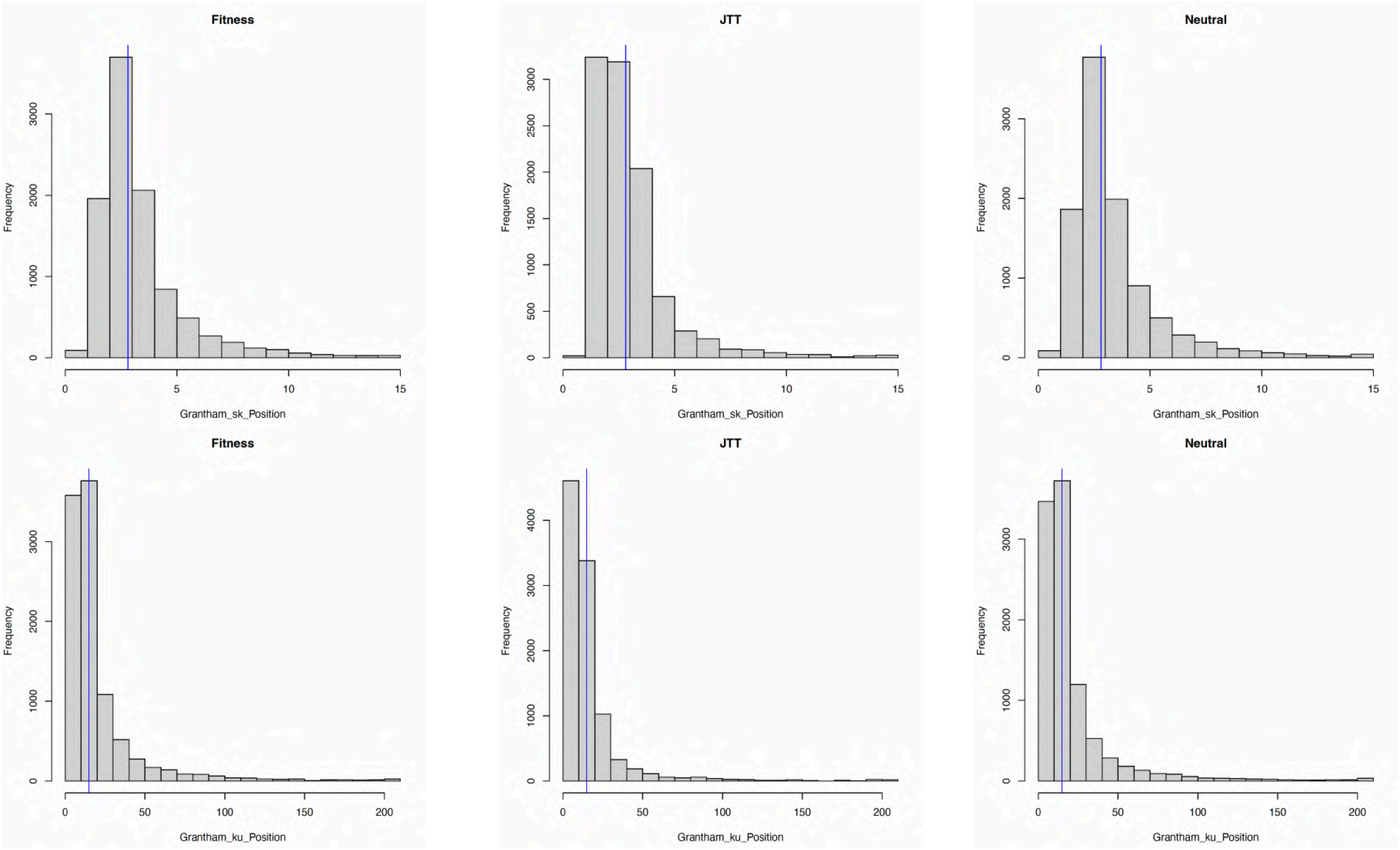
Fitness
JTT
Neutral
Frequency
Grantham_sk_Position
Fitness
JTT
Neutral
Frequency
Grantham_ku_Position

E. Coronavirus endopeptidase C30 protein family

1. Histogram of summary statistics from simulated (under every evaluated substitution model) and real data (blue vertical line).

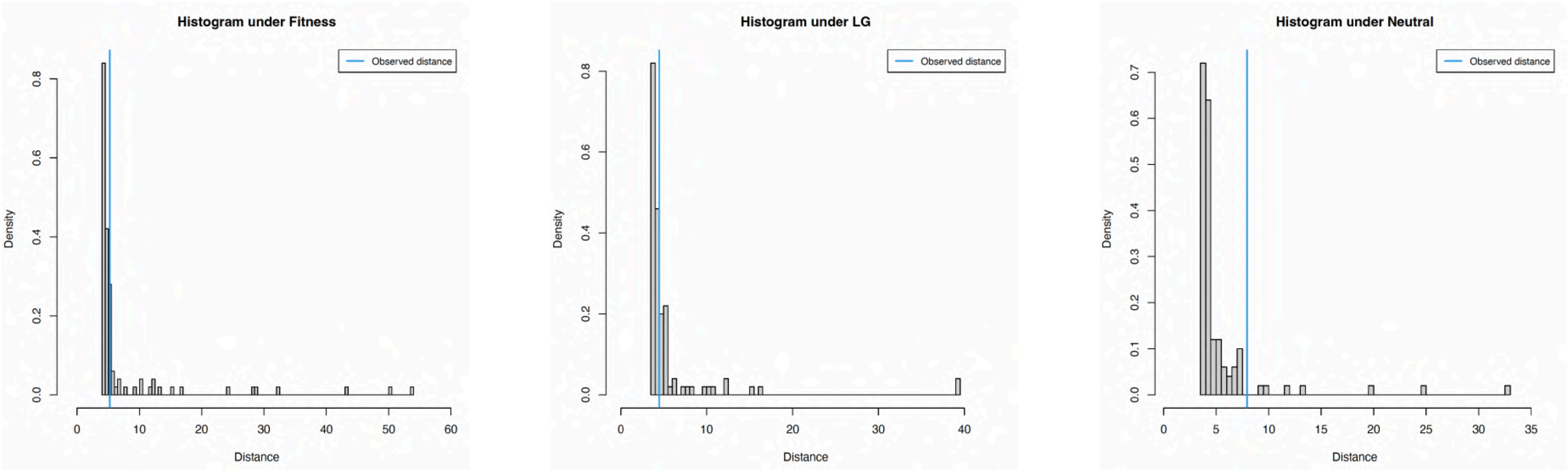

2. Principal component analysis of summary statistics from the data simulated under each substitution model and including summary statistics from the real data (cross).

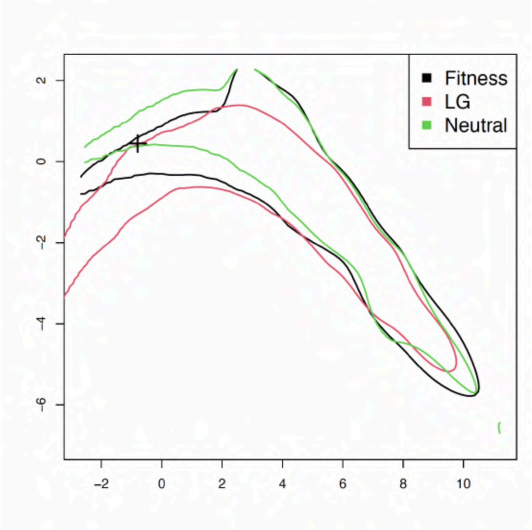

3. Histograms of protein folding stability mean (DGREM_mean), number of segregating sites (SegSites) and Grantham distance mean (Grantham_mean_Position), standard deviation (Grantham_sd_Position), skewness (Grantham_sk_Position) and kurtosis (Grantham_ku_Position), from data simulated under every substitution model and including the corresponding summary statistics from the real data (blue vertical line).

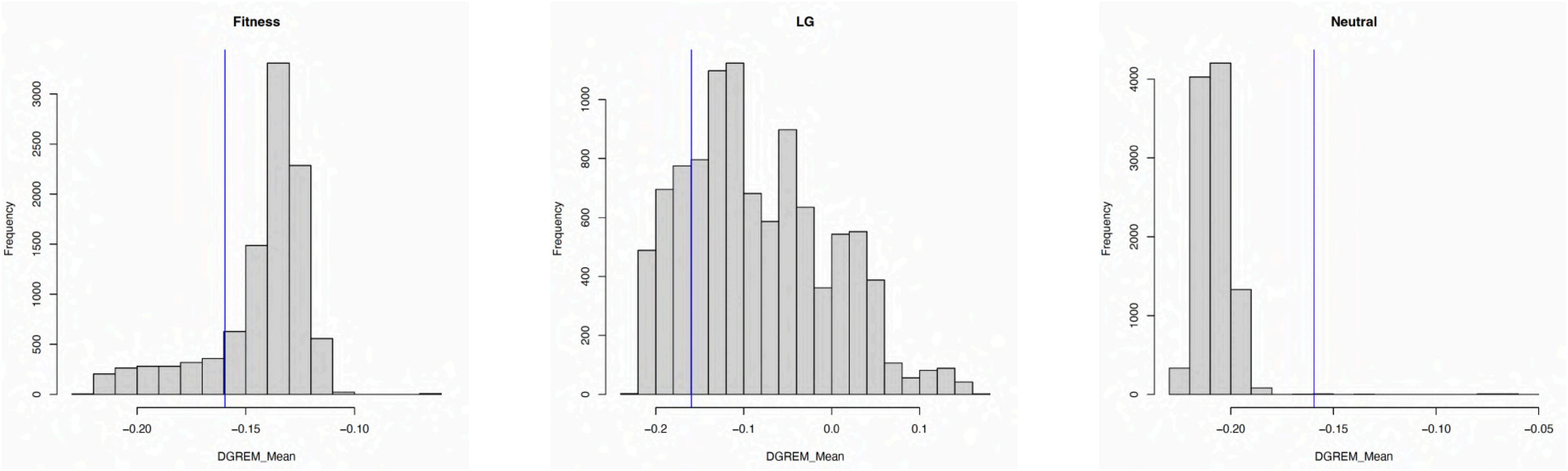

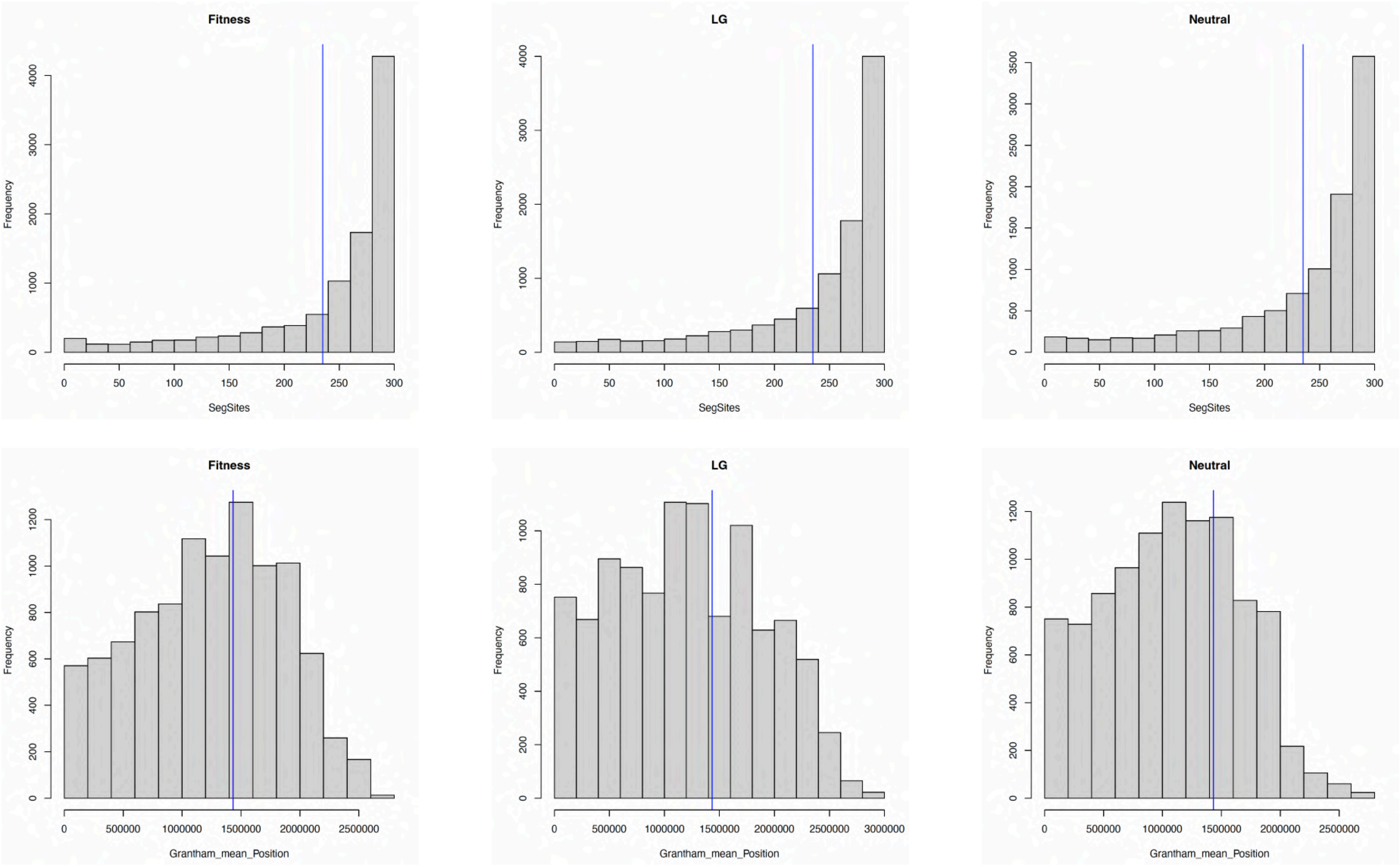
Fitness
Frequency
SegSites
LG
Frequency
SegSites
Neutral
Frequency
SegSites
Fitness
Frequency
Grantham_mean_Position
LG
Frequency
Grantham_mean_Position
Neutral
Frequency
Grantham_mean_Position

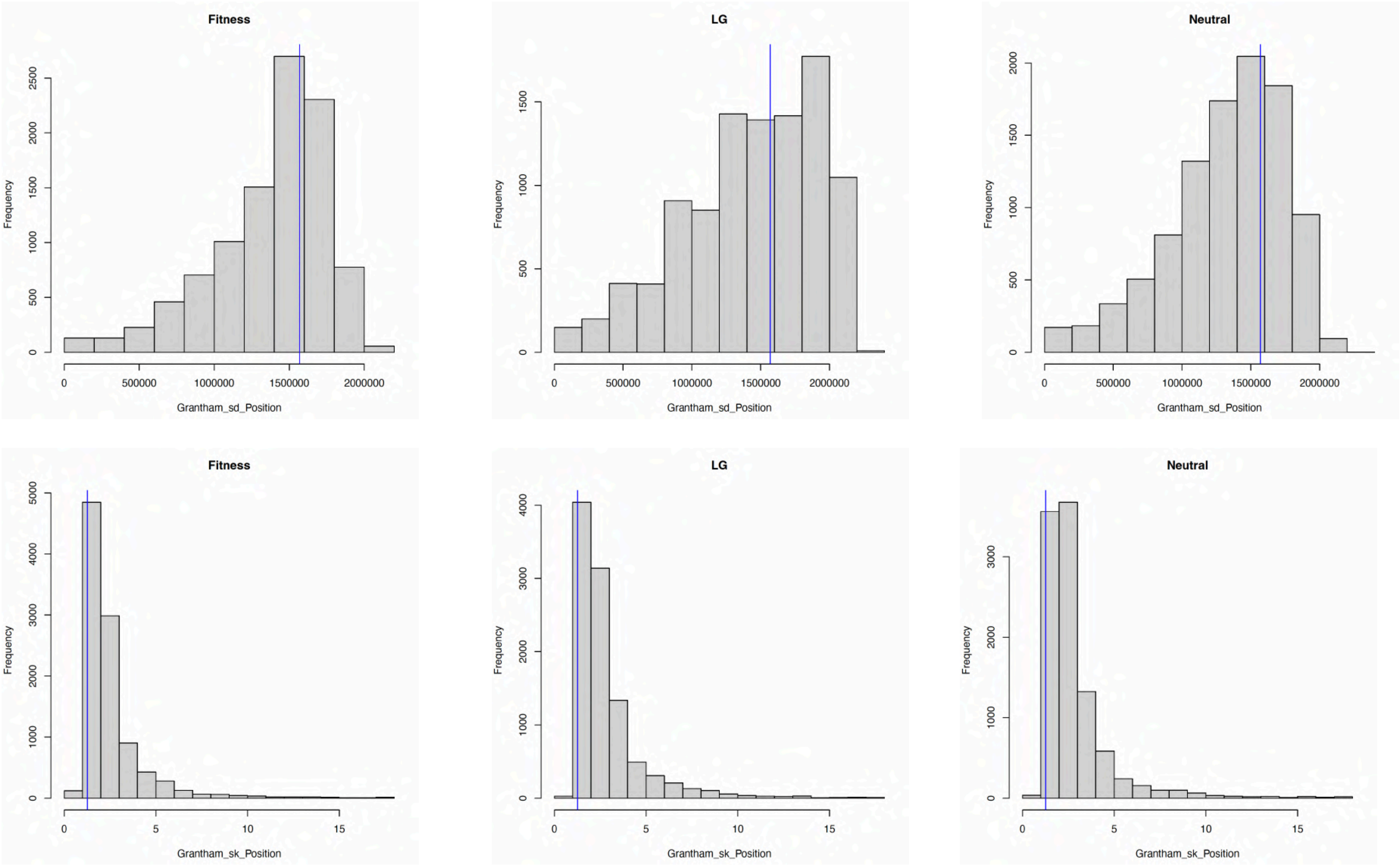

Fitness
Frequency
Grantham_sd_Position
0
500000
1000000
1500000
2000000
500
1000
1500
2000
2500
LG
Frequency
Grantham_sd_Position
Neutral
Frequency
Grantham_sd_Position
Fitness
Frequency
Grantham_sk_Position
5
10
15
LG
Frequency
Grantham_sk_Position
Neutral
Frequency
Grantham_sk_Position

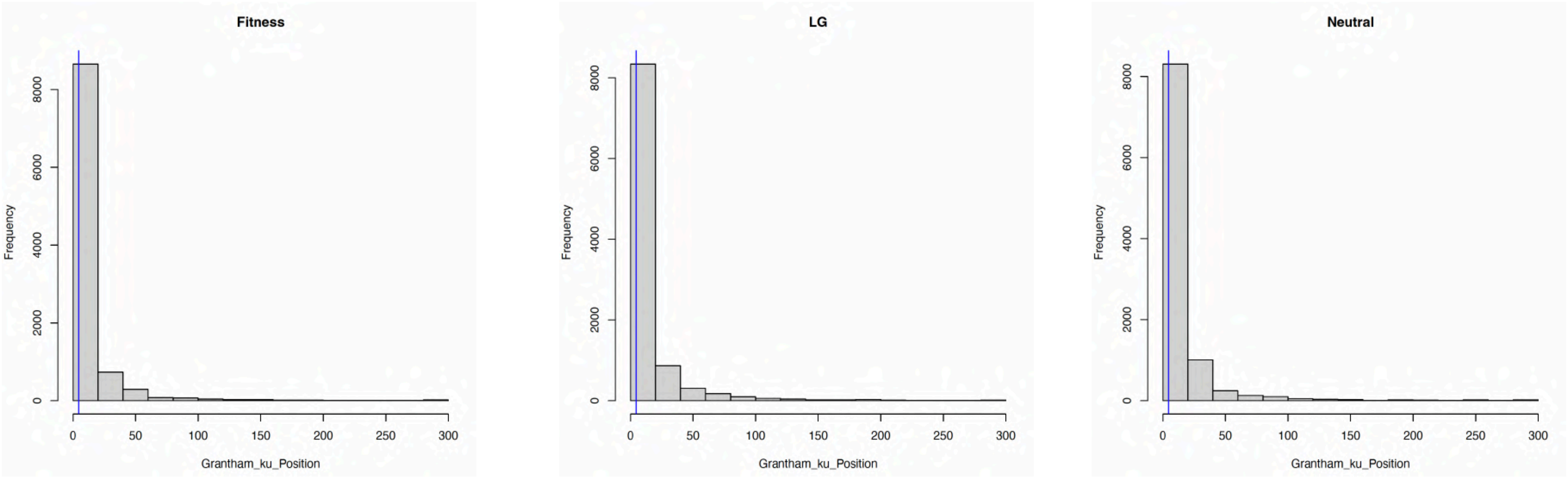
Fitness
LG
Neutral
Frequency
0
2000
4000
6000
8000
0
50
100
150
200
250
300
Grantham_ku_Position

F. Coronavirus 2'-O-methyltransferase protein family

1. Histogram of summary statistics from simulated (under every evaluated substitution model) and real data (blue vertical line).

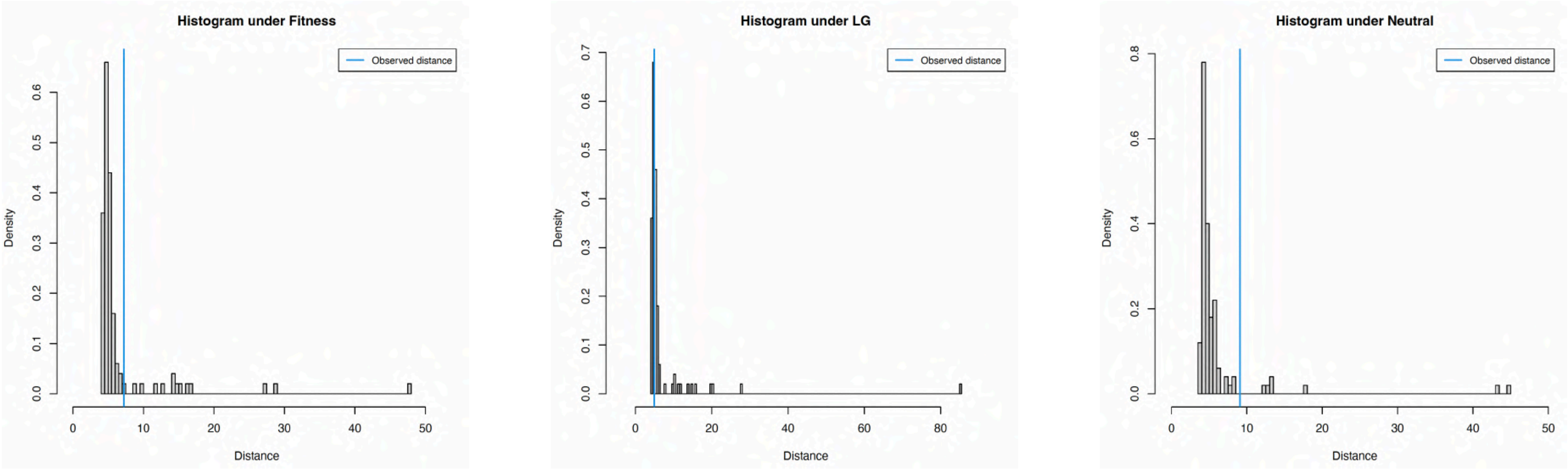

2. Principal component analysis of summary statistics from the data simulated under each substitution model and including summary statistics from the real data (cross).

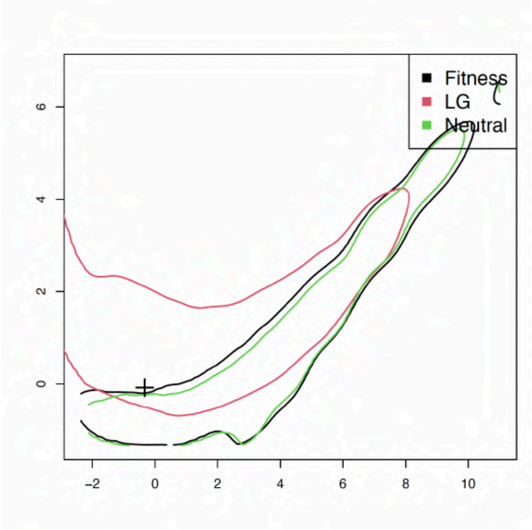

3. Histograms of protein folding stability mean (DGREM_mean) and standard deviation (DGREM_sd), number of segregating sites (SegSites) and Grantham distance mean (Grantham_mean_Position), standard deviation (Grantham_sd_Position), skewness (Grantham_sk_Position) and kurtosis (Grantham_ku_Position), from data simulated under every substitution model and including the corresponding summary statistics from the real data (blue vertical line).

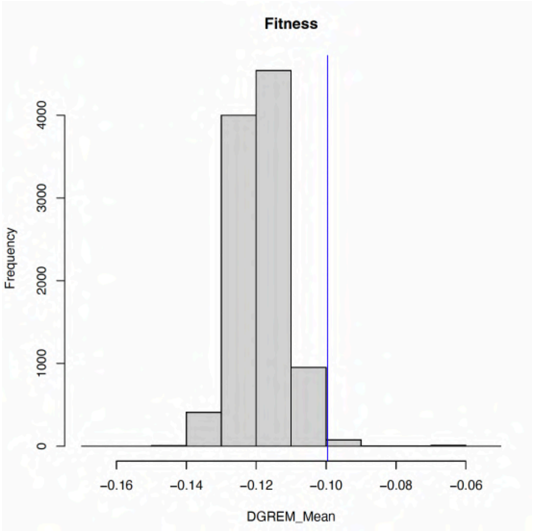

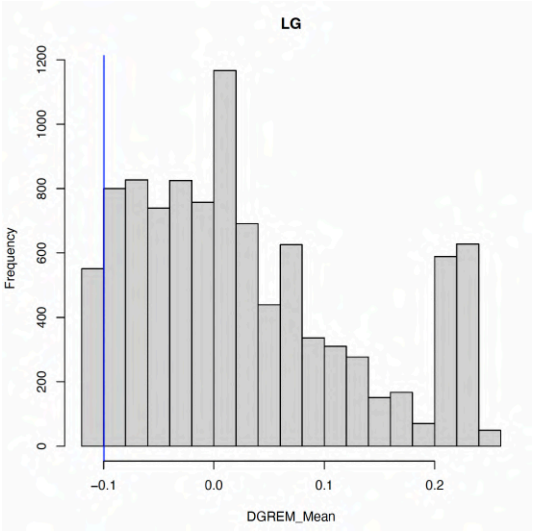

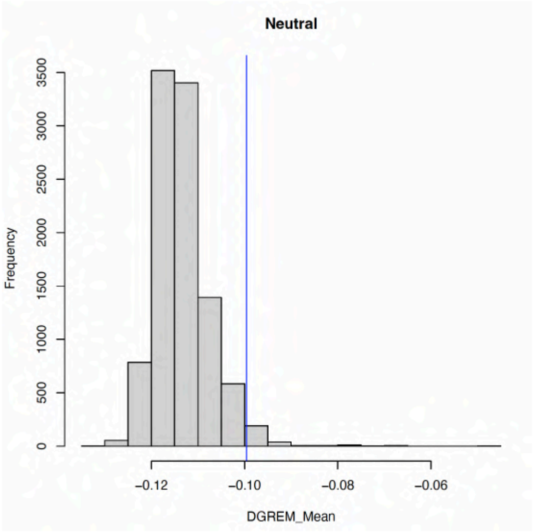

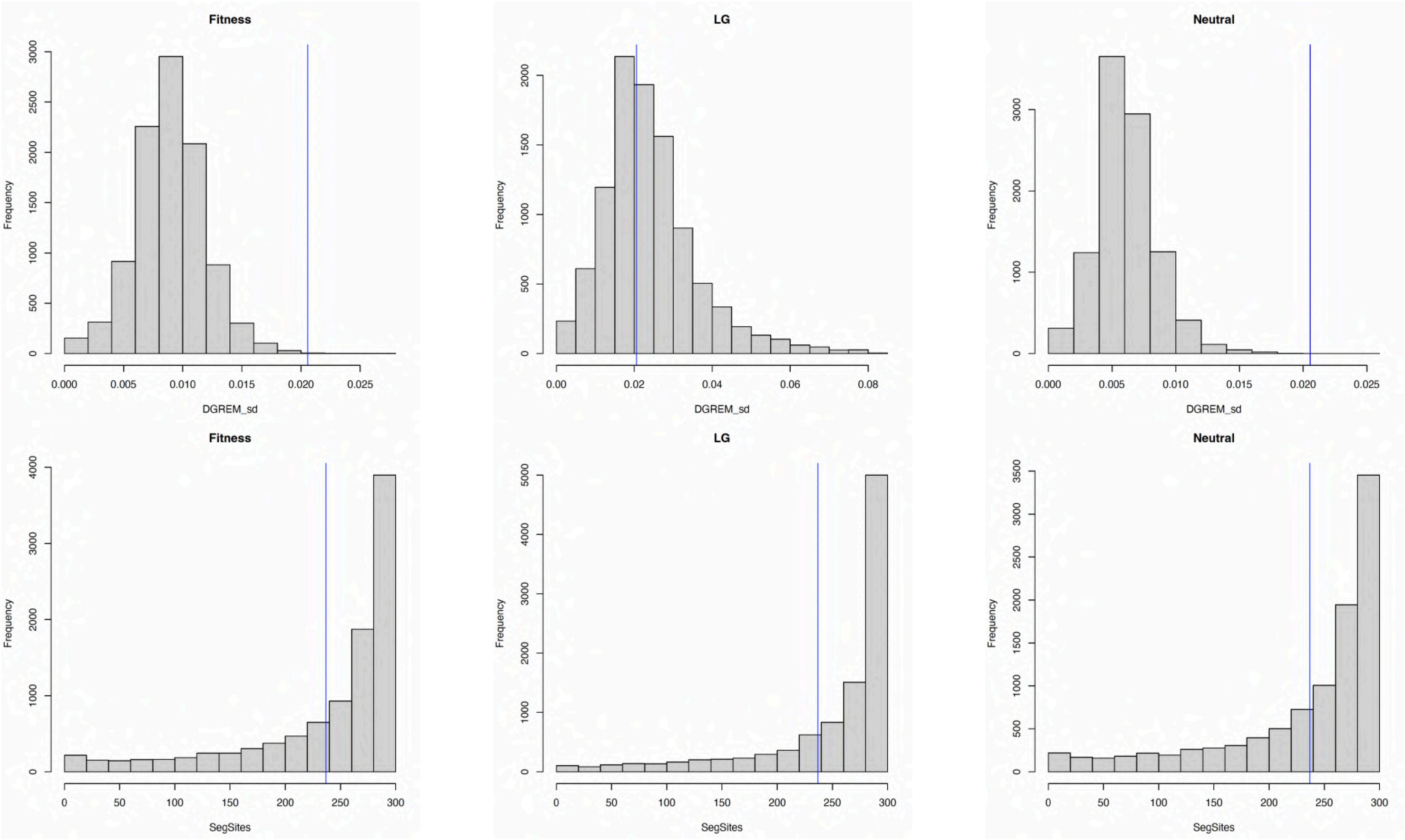
Fitness
LG
Neutral
Frequency
DGREM_sd
SegSites

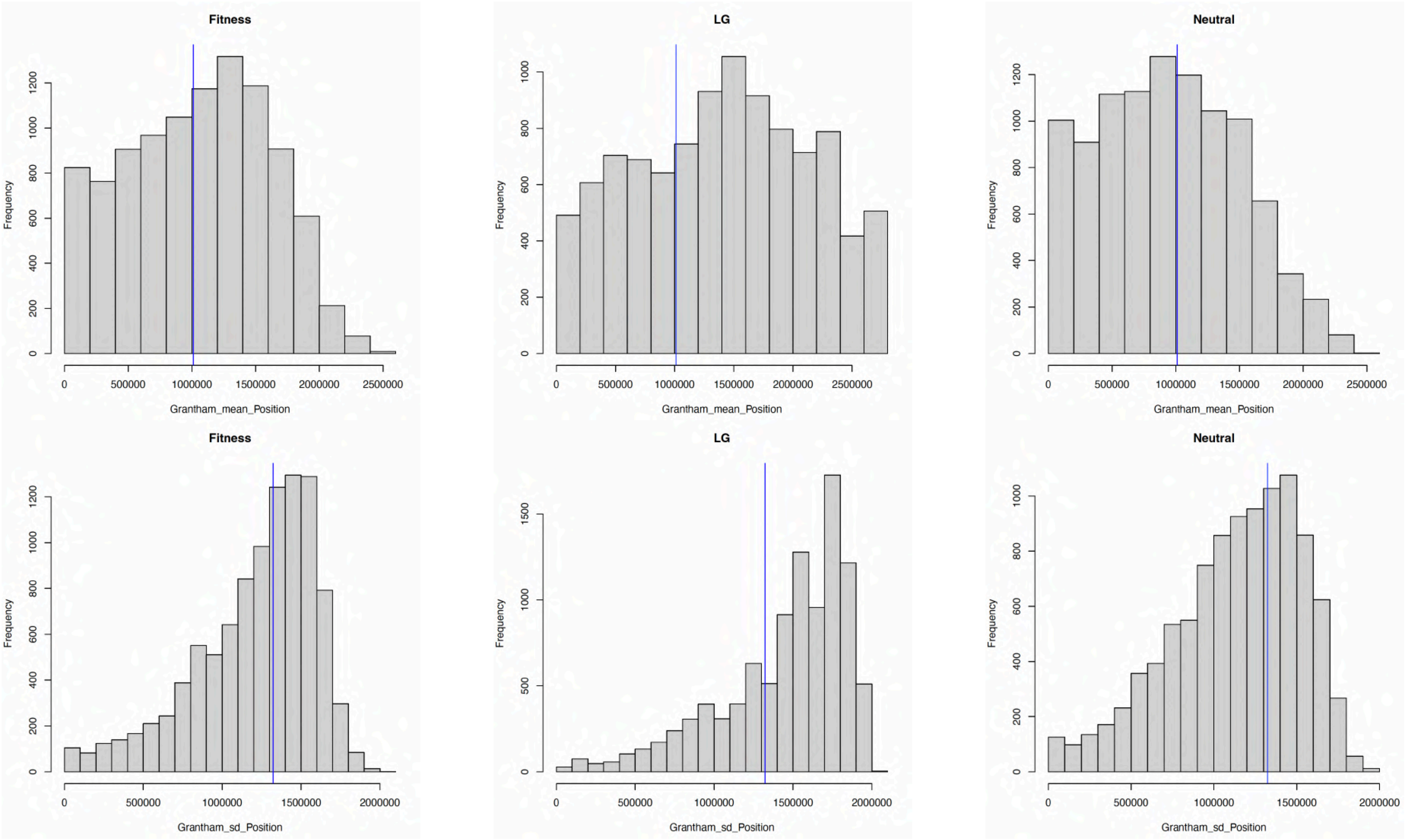
Fitness
Frequency
0
200
400
600
800
1000
1200
0
500000
1000000
1500000
2000000
2500000
Grantham_mean_Position
LG
Frequency
0
200
400
600
800
1000
0
500000
1000000
1500000
2000000
2500000
Grantham_mean_Position
Neutral
Frequency
0
200
400
600
800
1000
1200
0
500000
1000000
1500000
2000000
2500000
Grantham_mean_Position
Fitness
Frequency
0
200
400
600
800
1000
1200
0
500000
1000000
1500000
2000000
Grantham_sd_Position
LG
Frequency
0
500
1000
1500
0
500000
1000000
1500000
2000000
Grantham_sd_Position
Neutral
Frequency
0
200
400
600
800
1000
0
500000
1000000
1500000
2000000
Grantham_sd_Position

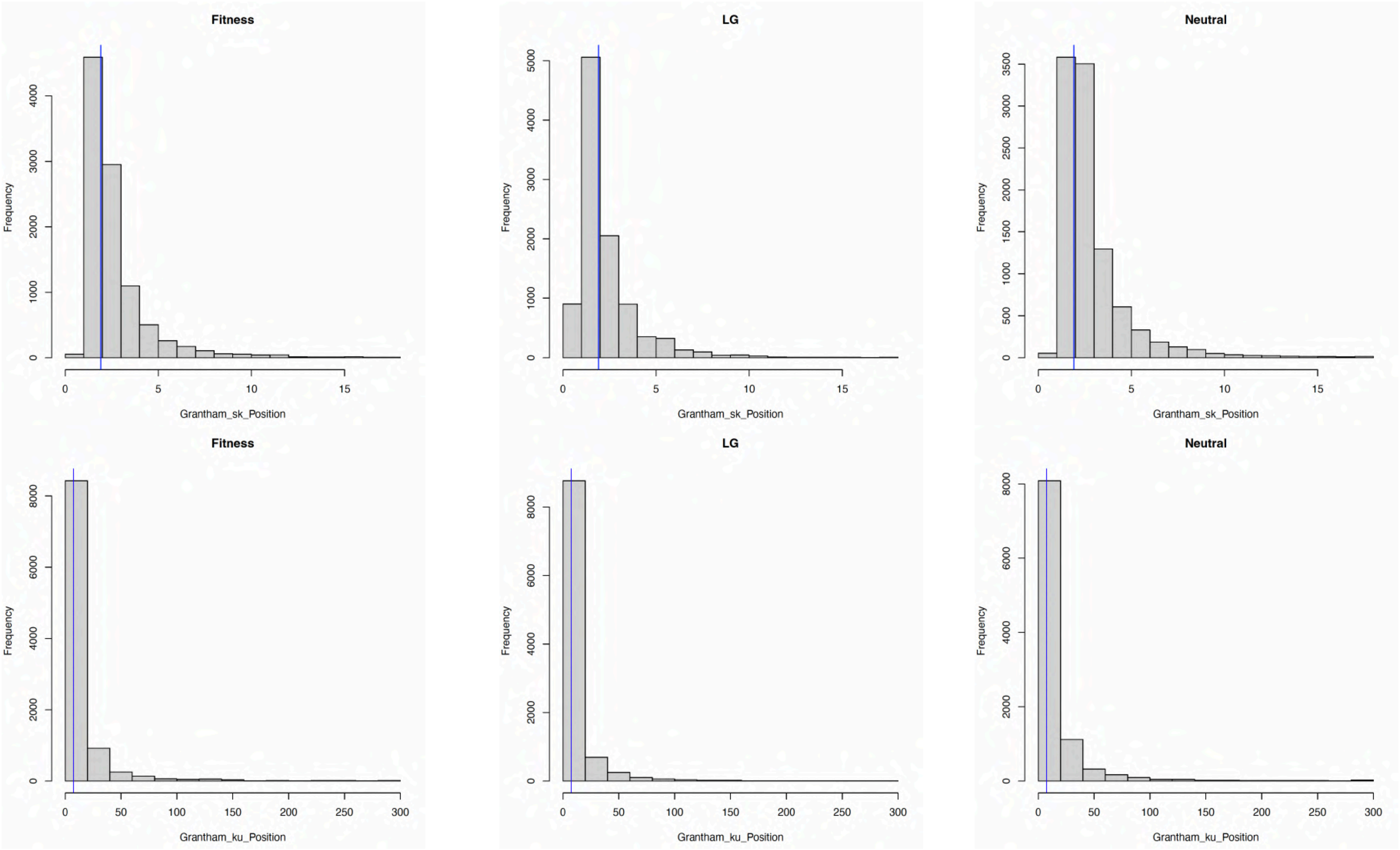
Fitness
LG
Neutral
Frequency
Grantham_sk_Position
Fitness
LG
Neutral
Frequency
Grantham_ku_Position

G. Toll-Interleukin receptor domain protein family

1. Histogram of summary statistics from simulated (under every evaluated substitution model) and real data (blue vertical line).

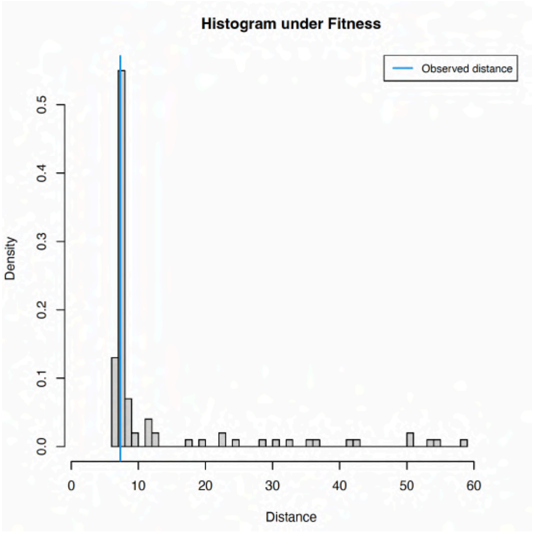

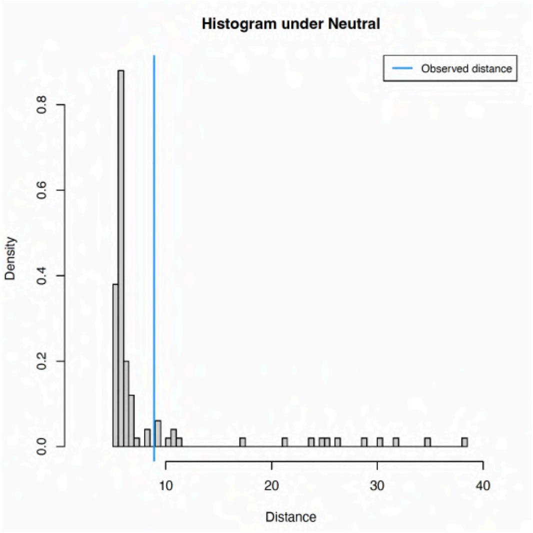

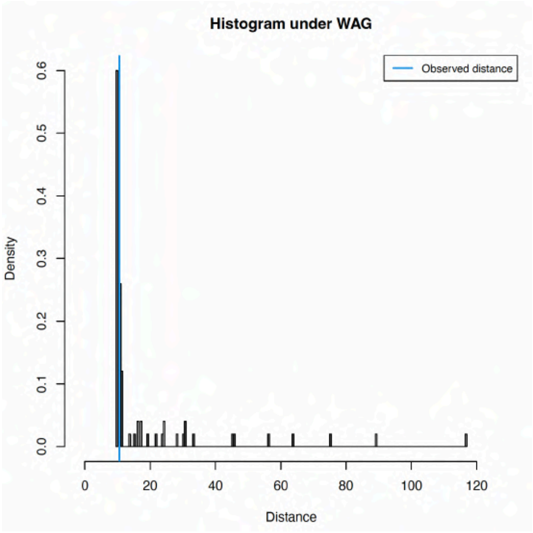

2. Principal component analysis of summary statistics from the data simulated under each substitution model and including summary statistics from the real data (cross).

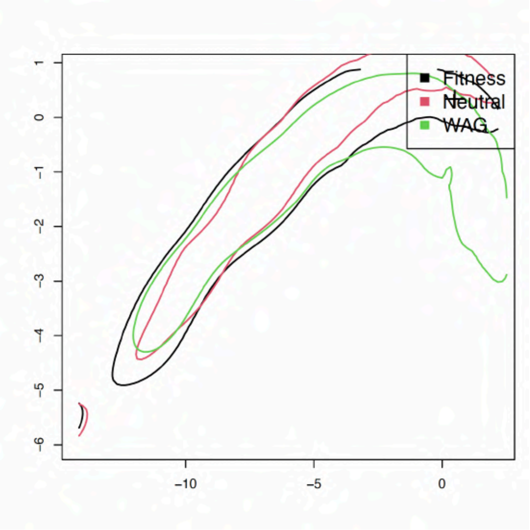

3. Histograms of protein folding stability mean (DGREM_mean) and standard deviation (DGREM_sd), number of segregating sites (SegSites) and Grantham distance mean (Grantham_mean_Position), standard deviation (Grantham_sd_Position), skewness (Grantham_sk_Position) and kurtosis (Grantham_ku_Position), from data simulated under every substitution model and including the corresponding summary statistics from the real data (blue vertical line).

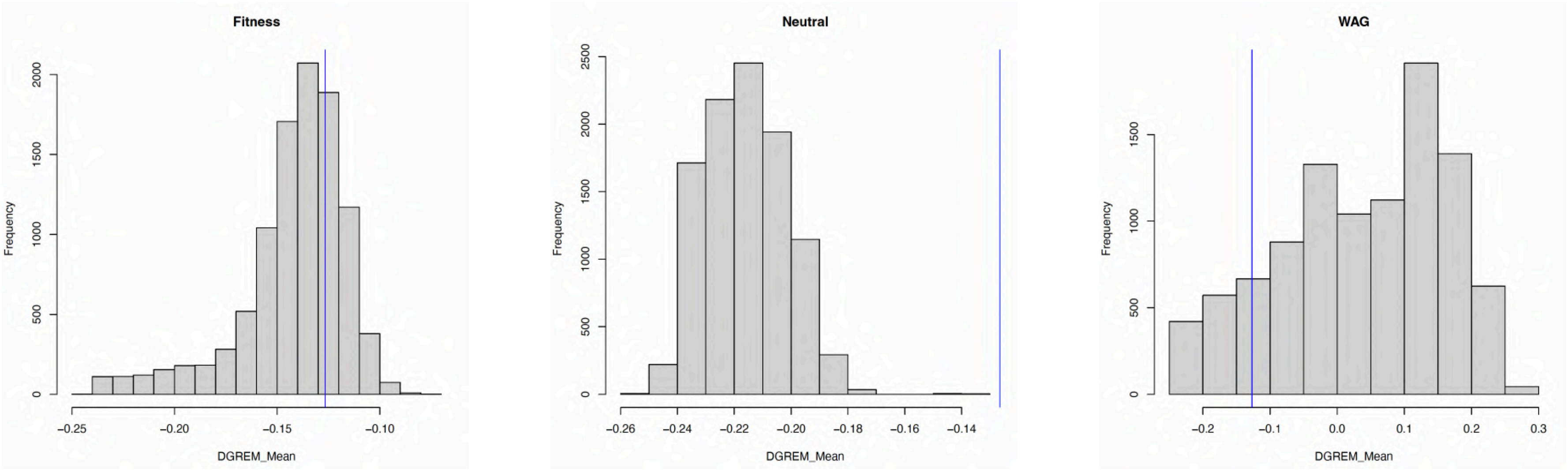

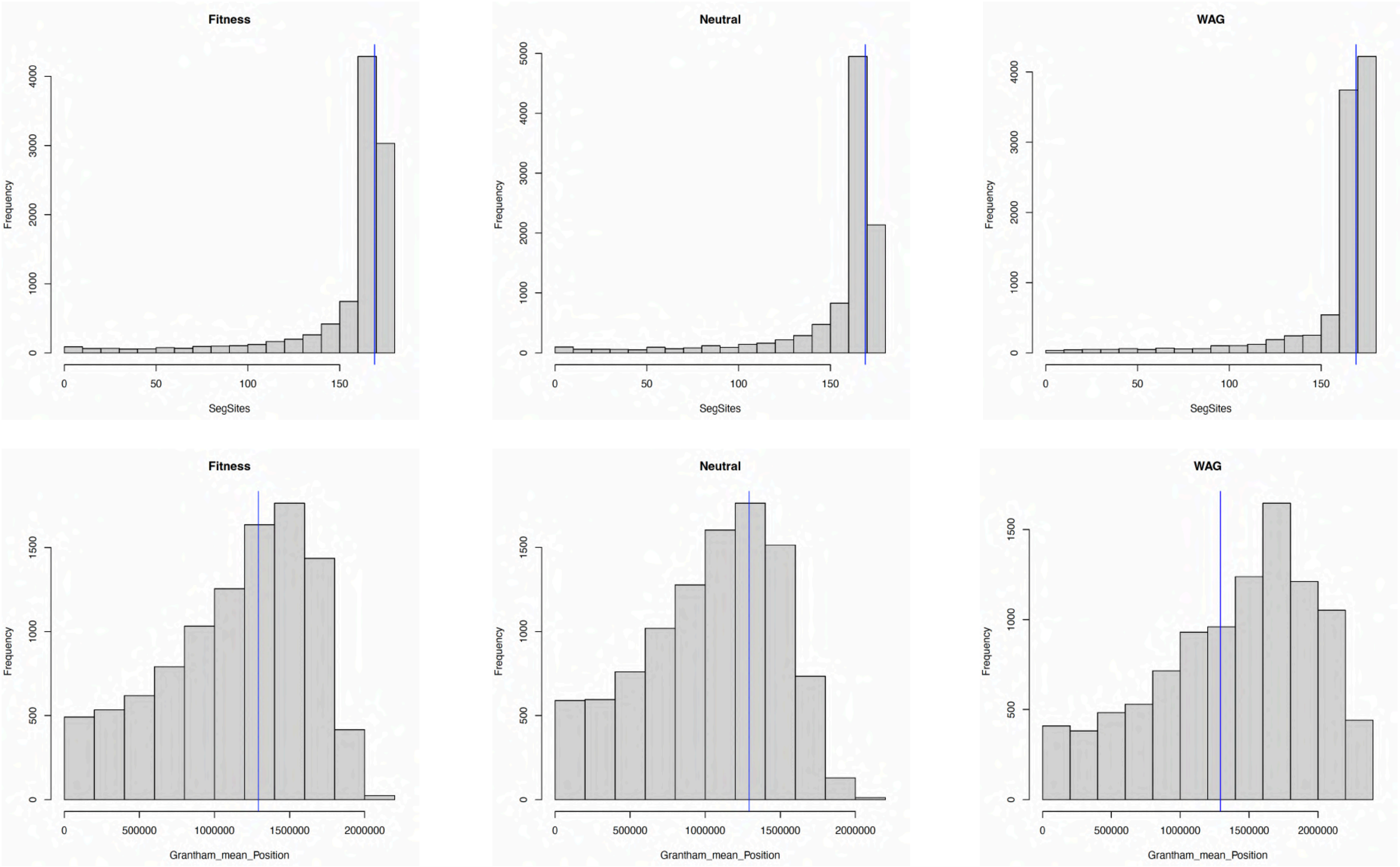
Fitness
Neutral
WAG
Frequency
SegSites
0
50
100
150
0
1000
2000
3000
4000
5000
Fitness
Neutral
WAG
Frequency
Grantham_mean_Position
0
500000
1000000
1500000
2000000
0
500
1000
1500

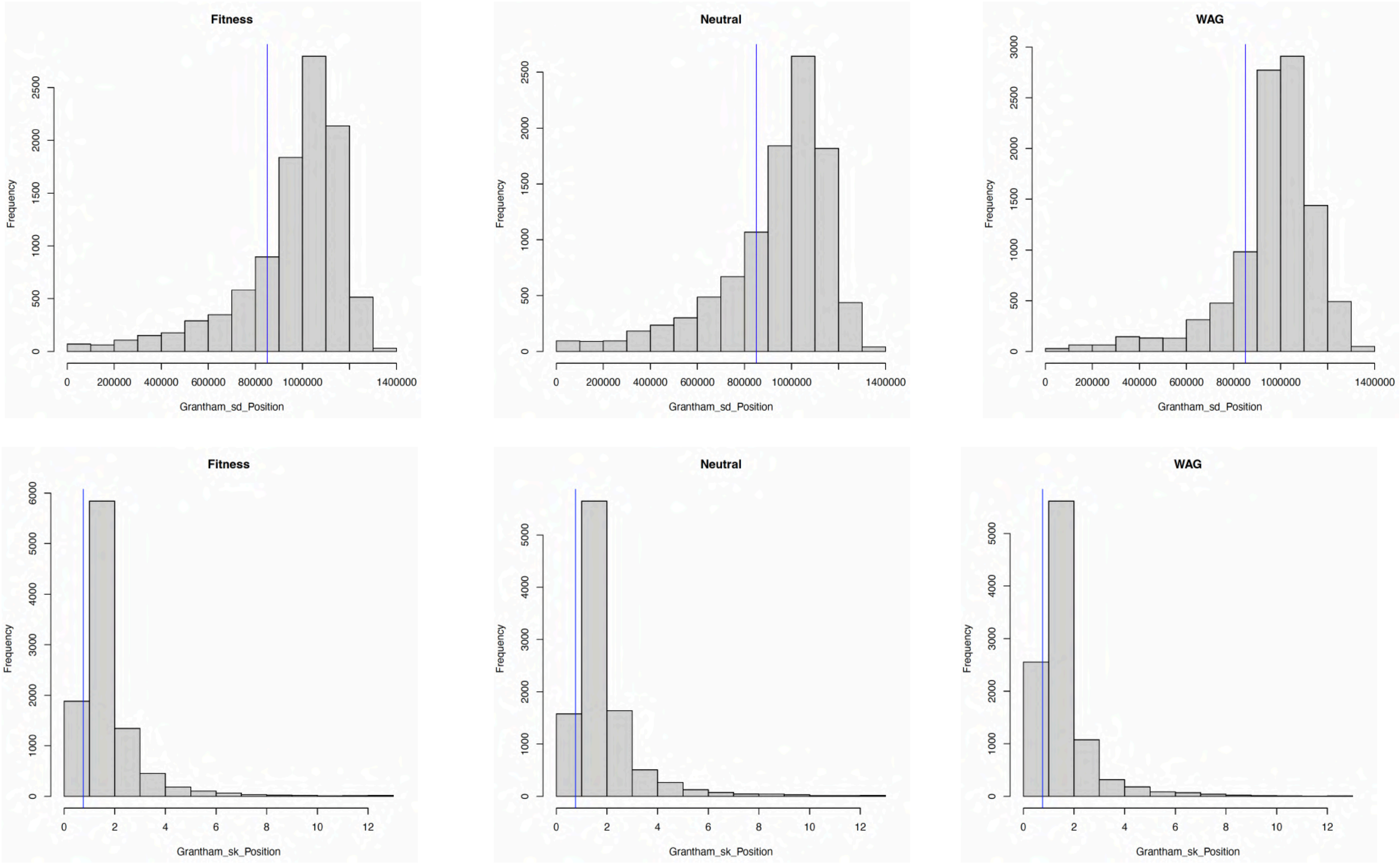
Fitness
Neutral
WAG
Frequency
Grantham_sd_Position
0
200000
400000
600000
800000
1000000
1400000
500
1000
1500
2000
2500
3000
Grantham_sk_Position
0
2
4
6
8
10
12
1000
2000
3000
4000
5000
6000

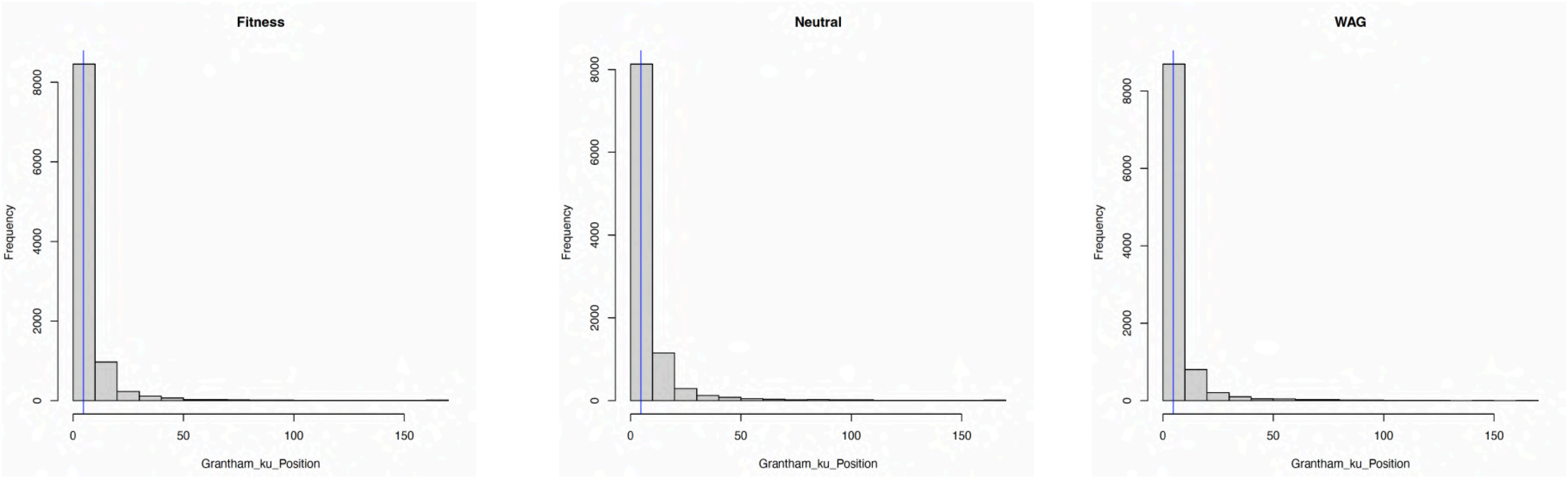
Fitness
Frequency
Grantham_ku_Position
Neutral
Frequency
Grantham_ku_Position
WAG
Frequency
Grantham_ku_Position

H. Mitochondria membrane translocase protein family

1. Histogram of summary statistics from simulated (under every evaluated substitution model) and real data (blue vertical line).

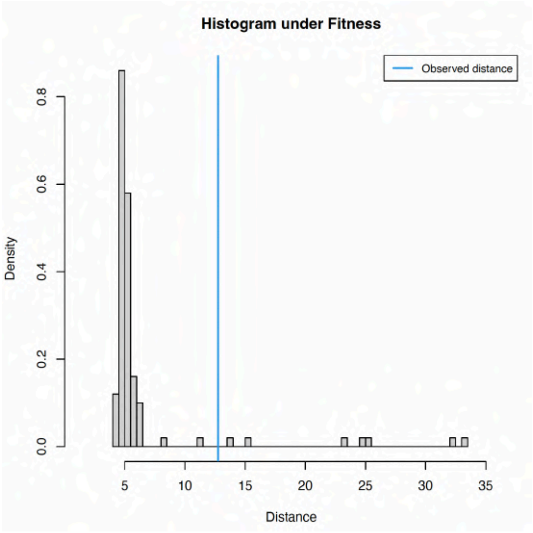

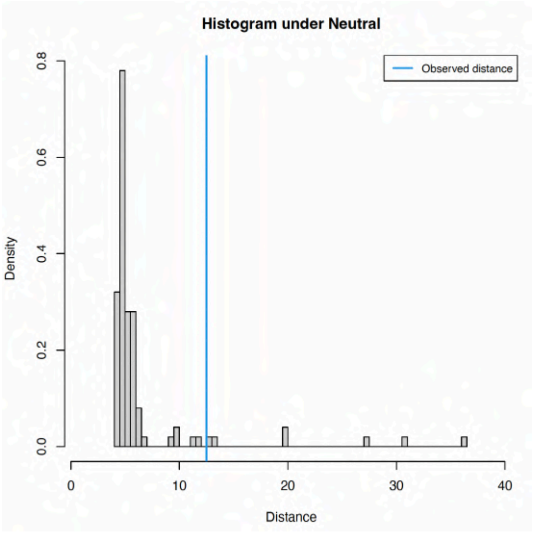

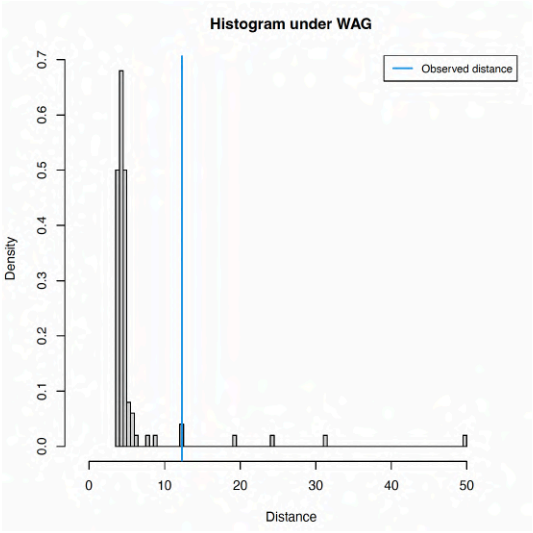

2. Principal component analysis of summary statistics from the data simulated under each substitution model and including summary statistics from the real data (cross).

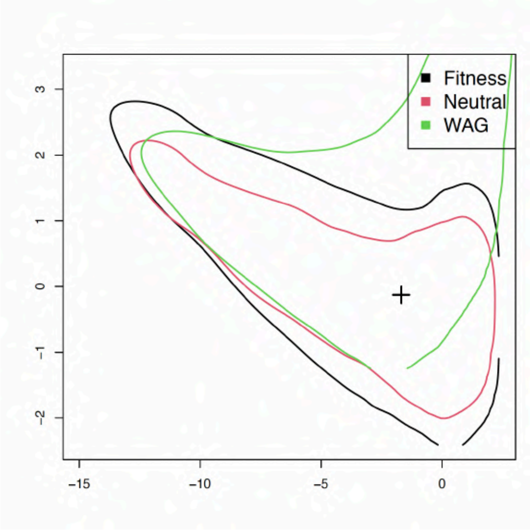

3. Histograms of protein folding stability mean (DGREM_mean) and standard deviation (DGREM_sd), number of segregating sites (SegSites) and Grantham distance mean (Grantham_mean_Position), standard deviation (Grantham_sd_Position), skewness (Grantham_sk_Position) and kurtosis (Grantham_ku_Position), from data simulated under every substitution model and including the corresponding summary statistics from the real data (blue vertical line).

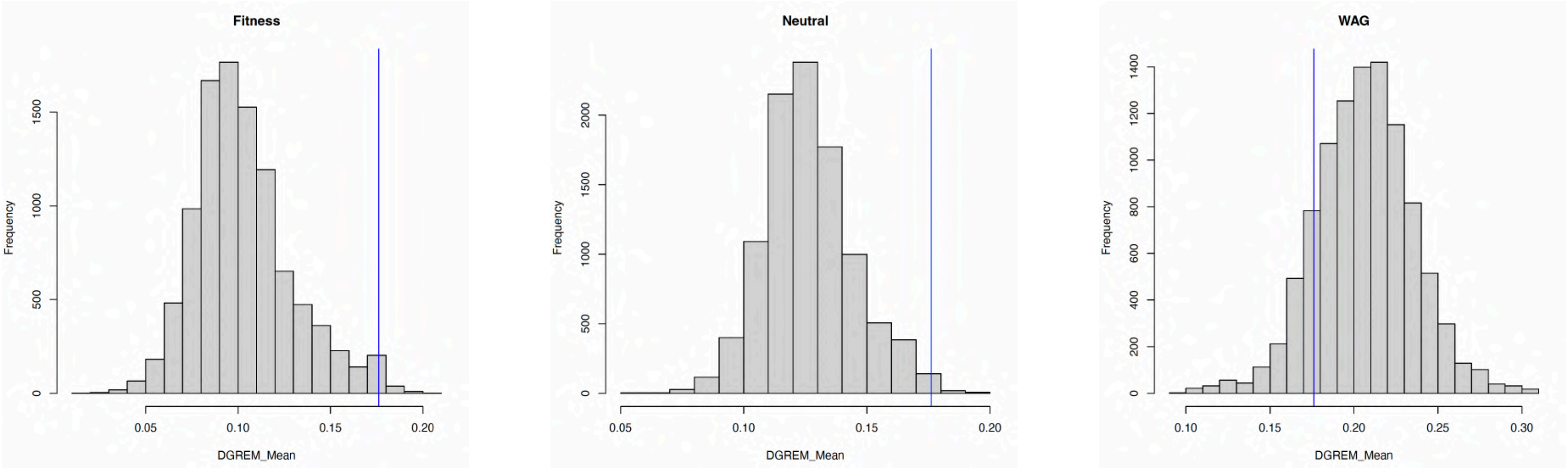

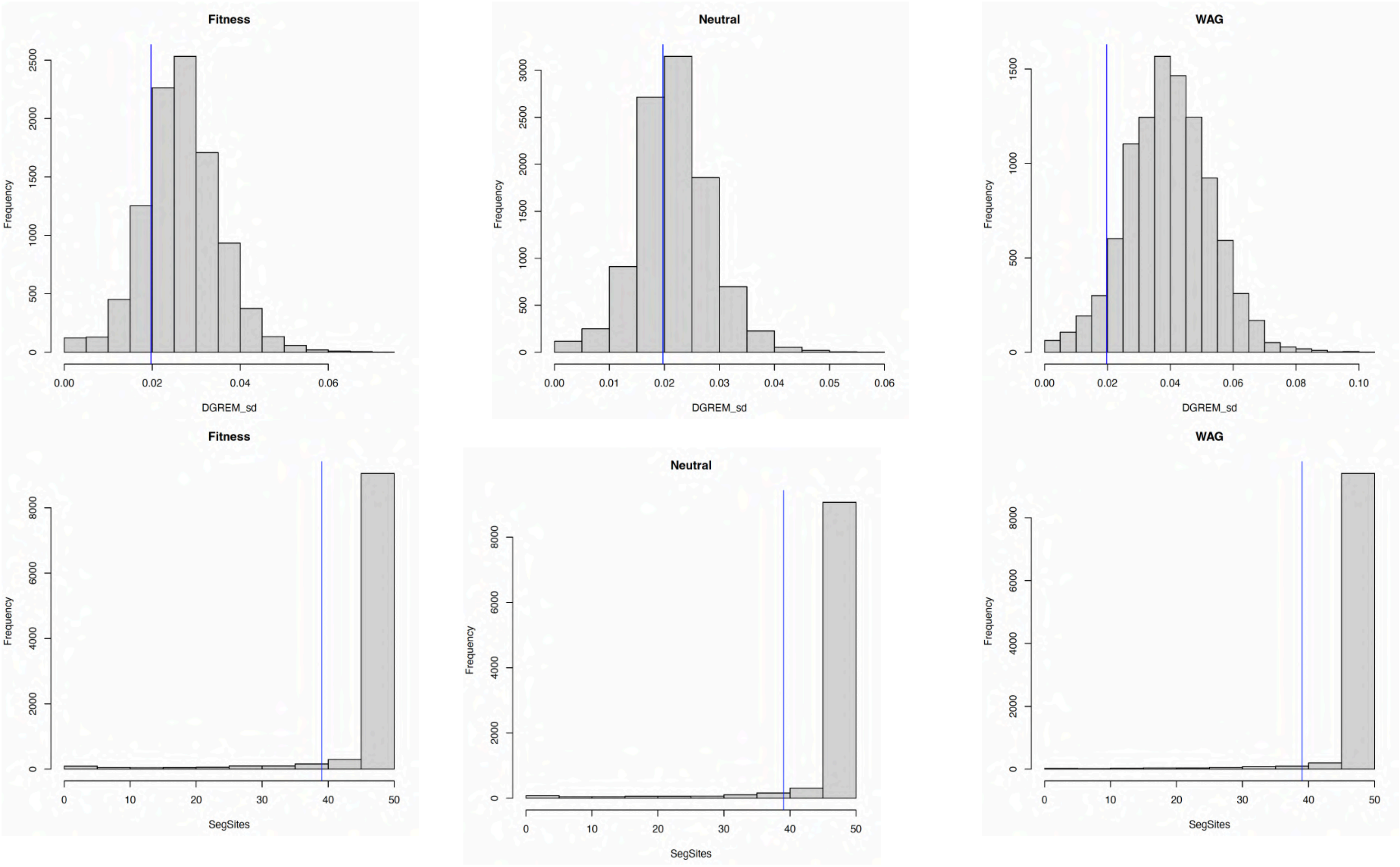
Fitness
Frequency
0
500
1000
1500
2000
2500
0.00
0.02
0.04
0.06
DGREM_sd
Neutral
Frequency
0
500
1000
1500
2000
2500
3000
0.00
0.01
0.02
0.03
0.04
0.05
0.06
DGREM_sd
WAG
Frequency
0
500
1000
1500
0.00
0.02
0.04
0.06
0.08
0.10
DGREM_sd
Fitness
Frequency
0
2000
4000
6000
8000
0
10
20
30
40
50
SegSites
Neutral
Frequency
0
2000
4000
6000
8000
0
10
20
30
40
50
SegSites
WAG
Frequency
0
2000
4000
6000
8000
0
10
20
30
40
50
SegSites

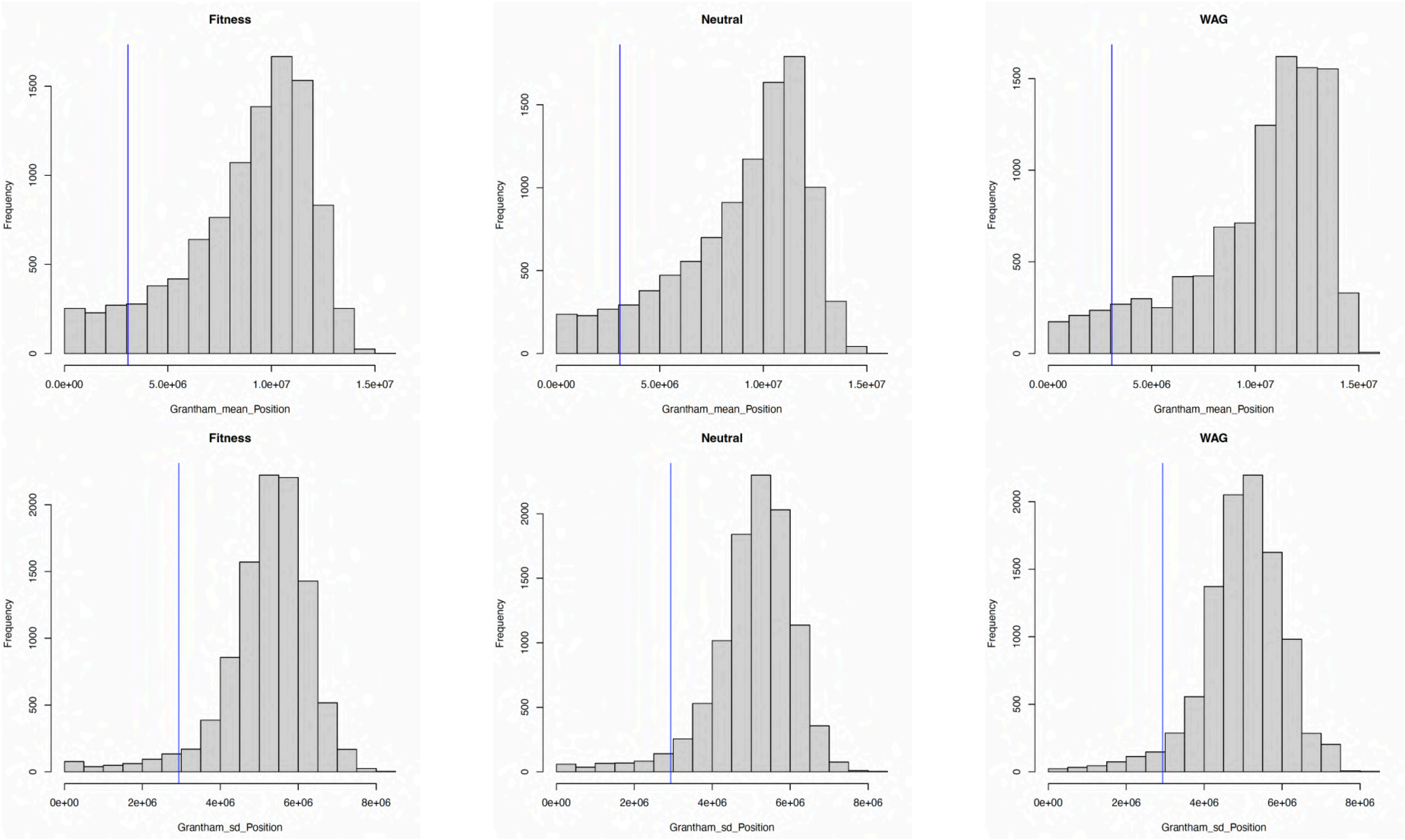
Fitness
Neutral
WAG
Frequency
Grantham_mean_Position
0.0e+00
5.0e+06
1.0e+07
1.5e+07
0
500
1000
1500
Grantham_sd_Position
0e+00
2e+06
4e+06
6e+06
8e+06
2000

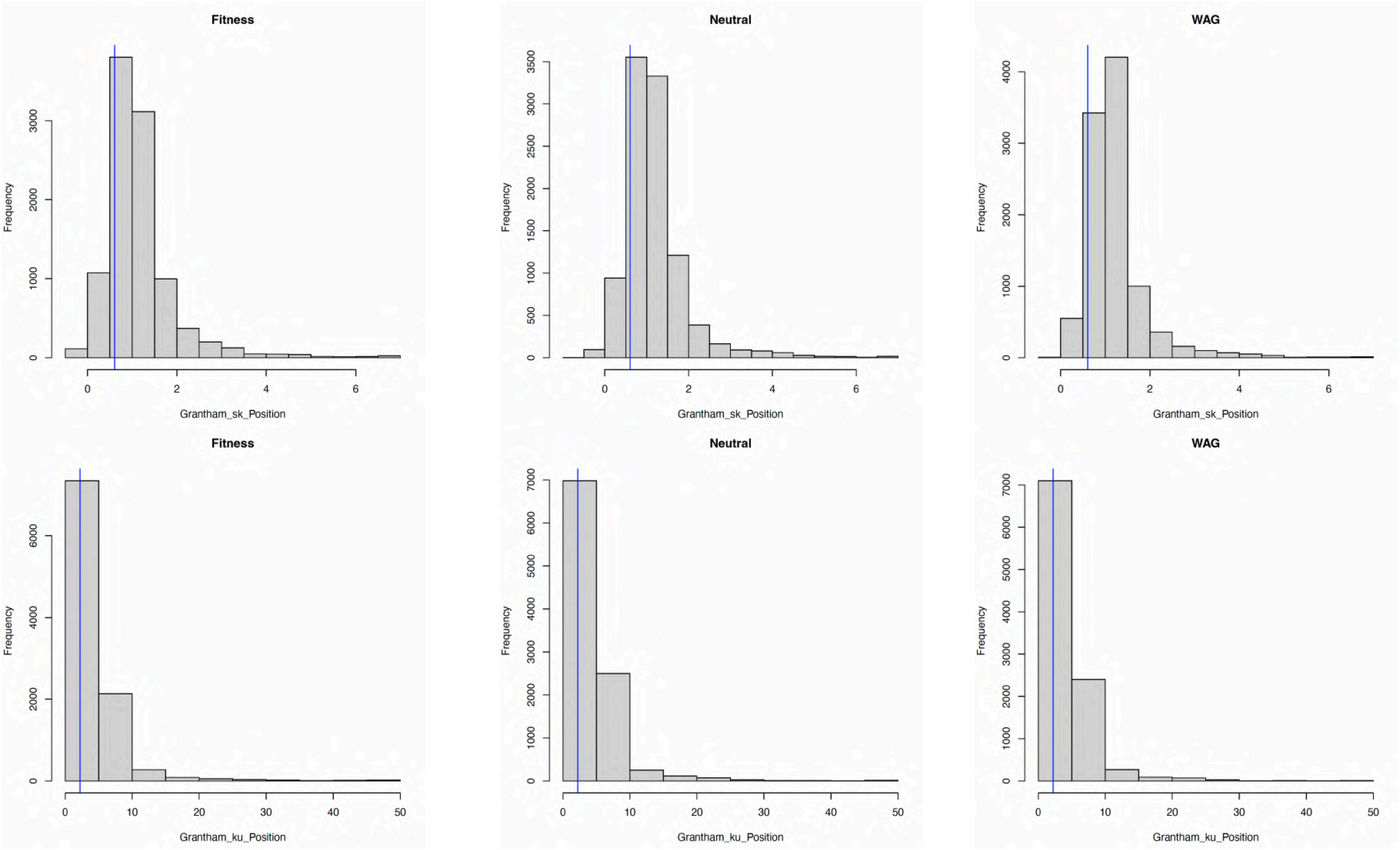
Fitness
Neutral
WAG
Frequency
Grantham_sk_Position
Fitness
Neutral
WAG
Frequency
Grantham_ku_Position

I. Squalene epoxidase protein family

1. Histogram of summary statistics from simulated (under every evaluated substitution model) and real data (blue vertical line).

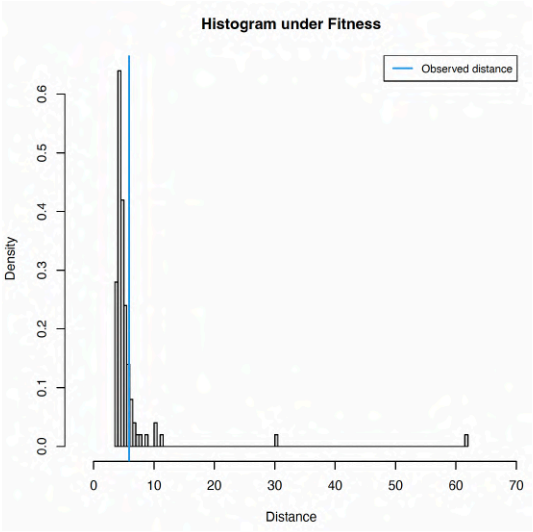

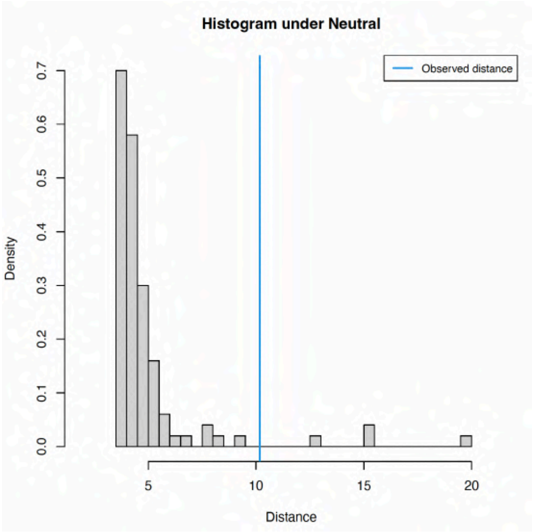

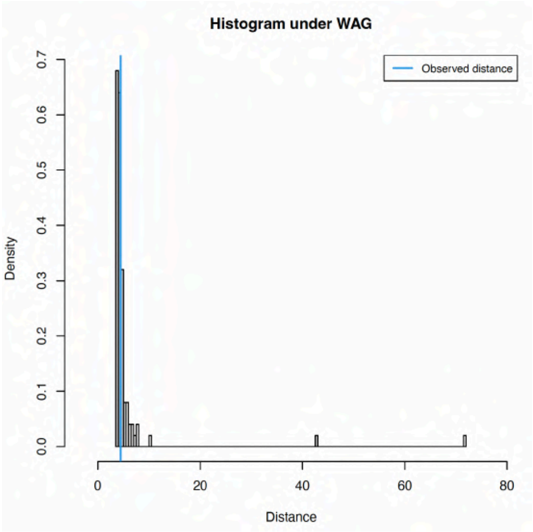

2. Principal component analysis of summary statistics from the data simulated under each substitution model and including summary statistics from the real data (cross).

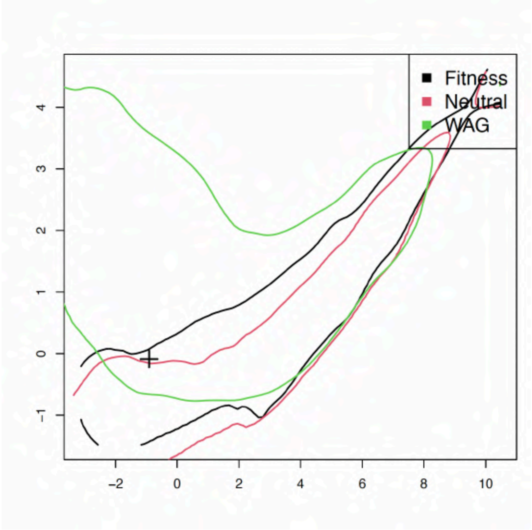

3. Histograms of protein folding stability mean (DGREM_mean) and standard deviation (DGREM_sd), number of segregating sites (SegSites) and Grantham distance mean (Grantham_mean_Position), standard deviation (Grantham_sd_Position), skewness (Grantham_sk_Position) and kurtosis (Grantham_ku_Position), from data simulated under every substitution model and including the corresponding summary statistics from the real data (blue vertical line).

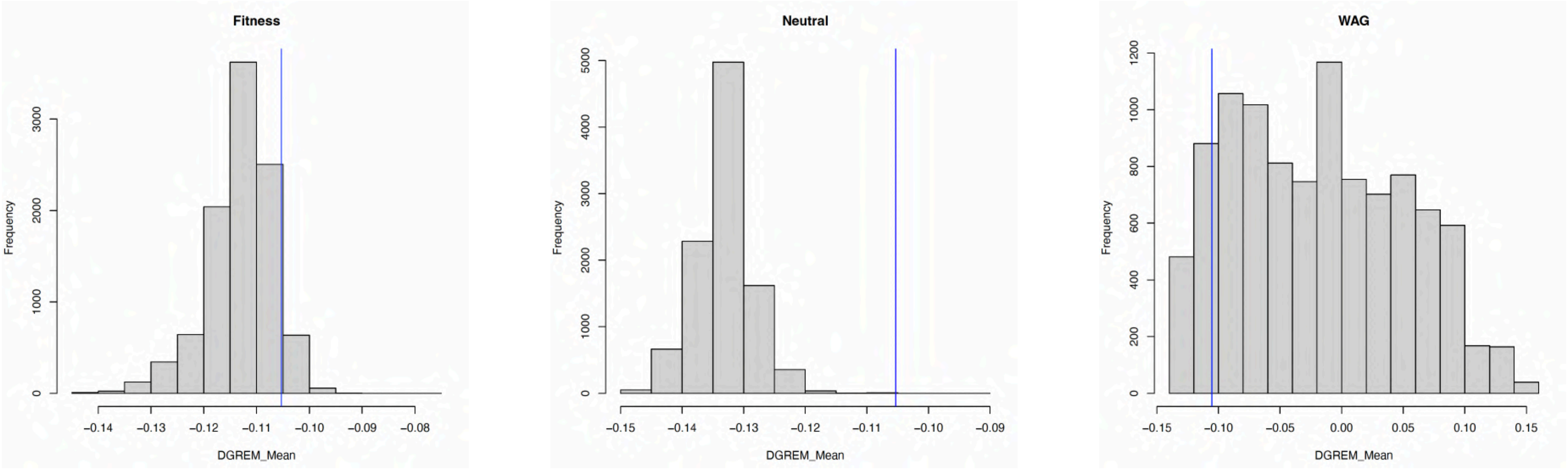

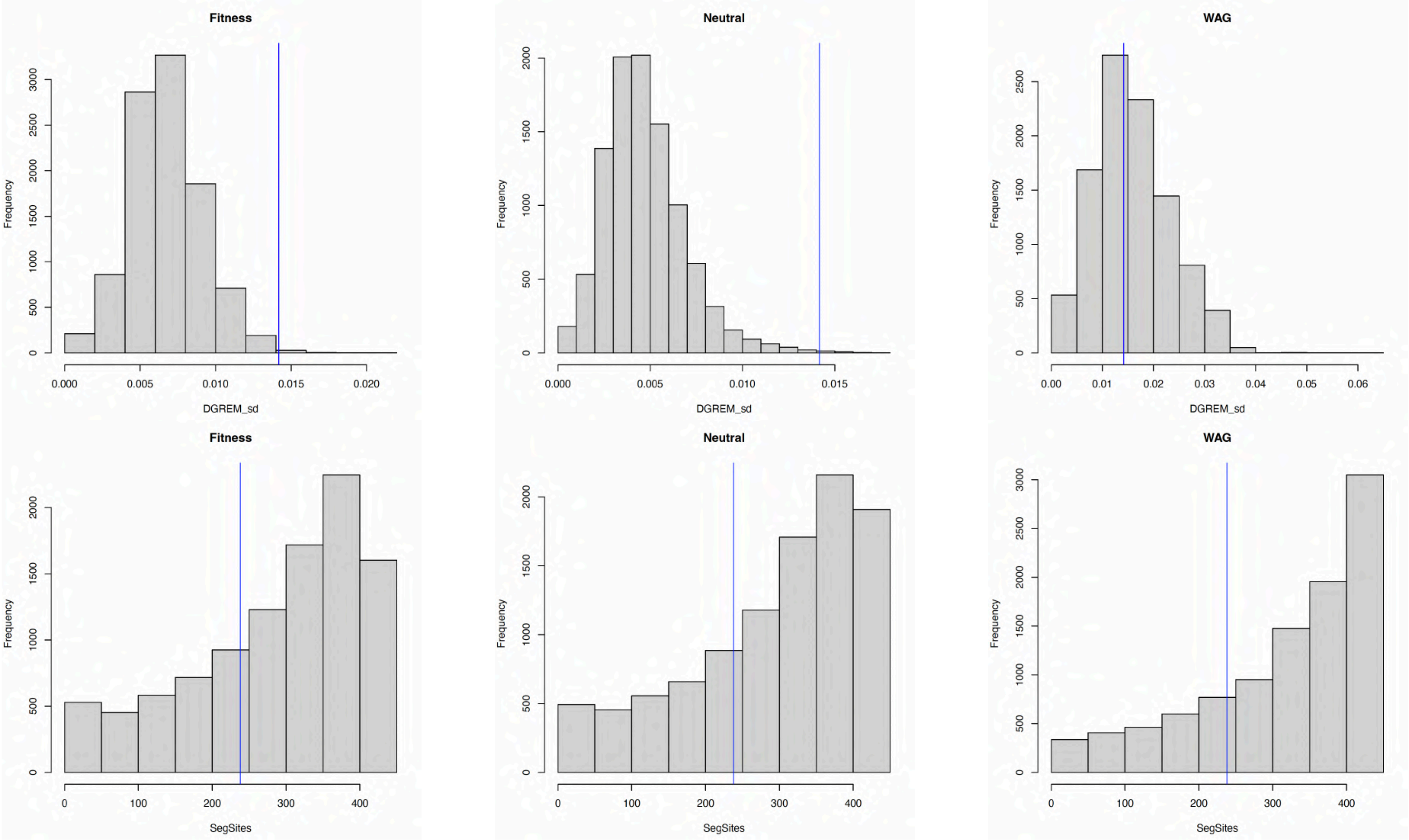

Fitness
Neutral
WAG
Frequency
DGREM_sd
0.000
0.005
0.010
0.015
0.020
0.00
0.01
0.02
0.03
0.04
0.05
0.06
SegSites
0
100
200
300
400
500
1000
1500
2000
2500
3000

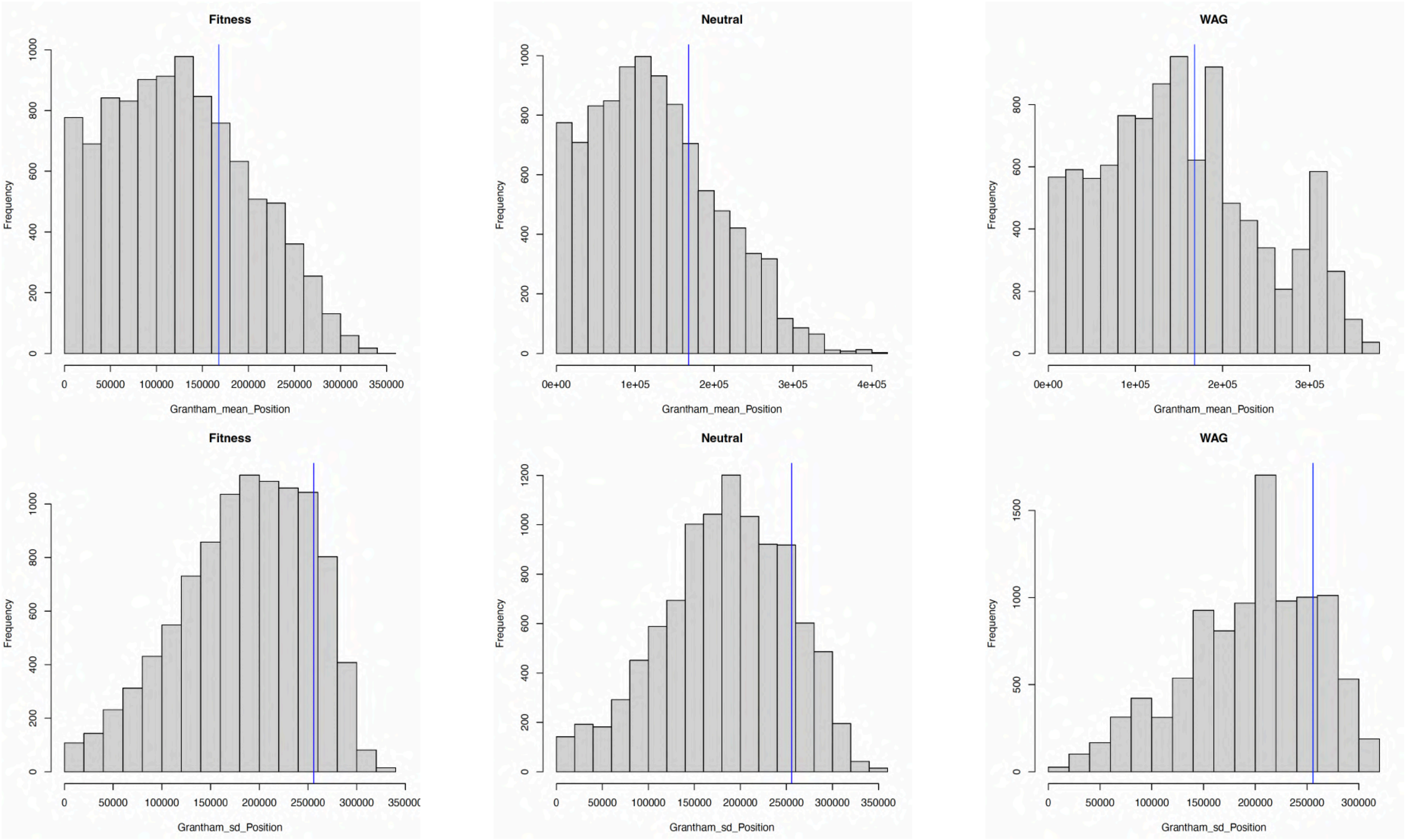
Fitness
Neutral
WAG
Frequency
Grantham_mean_Position
Grantham_sd_Position

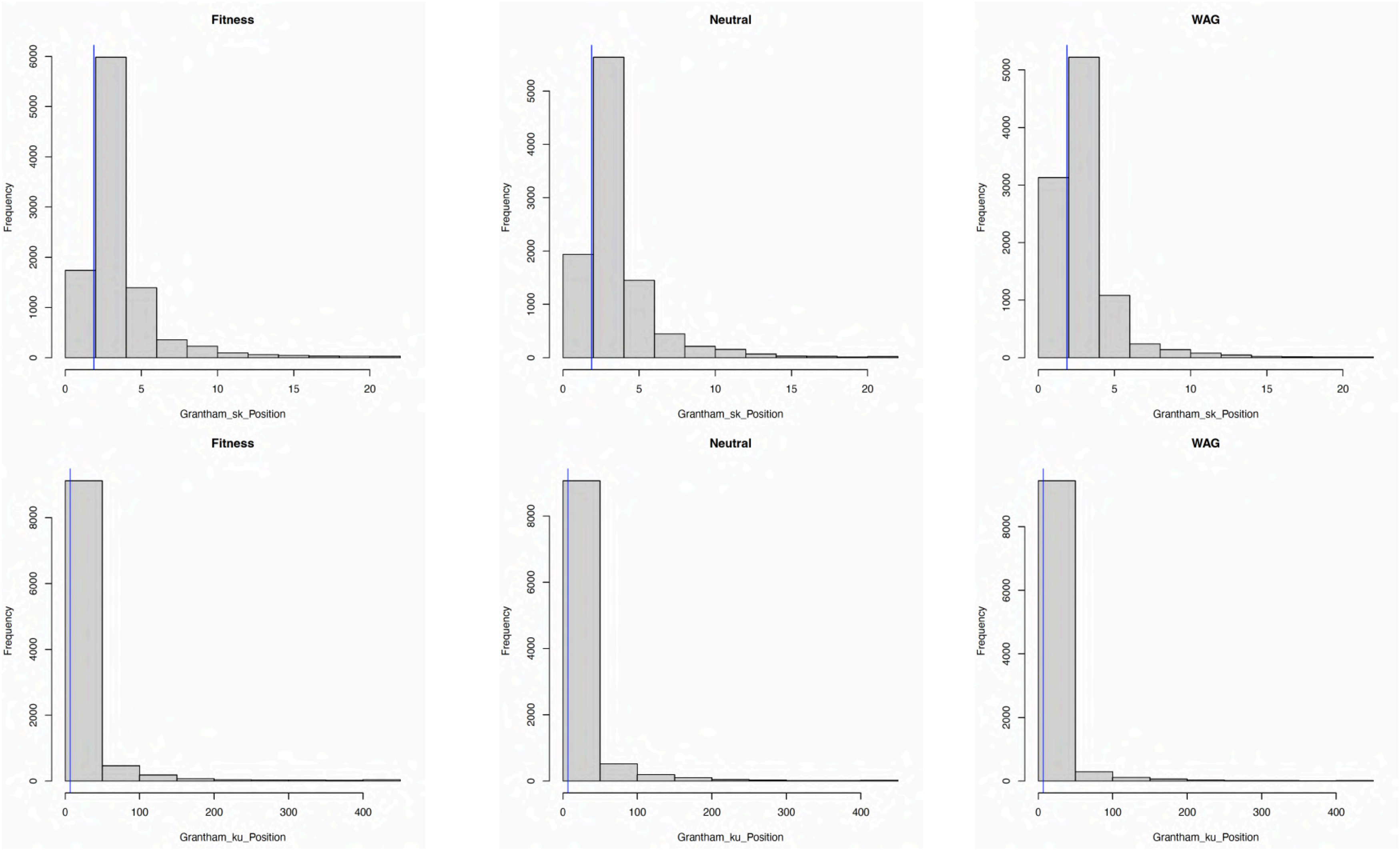
Fitness
Neutral
WAG
Frequency
Grantham_sk_Position
Grantham_ku_Position
0
5
10
15
20
100
200
300
400
1000
2000
3000
4000
5000
6000
8000

J. Ebola nucleoprotein protein family

1. Histogram of summary statistics from simulated (under every evaluated substitution model) and real data (blue vertical line).

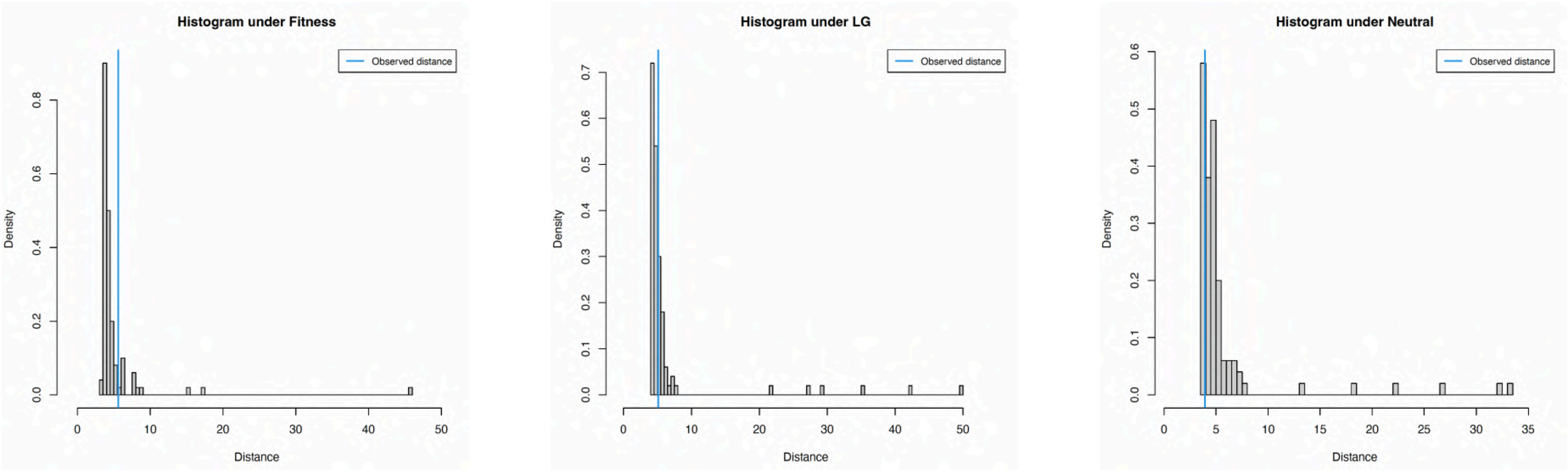

2. Principal component analysis of summary statistics from the data simulated under each substitution model and including summary statistics from the real data (cross).

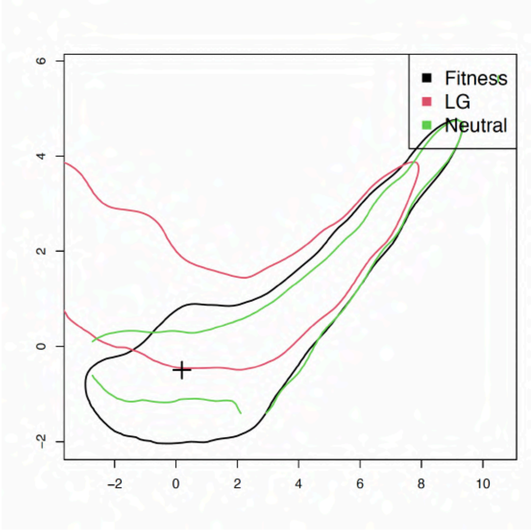

3. Histograms of protein folding stability mean (DGREM_mean) and standard deviation (DGREM_sd), number of segregating sites (SegSites) and Grantham distance mean (Grantham_mean_Position), standard deviation (Grantham_sd_Position), skewness (Grantham_sk_Position) and kurtosis (Grantham_ku_Position), from data simulated under every substitution model and including the corresponding summary statistics from the real data (blue vertical line).

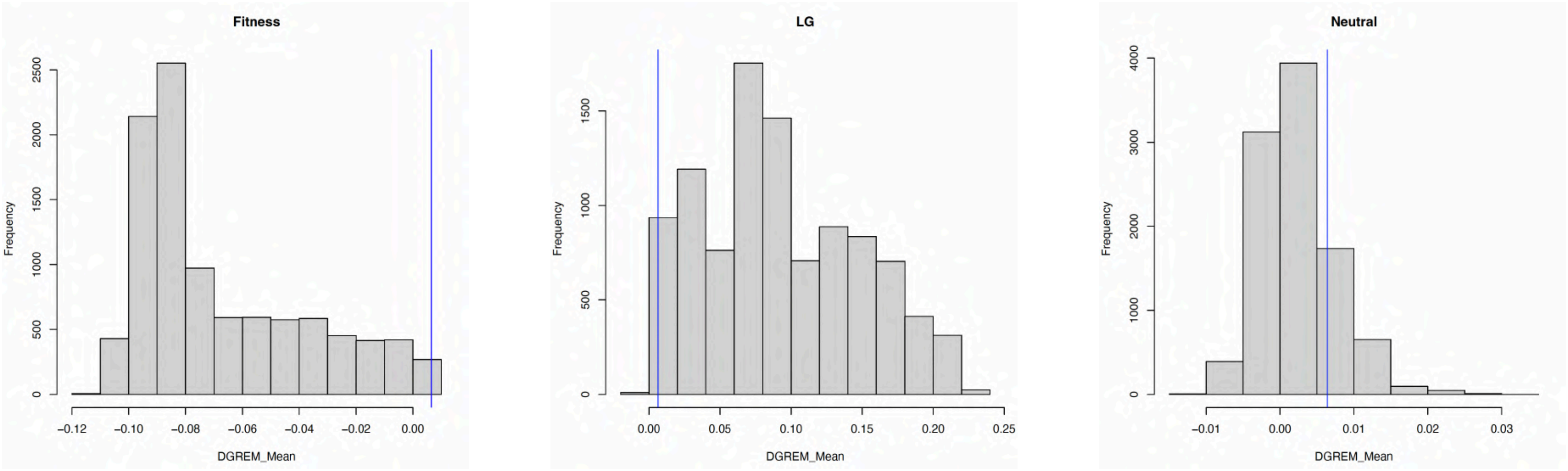

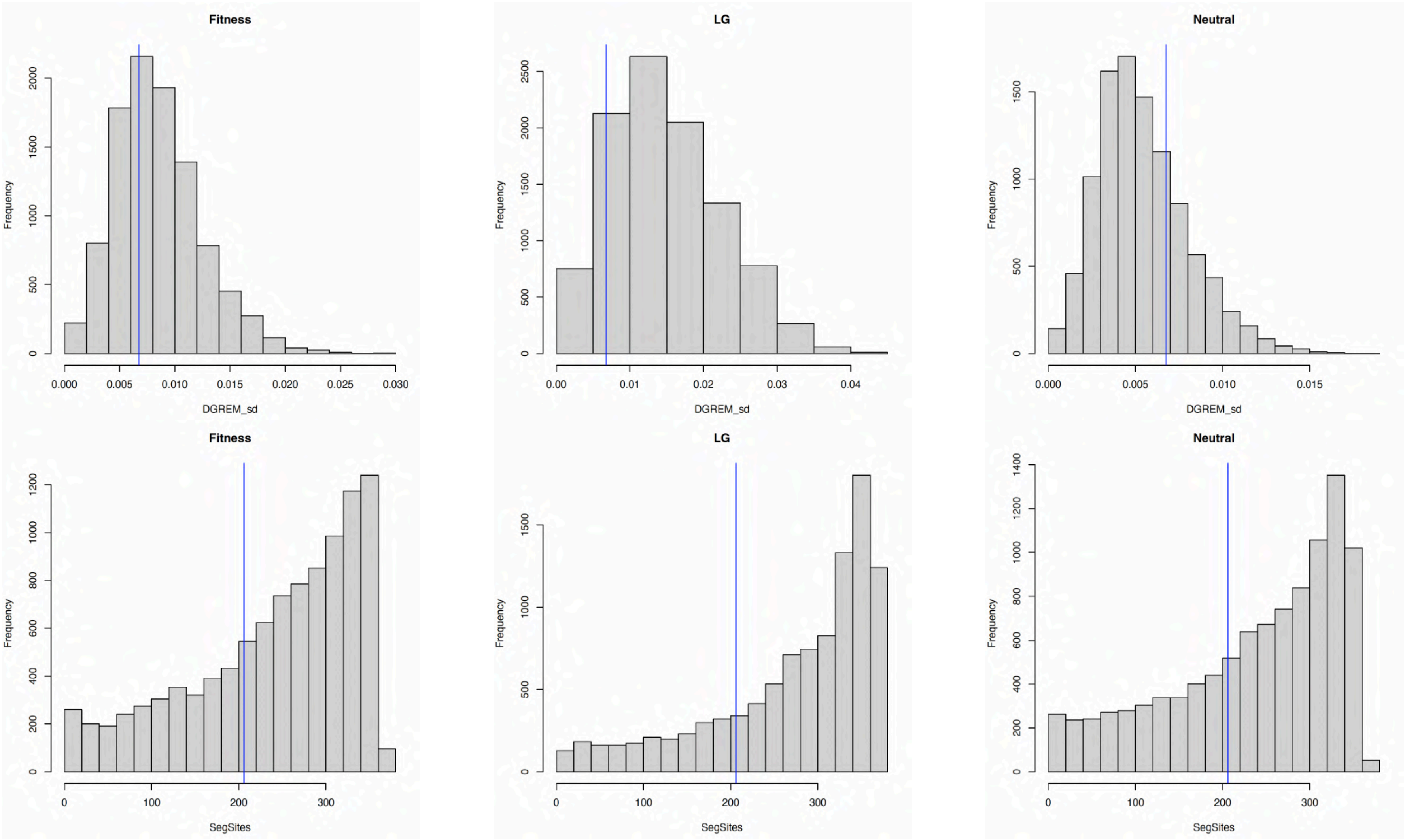
Fitness
LG
Neutral
Frequency
DGREM_sd
SegSites

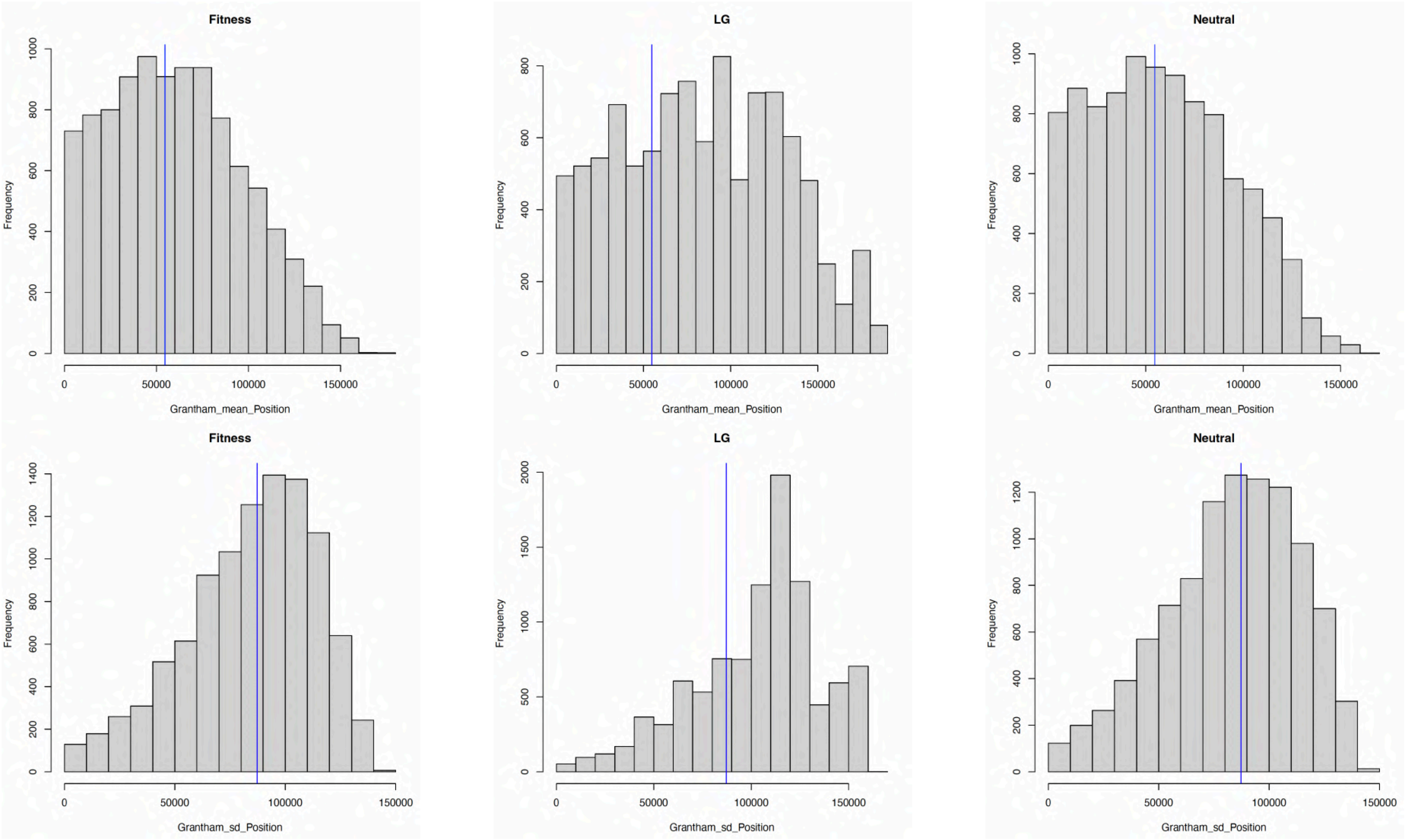
Fitness
LG
Neutral
Frequency
Grantham_mean_Position
Grantham_sd_Position
0
50000
100000
150000

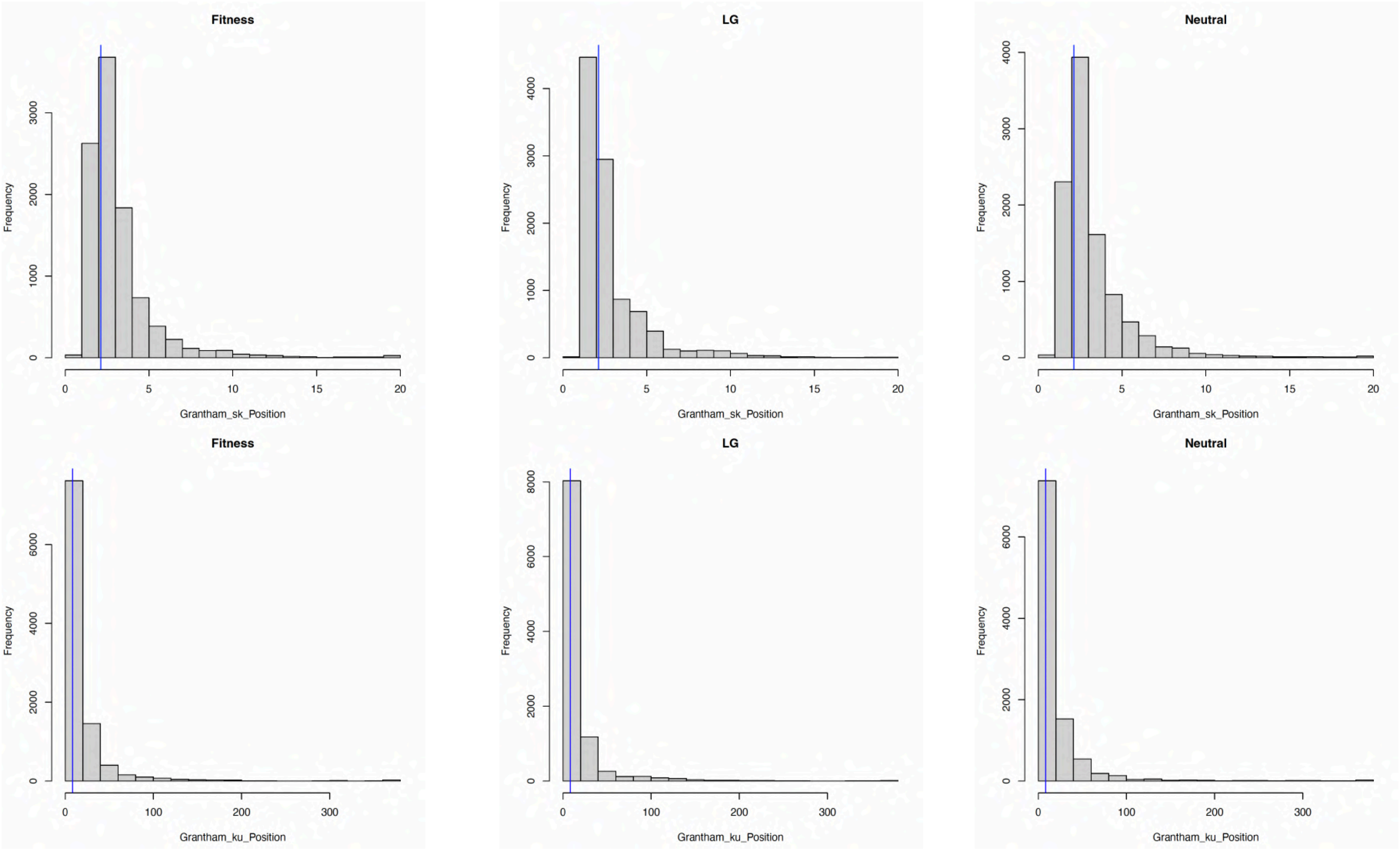
Fitness
LG
Neutral
Frequency
0
1000
2000
3000
4000
0
5
10
15
20
Grantham_sk_Position
Fitness
LG
Neutral
Frequency
0
2000
4000
6000
8000
0
100
200
300
Grantham_ku_Position
